# Invisible no more: a scoping review of the health care aide workforce literature

**DOI:** 10.1186/s12912-015-0090-x

**Published:** 2015-07-22

**Authors:** Sarah J. Hewko, Sarah L. Cooper, Hanhmi Huynh, Trish L. Spiwek, Heather L. Carleton, Shawna Reid, Greta G. Cummings

**Affiliations:** CLEAR Outcomes Research Program, Faculty of Nursing, University of Alberta, Level 3, Edmonton Clinic Health Academy, 11405 87 Avenue, Edmonton, AB T6G 1C9 Canada

**Keywords:** Health manpower, Review, Home health aides, Nurses’ aides, Frail elderly, Nursing homes, Home care services, Health services accessibility

## Abstract

**Background:**

Healthcare aides (HCAs) are the primary caregivers for vulnerable older persons. They have many titles and are largely unregulated, which contributes to their relative invisibility. The objective of this scoping review was to evaluate the breadth and depth of the HCA workforce literature.

**Methods:**

We conducted a search of seven online bibliographic databases. Studies were included if published since 1995 in English, peer-reviewed journals. Results were iteratively synthesized within and across the following five categories: education, supply, use, demand and injury and illness.

**Results:**

Of 5,045 citations screened, 82 studies met inclusion criteria. Few examined HCA *education*; particularly trainee characteristics, program location, length and content. Results in *supply* indicated that the average HCA was female, 36–45 years and had an education level of high school or less. Home health HCAs were, on average, older and were more likely to be immigrants than those working in other settings. The review of studies exploring HCA *use* revealed that their role was unclear – variation in duties, level of autonomy and work setting make describing “the” role of an HCA near impossible. Projected increased *demand* for HCAs and high rates of turnover, both at the profession and facility-level, elicit predictions of future HCA shortages. Home health HCAs experienced comparatively lower job stability, earned less, worked the fewest hours and were less likely to have fringe benefits than HCAs employed in hospitals and nursing homes. The review of studies related to HCA *illness and injury* revealed that they were at comparatively higher risk of injury than registered nurses and licensed practical nurses.

**Conclusions:**

This is the largest, most comprehensive scoping review of HCA workforce literature to date. Our results indicate that the HCA workforce is both invisible and ubiquitous; as long as this is the case, governments and healthcare organizations will be limited in their ability to develop and implement feasible, effective HCA workforce plans. The continued undervaluation of HCAs adversely impacts care providers, the institutions they work for and those who depend on their care. Future workforce planning and research necessitates national HCA registries, or at minimum, directories.

**Electronic supplementary material:**

The online version of this article (doi:10.1186/s12912-015-0090-x) contains supplementary material, which is available to authorized users.

## Background

**Fig. 1:**
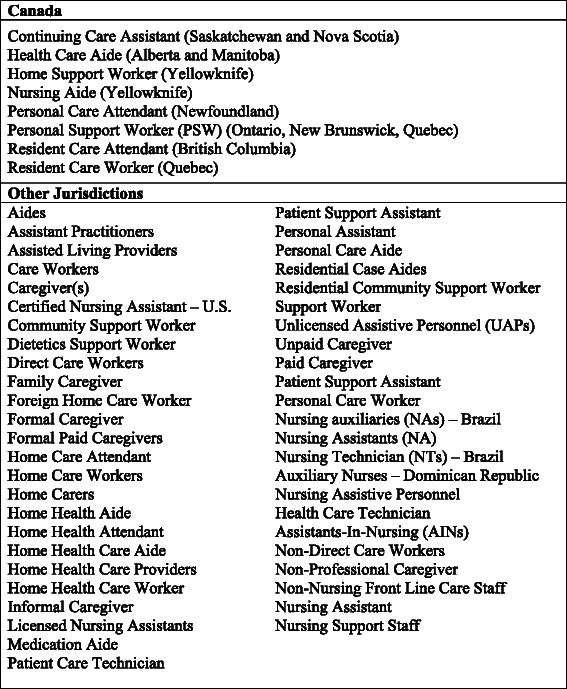
Alternate titles for healthcare aides

Healthcare aides (HCAs) go by many titles and are largely unregulated, which contributes to the relative invisibility of this workforce in the eyes of researchers, patients and the general public. Broadly defined, HCAs are those who provide supportive services and personal assistance to disabled, elderly and/or ill (acute or chronic) individuals requiring either short-term aide or long-term support [1]. See Fig. 1 for a list of alternate HCA titles. HCAs are the primary care providers for frail and vulnerable older persons, who reside either in long-term care (LTC) or in their homes with home-based supports. In 2013, 14.1 % [2] of Americans and 14.9 % [3] of Canadians were over the age of 65; this segment of the population is predicted to increase by more than 200 % between 2012 and 2060 [4]. By 2050, ten percent of the populations of Organisation for Economic and Co-Operative Development (OECD) countries will be over the age of 79 [5]. In the European Union, near 25 % of the population are predicted to be 65 years or older by 2030; an increase of 8 % in only 25 years. A similar picture is evident across the globe [4]. Between 2010 and 2050, needs for care among older adults are predicted to nearly triple, with the most dramatic increases seen in low and middle-income countries [5]. These demographic trends will increasingly challenge the healthcare system as older people require different, and often more, health services than do younger people [6]. Chronic conditions, in particular, are strongly associated with age. Cognitive impairment and dementia, whose prevalence double with every five-year incremental age increase, are the leading global chronic disease contributors to older persons’ disability and dependence [5].

A number of global trends have contributed to reducing the likelihood that older adults will receive care from family members, including declining birth rates [4, 5], enhanced workforce mobility and urbanization [5], increased prevalence of single-parent households [4], a more highly educated female population [5], high divorce rates [4], and the tendency of adult children to live away from their families [4, 5]. As a result, demand for institutional and paid provision of care is high and will continue to grow [4]. For example, the need for LTC in Canada is expected to increase 10-fold by the year 2038 [7]. According to a U.S.-based report, those who reach age 65 have a 40 % chance of eventually entering a nursing home (NH); near 10 % of those who do will stay for five years or more. Notwithstanding, in OECD countries, the proportion of elderly receiving care within their homes is estimated to be as high as 65 % [4]. Societally, we are relying on a rapidly growing HCA workforce to provide quality care to our loved ones [5]. Thus, it is increasingly important that health care systems collect and use HCA data in workforce planning.

A better societal understanding of the HCA workforce is imperative, as future demand will be high and the existing supply of these workers is poorly understood. HCA positions are proliferating globally, across all healthcare settings. In the U.S., the HCA workforce has been profiled in several national surveys, the most recent in 2010 [8]: the majority are female, over 40 years of age, were born in the U.S., earn less than half of the U.S. national median annual earnings and have less than or equal to a high school education. According to UK estimates, 1.6 million are currently employed as front-line care providers in the social care sector, a value expected to double within the next 20 years [5]. The absence of national or provincial directories/registries in Canada means that less is known about the Canadian HCA workforce. Narrowly-focused systematic reviews of the literature relating to the HCA workforce have been published in the areas of workplace violence [9] and models of care [10]. Reviews on job satisfaction and burnout are forthcoming. None have taken a broad view of the workforce for purposes of workforce planning – exploring its characteristics, status, and future – as we have sought to do. The research questions guiding this review were:

1. What is the breadth and depth of the HCA workforce literature? Are there notable knowledge gaps?
2. What does existing literature tell us about the education, supply and use of HCAs, the demand for HCAs, and injury and illness among HCAs?

Specific areas of focus identified in Question 2 were selected based on Birch et al. [11] human resources planning framework (described in greater detail under Screening – Inclusion and Exclusion).

## Methods

We determined that a scoping review [12, 13] would best answer our guiding questions. Our questions were broad, and relevant studies that would aide us in answering these questions were diverse in both design and quality. Results of this study can inform the development of future systematic reviews of the literature, which address specific, relevant questions [12] relating to the HCA workforce. Scoping reviews, increasingly favoured in the field of health research, are conducted with a goal of “summarizing a range of evidence in order to convey the breadth and depth of a field” p. 1 [13]. The primary methodological difference between systematic reviews and scoping reviews are: 1) the specificity of the research questions guiding the review and 2) the incorporation of quality assessments into the review process [12]. Quality assessments are not typically conducted as part of a scoping review [12, 13]. We adhered to all PRISMA guidelines that applied to scoping review methodology. Our completed PRISMA checklist is included as Additional file 1.

### Search strategy

**Fig. 2:**
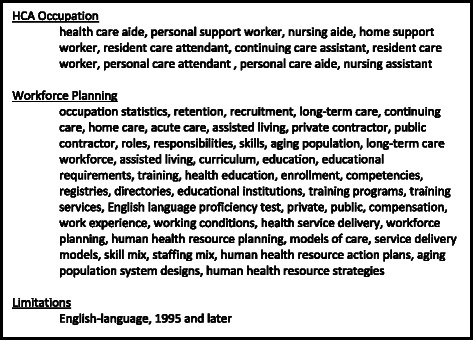
Key words used to search Medline

The search strategy included seven online bibliographic databases, which index journals from around the globe: MEDLINE® In-Process and Other Non-Indexed Citations, Ovid MEDLINE ® Daily and Ovid MEDLINE ® 1946-Present, EMBASE (1988-); PsycINFO (1987-); EBM Reviews– Cochrane Database of Systematic Reviews (2005-), Database of Abstracts of Reviews of Effects, Cochrane Central Register of Controlled Trials, CMR, Health Technology Assessment; CINAHL, Business Source Complete, ABI Inform. A health services librarian conducted the search on March 16, 2013. See Fig. 2 for a list of key words used in the MEDLINE® search or Additional file 2 for more detailed information on the MEDLINE® search.

### Screening – inclusion and exclusion

A single author screened each citation: the titles and abstracts were reviewed using the following inclusion and exclusion criteria. Inclusion criteria: 1) HCA or an equivalent position title, 2) Published in a peer-reviewed journal, 3) Published in 1995 or later, 4) Abstracts published in English. Exclusion criteria included: 1) Primary focus of the study was workplace violence, models of care, stress, job satisfaction or burnout as reviews on those topics have either been recently completed [9, 10] or are forthcoming, 2) Primary focus of the study was continuing education (as opposed to initial training) or quality of care (excluded due to challenges associated with isolating contributions of HCAs to quality of care in multi-disciplinary, heterogeneous settings), 3) Workforces not considered equivalent to HCAs such as family caregivers, unpaid caregivers or assistants to an allied health professional. If a study reported on a dependent variable that would otherwise qualify it for exclusion (i.e. workplace violence, models of care, stress, job satisfaction, burnout, continuing education or quality of care) but also reported on a dependent variable that met inclusion criteria (e.g. turnover), then it was included and only those results related to the “included” dependent variable were extracted and synthesized. In some cases, variables such as workplace violence or job satisfaction were independent variables predicting outcomes meeting inclusion criteria; in these cases, results relating to such variables were extracted and synthesized (e.g. results relating to the relationship between job satisfaction and turnover). As a means of “calibration”, the research team reviewed 20 titles and abstracts together before continuing with independent screening. If the reviewer of the title and abstract was at all unclear regarding the study’s eligibility for inclusion, the citation was brought forward to the manuscript screening stage.

We decided, *a priori*, to build on Birch et al. [11] human resources planning framework, a conceptual model that identifies constructs influencing the demand for, and supply of, health human resources, by categorizing included studies (topically) into the following groups: education, supply, use, demand, injury and illness. For the category of preparatory education, we anticipated finding studies reporting on student demographics, entrance requirements, curriculum, program length, certification (process and mandates) and regulation. Studies that examined the supply of HCAs were expected to focus on the demographic characteristics of existing HCAs - including immigration status, employment characteristics (such as wage), demographics of students entering vocational programs, recruitment into training programs and workforce shortages. The role of the HCA (level of autonomy and description of tasks), skill-mix, power differentials among staff, competencies and relevant position statements were expected to be retrieved within the category of use. We anticipated including studies that reported on various indicators of demand such as tenure, turnover and intent to leave (either/both the profession/facility) and factors, community-, facility-, or individual- level, associated with each indicator. This category also included studies relating to projected growth of the profession, recruitment and provision of benefits. In the category of injury and illness, we expected to find state, provincial or national profiles of HCA injury, absenteeism and illness rates.

### Data extraction

An author (either SH, SC, TS, HC or SR) extracted data elements, including author (year), journal, country, sample, setting, intervention (where applicable), data collection method, instrument(s) and measure(s), reliability and validity of instruments, data analysis and results relevant to any one of the categories of findings described above from each publication that met criteria for inclusion. HH inspected the contents of synthesis tracking tables through random verification of data elements.

### Analysis

Two to five authors, in a number of in-person meetings over a period of two months, collated and summarized study results [12], as captured through data extraction. Authors (SH, SC, TS, HC, SR, HH) compiled and organized results into category-specific tables (i.e. education, supply, use, demand, injury and illness). Through the review of these synthesis tracking tables, in which commonly reported results were grouped together (e.g. mean age of HCA participants), the research team was able to “apply meaning to the results” [13] and to detect notable gaps in the literature.

## Results

### Search results

**Fig. 3:**
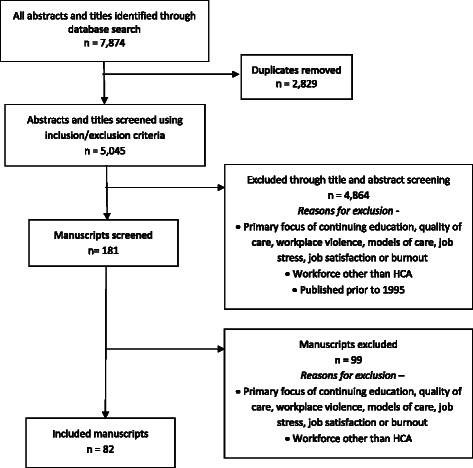
Flow diagram

We retrieved a total of 7,874 citations and removed 2,829 duplicates, which left 5,045 citations for screening. Of the 181 studies retained for manuscript screening, 82 were included (see Fig. 3). None were excluded based on language of publication. Characteristics of included studies (listed in alphabetic order) are available as a supplementary document (Additional file 3).

Most studies (*n* = 71, 87 %) were quantitatively designed and the majority utilized some form of regression analysis. Near one-fifth of the studies provided only descriptive statistics [14–30] and the remainder of those with quantitative results utilized simpler, bivariate statistical analyses [31–39]. Less than 10 % of included studies were qualitatively designed [18, 27, 40, 41]. Six [42–47] narrative, non-systematic literature reviews were included and a single methodological paper [48]. The majority of included studies were conducted in the U.S.; thirteen studies [21, 24, 26, 33, 35, 49–56] used data from the National Nursing Assistant Survey (NNAS) (most frequently from the 2004 data set) and six [35, 49, 56–59] used data from the National Nursing Home Survey (all from the 2004 data set). Other countries represented in the included studies were Australia [15], Brazil [19], Canada [27, 41, 44, 48, 60–64], Denmark [36, 37, 65, 66], Ireland [20, 29], Japan [67–69], New Zealand [28], Norway [70], Taiwan [32] and the United Kingdom [18, 42, 47]. See Table 1 for a categorical summary of synthesized results. The unabridged synthesis tracking tables are available as a supplementary file (Additional file 4).

**Table 1:** Summary Table

Education [14, 15, 18, 19, 21, 25, 26, 28, 29, 33–36, 40, 42–45, 47, 52, 53, 56, 69, 71, 72]	
Reasons for becoming an HCA	1) Desire to help or inclination to work with people
	2) Aspiration to work in healthcare
	3) Job security or desirable job benefits [33, 41, 53]
Transitions into HCA career (range)	Not working or unemployed: 22.1 % [53] to 28.4 % [72]
Perceptions of training (range)	Felt well-prepared for work by initial training: 38 % [42] to 96.5 % [33]
Initial training topics	*Patient Care:* Personal/resident care [21, 28, 33], lifting/handling, fall prevention, medications, nutrition, First Aid, continence, oral hygiene [26], talking with residents [21, 33]. Perceived as excellent by 57.9 % to 66.6 % of HCAs [21]
	*Holistic Care*: Recognizing abuse, philosophy and values, cultural safety, sexuality, common disabilities [28], dementia care, discuss resident care with family, work with abusive residents [21, 33]. Perceived as excellent by 41.9 % to 44.6 % of HCAs [21]
	*Provider and policy*: Personal safety, emergency procedures, infection control, service policy/protocol, risk management, fire and safety [26], record resident information, prevent work injuries, organize tasks, work with supervisors, work with coworkers, problems-solve work issues [21, 33]. Perceived as excellent by 32.2 % to 52.8 % of HCAs [21]
Requested topics for initial training	*Patient care*: Care skills [21, 29], talk with residents, medication management, pain management [21]
	*Holistic care*: Abusive residents [21, 29], discuss resident care with family members, work with residents family, dementia care [21], mental health training, integrating health promotion, multicultural training, challenging behavior skills [29]
	*Provider and policy*: Work with coworkers, organize work tasks, work with supervisors, problem solving for work issues, record resident information, prevent work injuries [21], physical preparation for the role, training in management, stress management [29]
Location of initial or vocational training (range)	Facility employing: 43.1 %, among immigrants [33] to 65.3 %, rural [53]
	Community college 15.7 %, among immigrants [33] to 23.8 %, micropolitan [53]
	High school: 6.0 %, micropolitan setting to 6.3 %, rural [53]
	Vocational or trade school: 5.4 %, micropolitan to 6.6 %, urban [53]
Cost of training (range)	Entirely paid for by employer: 67.9 %, urban [53] to 78 % [28]
Training hours (range)	Ratio classroom to clinical: 50:50 [69] to 95:5, in “other” long-term care settings [14]
Qualification	National Vocational Qualification (UK): 4 levels of qualification [18, 42, 47]
	Home Helper (Japan): 3 levels of qualification [69]
Supply [14–22, 24, 27, 28, 32–35, 39–41, 44, 45, 48–58, 62, 66–68, 70, 72–85]	
Mean age (range)	Exact ages - 36 [78] to 47.6 [32]; Age ranges – 31–35 [55] to 41–50 [75]
Education (range)	High school or less: 40.4 %, home health [41] to 92 %, nursing homes [80]
	Some college/post-secondary: 8 %, home health, nursing home and assisted living [79] to 38.7 %, hospital [17]
Marital status (range)	Married/living with partner: 38 %, hospital [22] to 82.6 %, Danish [66]
Dependents (range)	Adult or child, living at home (U.S. specific): 38.9 %, non-immigrant [33] to 60.5 %, female [52]
	Children under 18: 28.8 %, home health HCAs [81] to 52 % [76]
Primary language (range)	English: 74.5 %, home health [17] to 99.1 %, rural [53]
	Non-English: 1 %, non-immigrant to 50.9 %, immigrant [33]
Immigration status (range)	U.S. citizen: 88 % [76] to 99.3 %, rural [53]
	Non-U.S. citizens: 6 % female to 17.3 %, male [52]
Gender (range)	Female: 76 %, permanent full-time (Japan) [67] to 98.3 % [66]
Employment characteristics (range)	Full-time: 14 %, Canada [48] to 79.3 %, hospital [81]
	Weeks worked annually (mean): 40.7, home health [17] to 47.5, hospital [81]
	Weekly hours worked (mean): 13, home care (Danish) [28] to 38, U.S.-based nursing home [22]
	Weekly overtime hours worked (mean): U.S.-specific 9.71 [57] to 10.1 [58]
Shift work (range)	Mainly day: 43.4 % [66] to 61 %, nursing home [80]
	Mainly evening: 22 %, nursing home [80] to 24.8 % [66]
	Mainly night: 10.5 % [66] to 17 %, nursing home [80]
Wage (range)	Hourly in U.S. dollars (mean): $7.45 in home health and nursing homes, 2002 [39] to $17.84 in home health, 2006 [22]
	Household income < $30,000: 49.6 %, home health [35] to 70.3 %, female [52]
	<150 % federal poverty level: 18 % [84] to 37.9 %, home health [39]
Requiring federal assistance (range)	Any: 5 % [76] to 31.4 %, nursing home [33]
	Food stamps: 10.78 %, nursing home [24] to 14 % [84]
Use [18, 29, 30, 40, 42, 45–47, 57, 70, 80, 81, 85–91]	
Tasks assigned	Patient contact: provide personal care [18, 42] (indirect and direct) [40, 45], feeding [40, 45, 47], oral care [40, 45]
	Physical [40, 70]
	Clerical/Administrative [40]: general [42, 45, 47]
	Non-patient contact [42]: housekeeping [40, 47]
	Similar to RN [29]: Administer medications, catheterization [42, 47]
Staffing (FTE/100 residents)	HCA: 25.3 [80] to 38.5 [89]
	RN: 8.5 [80] to 25.9 [89]
	LPN: 11.2 [80] to 23.7 [89]
Demand [15, 16, 22, 24, 27, 28, 31–35, 39–42, 44, 47–50, 53–59, 62, 65–69, 72–77, 79, 80, 82–89, 91–94]	
Projected growth of the profession	HCAs: 62.5 % (2000–2010) [84] to 114 % (2010–2020) [48]
	Home health aides: 47.3 % (2000–2010) [84] to 69.4 % (2010–2020) [74]
Tenure in profession (range)	Months (mean): 79.2 [68], nursing home to 148.8 [80]
	11-20 years: 22.3 % [24] to 22.8 % [53], both in nursing homes
	>20 years: 12.3 % [35] to 12.5 %, nursing home [53]
Turnover – profession	Within 2 to 3 years of training: 37 %, Denmark [65] to 46.3 %, Taiwan [32]
Tenure in facility (range)	Months (mean): 25.96 [56] to 118.3 [79], both in nursing homes
	<2 years: 41.8 %, rural to 42.6 %, micropolitan [53]
Turnover – job/facility (range)	Annual: 59.4 % [91] to 170.5 % [86]
	6-month: 13.1 % [75] to 64.4 % [88]
	3-month: 18.8 % [57] to 19 % [58]
Community and facility-level factors related with turnover (—, + or NS)	*Community:* High unemployment rate: (—) [57, 82, 91]
	*Facility*: For-profit status: (+) [58, 82, 83, 87, 94]
	Chain membership: (+) [93, 94],(—) [80], NS [82]
	Higher LPN staffing levels: (—) [80, 91]
	Greater HCA HPRD: (—) [57, 58, 86]
	High HCA wages: (—) [28, 58, 72, 85, 87, 94]
	Provision of benefits: (—) [58, 72], NS [22, 82, 93]
	Union contract in place: (—) [58, 92]
	Greater HCA perceived quality of care: (—) [80, 88, 89]
Impact of interventions on turnover	0.2 FTE Retention Specialist x 6 months: (—) (*p* < 0.05) [75]
	Multi-pronged curriculum based intervention: (—) (*p* ≤ 0.05) [83]
Intent to leave facility/job (range)	33.8 % (≥50 years) to 61.0 % [54]
Community and facility-level factors related with intent to leave facility/job (—, + or NS)	*Community:* Job alternative: (+) [79], number of nursing homes in county (—) [80]
	*Facility*: Rewarding income: (—) [32, 55, 68, 79, 80]
	Insurance coverage: (—) [49, 72]
	Supportive supervision: (—) [32, 49]
Recruitment into employment	Word of mouth [16, 33, 40]
Individual factors related with turnover (—,+ or NS)	Age: Increasing age (—) [22, 72, 94]
	Race/ethnicity: White (Reference), Hispanic (+), Black NS, Other NS [22], Hispanic NS [92], Racial minority (—) [80]
	Marital status: Married (+) [80], NS (compared to home health aides) [22]
Individual factors related with intent to leave (—, + or NS)	Age: Younger (+) [54, 67, 68]
	Shift: Nights (+) [67, 68]
	Education: > High school (+) [49, 79]
	Job security: High (—) [56], Low (+) [68]
	Job history: >2 jobs in last 5 years (+) [49, 79]
	Job satisfaction: High (—) [49, 80]
Benefits (range) – U.S. specific unless otherwise stated	Without health insurance: 12.7 %, immigrants employed in nursing homes [33] to 33 %, home health aides [82]
	Health insurance available: 83.3 % [49] to 91.6 %, micropolitan [53]
	Utilize/access health insurance: 25.5 %, home health to 62.3 %, hospital [39]
	Pension plan: 60 % [58] to 71.2 %, micropolitan [53]
	Paid sick time: 65.7 %, micropolitan [53] to 79.0 %, nursing home [58]
	Paid vacation days: 64 % [58] to 89 % [56], both in nursing homes
	Subsidized transportation: 3.9 %, rural [53] to 38.7 %, Canada [27]
Unionization	U.S. NHs: 10.4 % of HCAs [77] to19 % of facilities [92]
Illness and Injury [24, 44, 49–51, 60–64, 73, 90, 94]	
Work-related injury rate (range)	Proportion of HCAs injured: 18.5 %, home health aides [94] to 59.44 %, NHs [49]
	Number of injuries per HCA (average): 1.54 [51] to 2.63 [24]
Types of injuries	Most common: MSI [60, 64]
Rate of injury by profession	HCA higher than RN [61, 62, 64]
	HCA higher than LPN [61, 64]
Rate of injury by setting	Highest in LTC, as compared to acute care and community [60, 61]
Injury claim/sickness absence	HCA have higher rate than RN [61, 63]
	HCA and LPN have similar rates [60, 63]
Factors related to risk of injury (—, +, NS)	Availability of equipment: (—) [50, 64]
	Workplace aggression: (+) [44, 64]
	Lower age: (+) [58], (—) [62]
	Gender: Female (+) [60, 62]

### Education of HCAs

Twenty-five studies were relevant to HCA education [14, 15, 18, 19, 21, 25, 26, 28, 29, 33–36, 40, 42–45, 47, 52, 53, 56, 69, 71, 72]. Common reasons for becoming an HCA, ranked by North American HCAs in order of importance, were: 1) a desire to help [33, 53] or an inclination to work with people [41], 2) an aspiration to work in health care [33, 53], and 3) job security [33, 53] or related, desirable job benefits [41]. Transitions into HCA professions were explored in two U.S.-based studies [53, 72]: one [72] reported that 28.8 % of those newly entering the HCA workforce had most recently been unemployed (unrelated to disability).

The majority of U.S.-based HCAs sampled felt well prepared for work by their initial training [21, 34, 42, 35, 52, 56]. Three categories of initial training topics offered in HCA preparatory programs emerged from the included studies: patient care, provider and policy and holistic care. The majority of U.S. HCAs rated their initial training (by topic) as excellent [21, 33]. Among the clinical topics provided in initial training in the U.S., *Working with abusive patients* and *Dementia care* were least often rated as excellent [21, 33]. Requested topics for initial training among U.S.-based HCAs aligned with topics least often identified as excellent in preparatory programs, such as *Dementia care*, *Dealing with abusive residents*, *Problem solving* and *Working with co-workers and supervisors* [21, 29].

In North America, the trend is for HCAs to obtain their initial training at the college level [26, 44] rather than by attending facility-provided or on-the-job training; in the U.S., 15.7 % of immigrant HCAs [33] to 23.8 % of HCAs (micropolitan) [53] completed initial training at a community college. Approximately half of current U.S.-based HCAs had received their training in the facility where they were employed [21, 33, 35, 53] and close to 6 % were trained in a high school setting [35, 53]. All included studies that discuss cost of training (and who pays for it) were published in the last five years, reflecting a recent interest in the subject [21, 26, 28, 53]. In the U.S., responsibility for cost of training appeared to correspond with location of training, in that the cost of facility-provided training was more likely to be covered by the employer [21, 53]. Time spent in the classroom (as opposed to a clinical setting), expressed as a proportion of initial training time, ranged from 50 % for entry-level home health workers in Japan [69] to 95 % for U.S.-based HCAs employed in “other” LTC settings (including community-based services and home health) [14].

Globally, regulation of the HCA workforce is inconsistent, as is licensure of individual HCAs, whether optional or mandatory. Two countries – the UK, with National Vocational Qualifications [18, 42, 47] and Japan, with Home Helper certifications [69], have instituted skill-based classification systems for HCAs.

A single study [19] from Brazil provided data on student demographics. No included studies reported on entrance requirements for vocational programs. Although some studies [14, 21, 35, 69, 72] provided information on the number of training hours required to complete HCA programs, none provided details on the length or structure (i.e. part-time vs. full-time, day vs. night) of programs.

### Supply of HCAs

Fifty-one included studies reported on the supply of HCAs [14–22, 24, 27, 28, 32–35, 39–41, 44, 45, 48–58, 62, 66–68, 70, 72–85]. HCA mean age range was 36–45 years [14, 17, 22, 24, 27, 35, 39, 41, 49, 52, 68, 79, 81]. Some variation across settings was evident; U.S.-based home care settings [35, 79] had the highest mean age range, where it was 44 [41] to 46.7 years [81]. The majority of U.S. HCAs’ highest level of education was high school or less [21, 22, 33, 34, 49, 50, 54–56, 72, 76, 77, 80, 81]; this was particularly prevalent among NH and “native” born HCAs (*p* < 0.05) [33] (*p* < 0.001) [54]. Overall, approximately 25 % of U.S. HCAs have some college/post-secondary education [55, 75]. In the U.S. 38.0 % of hospital HCAs [22] to 52.0 % of nursing home HCAs [56] were reported as being married or living with a partner. In Denmark, 82.6 % were similarly coupled [66]. Across settings and studies, proportions of HCAs that were single, widowed, divorced, or married were fairly consistent.

In the U.S., between 38.9 % of non-immigrant HCAs [33] and 60.5 % of female HCAs [52] had a dependent living at home (adult or child). The range of U.S. HCAs with children under the age of 18 was 28.8 % in home health [81] to 52.0 % [76]. In the U.S., the range of those without children was 41.1 % among NH HCAs [24] to 66.5 % among home health aides [81]. Proportionally, in the U.S., 74.5 % of home health aides [17] to 99.1 % of rural HCAs [53] consider English to be their primary language. Two studies [15, 33] reported on language use on the job, of which one [33] addressed impact of the quality of English spoken on communication between HCAs and residents, and HCAs and other staff. Of HCAs working in the U.S., 88 % [76] to 99.3 % (rural) [53] were U.S. citizens; 6 % (female) to 17.3 % (male) [52] were not. The results of two studies [17, 39] indicate that the home care sector employed proportionally fewer U.S.-born HCAs than hospitals or NHs. The majority of HCAs were female, with a range of 76.0 % in permanent, full-time positions (Japan) [67] to 98.3 % [66]. No gender differences were apparent across work settings [17, 39].

In the U.S., the proportion of HCAs working full-time ranged from 62 % [76] to 79.3 % (hospital) [81]. A single Canadian study [48] reported that 14 % of HCAs were employed full-time. Far fewer U.S.-based HCAs in the home health care sector were employed full-time, compared to those in NHs or hospitals [17, 22, 39, 81]. In three U.S.-based studies [17, 39, 81], fewer full-time HCAs worked in NHs than hospitals. Mean weeks worked per year by HCAs in the U.S. ranged from 40.7 in home health [17] to 47.5 in hospital [81]. Mean hours worked per week ranged from 13 in the Danish home care sector [28] to 38 in U.S.-based NHs [22]. Many HCAs worked overtime (OT); average OT hours worked per week in the U.S. ranged from 9.71 [57] to 10.1 [58]. The majority of HCAs primarily worked day shifts, followed in frequency by evening then night shifts [66, 80].

Mean hourly wage (in USD) ranged from $7.45 in nursing home and home health settings (year - 2002) [39] to $17.84 in home health (year - 2006) [22]. Between 2001 and 2005, the most frequently reported hourly wage (in USD) was within a few cents of $10.30 [24, 49, 53, 72, 77]. The most recent (2012) reported hourly wage was $11.47 USD [40]. Hospital-employed HCAs in the U.S. had the highest hourly wage, compared to those in NHs and in the home care sector [17, 39, 81]. For the majority of U.S.-based HCAs, annual household income was less than or equal to $30,000 USD [24, 33, 35, 51, 52]. The overall proportion of U.S. HCAs earning less than or equal to 1.5 times the U.S. federal poverty amount ranged from 18 % (across all settings) [84] to 37.9 % (among home health aides) [39]. Home health HCAs in the U.S. were more likely to be in poverty than HCAs employed in a NH or hospital [17, 39]. Five percent [76] to 31.4 % [33] of U.S. HCAs were reported as receiving public assistance, with 10.78 % [24] to 14 % [84] utilizing food stamps. Three studies [44, 67, 72] reported HCAs working multiple jobs; no trends were noted.

None of the included studies provided information on the demographics of students entering vocational programs, methods of recruiting students into training programs or quantified existing workforce shortages or surpluses.

### Use of HCAs

Nineteen included studies reported on use of HCAs [18, 29, 30, 40, 42, 45–47, 57, 70, 80, 81, 85–91]. HCAs were employed in a wide variety of care settings with a diverse group of patients. A single message appeared most clearly and consistently in the literature addressing use of HCAs: the role of HCAs was unclear [29, 42, 45–47] – heterogeneity in duties, level of autonomy, setting of work and population makes describing “the” role of a HCA near impossible. Tasks assigned to HCAs reflected five categories: physical tasks, patient contact tasks, non-patient contact tasks, clerical tasks/administrative and tasks similar to Registered Nurses (RNs). Patient contact tasks, such as bathing and feeding, were most frequently reported [18, 40, 42, 45, 47]. However, responsibilities ranged from oral care [40, 45] and shaving [45] to venipuncture [42] and catheterization [42, 47].

Despite ongoing interest in enhancing skill-mix in health care organizations, we found only one empirical study [30] related to HCAs and skill-mix. The U.S.-based study explored shifts in staffing levels of RNs, Licensed Practical Nurses (LPNs) and HCAs in NHs between 1997 and 2007. RN staffing, calculated in hours per resident day (HPRD), declined while LPN and HCA HPRD increased. Such shifts in skill-mix were most pronounced in the for-profit sector [30].

No position statements were retrieved that met our inclusion and exclusion criteria.

### Demand for HCAs

Fifty-three included studies reported results related to demand for HCAs [15, 16, 22, 24, 27, 28, 31–35, 39–42, 44, 47–50, 53–59, 62, 65–69, 72–77, 79, 80, 82–89, 91–94].

#### Projected growth

Four North American studies [48, 72, 74, 84] offered estimations of projected growth in the HCA workforce. Periods during which growth was estimated varied; however, it is clear that growth is projected to be both sizeable and rapid. Two studies [74, 84] counted projected growth in a number of HCA-related job categories (Home Health Aide, Personal and Health Care Aide, Nurse Aide/Orderly) as among the fastest growing job categories in the U.S.

#### Working tenure

The range for mean months of work as an HCA was 79.2 (in NHs) [68] to 148.8 [80]. The proportion of HCAs with working tenure of less than five years varied across countries. After five years, proportions become more consistent [24, 32, 35, 50, 53, 54, 62, 67]. The proportion of HCAs in the U.S. having worked 11 to 20 years was 22.3 % [24] to 22.8 % [53]. In the U.S., near 12 % [24, 35, 53] had worked more than twenty years as a HCA.

Two international studies [32, 65] reported a sample-specific proportion of qualified HCAs leaving the occupation within two to three years of training (37 % [65] and 46.3 % [32]). Two studies [55, 68] reported intent to leave the occupation; results could not be synthesized due to variation in measurement (percentage intending to leave [55] versus average intention to leave on an ordinal scale [68]).

#### Facility-level findings

The mean range, in months, for tenure in a facility was 25.96 [56] to 118.3 [79], with variability within and across settings. Two studies using the U.S.-based NNAS database reported that approximately 40 % of HCAs were in their facilities for less than two years [24, 53].

Turnover rates were reported differently across studies: annually, semi-annually and quarterly. Annual turnover rates ranged from 59.4 % [91] to 170.5 % (in NHs) [86]. Six-month turnover rates, all reported in U.S.-based studies, ranged from 13.1 % [75] to 64.4 % [88]. Two U.S. studies [57, 58] reported three-month turnover rates near 19 %.

##### Community characteristics

High unemployment rates in the community were correlated with reduced turnover in three [57, 82, 91] of five U.S.-based studies [83, 92]. The remaining community-level factors associated with turnover were reported in single studies.

##### Facility characteristics

Several facility-level characteristics were associated with turnover of HCAs. For-profit status was positively associated with increased turnover in the U.S. [58, 82, 83, 87, 94]. Within the U.S., the association of chain membership with turnover was equivocal [80, 82, 93, 94]. Two U.S. studies [80, 91] found that higher LPN staffing levels reduced turnover among HCAs. Three others [57, 58, 86], also U.S.-based, found that greater HCA hours per patient day (HPPD) reduced turnover rates.

Higher HCA wages were negatively associated with turnover [28, 58, 72, 85, 87, 94]. The relationship between benefit provision and HCA turnover in the U.S. was inconclusive, with two studies [58, 72] reporting a negative association with turnover and three [22, 82, 93] reporting non-significant effects. Two U.S.-based studies [58, 92] reported that having a union contract significantly reduced turnover.

Three administrative factors significantly reduced turnover among U.S. HCAs: management seeking input [91], flatter management structure [92] and having a supervisor trained in management [92]. Higher quality of care, as perceived by HCAs, was related to lower HCA turnover in U.S.-based studies [80, 88, 89].

##### Interventions to address HCA turnover

Two intervention studies [75, 83], conducted in the U.S., reported a small but significant reduction in turnover associated with the intervention; either the addition of a 0.2 full-time equivalent (FTE) Retention Specialist for six months (*p* < 0.05) [75] or a multi-pronged curriculum-based intervention (*p* ≤ 0.05) [83].

##### Intent to leave

Four U.S.-based studies [49, 54, 55, 79] measured intent to leave among HCAs with proportions of up to 61% (non-U.S. citizens working in the U.S.) being very likely or somewhat likely to leave their job in the next year [54]. The relationship between available job or employment alternatives in the community and intent to leave was reported in two U.S. studies [79, 80]; results were equivocal. Satisfaction with compensation [32, 55, 68, 79, 80], provision of insurance coverage or benefits (U.S.-specific) [49, 72] and supportive supervision [32, 49] were all significantly negatively associated with intent to leave among HCAs.

#### Recruitment

In the U.S., word of mouth was more frequently cited [16, 33, 40] than formal recruitment methods, such as advertisements and announcements within schools and training programs, as a method of finding employment.

#### Individual characteristics

Two [22, 94] of three [80] studies, conducted in the U.S., reported that increasing age was significantly associated with lower individual risk of turnover. The relationship between race/ ethnicity and turnover among U.S. HCAs was statistically significant [22, 80, 94], but equivocal. The relationship between marital status and turnover among U.S. HCAs was inconclusive [22, 80].

##### Intent to leave

Younger age was positively associated with intent to leave [54, 67, 68, 72]. Results related to intent to leave by gender, reported in two studies based in Japan, were equivocal [67, 68]. In Japan, HCAs who worked the night shift reported greater intentions to leave their job [67, 68]. In the U.S., education levels greater than high school were associated with an increased likelihood of intending to leave a job [49, 79]. Job security was reported to reduce the likelihood of intent to leave [56, 68]. Individuals with more than two jobs in the past five years had greater intent to leave than those who had two or fewer [49, 79]. U.S. HCAs with high job satisfaction had lower intentions to leave [49, 80].

#### Benefits

The proportion of U.S. HCAs without health insurance ranged from 12.7 % among immigrants employed in NHs [33] to 33 % in home health [82]. Of the population of HCAs employed in U.S. NHs, 83.3 % [49] to 91.6 %, in a micropolitan setting [53] had health insurance available to them. Despite this, only 25.5 % (in home health) to 62.3 % (in hospital) [39] opted to utilize employer-provided health insurance. In the U.S., payment of health insurance varied significantly in that employers paid either all insurance fees for employees and their family, all fees for employees only, partial fees for employees and their family, or partial fees for the employee only [56, 58, 59]. The proportion of U.S. HCAs with a pension plan ranged from 60 % [58] to 71.2 % in micropolitan NHs [53]. Similarly, the proportion of U.S. HCAs with paid sick time varied from 65.7 % in micropolitan NHs [53] to 79 % in NHs [58]. The proportion of U.S. HCAs with access to paid vacation/personal days ranged from 64 % [58] to 89 %, both in NHs [56]. The proportion of Canadian home health aides receiving any of the above benefits was lower than the lowest reported U.S. rates [27, 44]. Provision of subsidized transportation varied across settings with 3.9 % [53] of rural NH HCAs receiving it and 38.7 % in home health care (Canada) [27]. Union membership differed across countries: in the U.S., proportions ranged from 10.4 % [77] to 19 % in NHs [92]. In a Canadian study [41], 38 % of home HCAs were union members. Home HCAs, whose wages were generally lower, were also less likely to receive benefits [44, 59].

### Injury and Illness of HCAs

Thirteen included studies reported on injury and illness in the HCA workforce [24, 44, 49–51, 60–64, 73, 90, 94]. Most reported on individual and unit-level factors related to workplace injury – as opposed to environmental or institutional factors. Few studies provided overall injury rates; more commonly, rates were reported as a function of another variable, such as type of injury [51, 60], job status [62], or as a unit-specific rate [60, 61, 63, 64] (e.g. per 100 FTE or per 100 person-years). In the U.S., the overall injury rate among NH HCAs was 59 % [24, 49]. A significantly lower annual injury rate of 18.5 % was reported among U.S.-based home health aides [94].

The most common types of injuries experienced by HCAs in Canada were musculoskeletal injuries [60, 64], which, in one study [64] made up 84 % of all injuries. Other injuries described in the literature included puncture [60], irritation and allergy [60], psychological trauma [60], scratches/cuts [51, 60], human bites [51] and bruises [51, 60].

Across all healthcare settings in Canada, HCAs had a higher injury rate than both LPNs and RNs [61, 62, 64]. The highest HCA injury rates were reported in NHs, as compared to in community and acute care settings [60, 61]. In one study conducted in the Canadian NH setting, HCA injury rates (37.0 per 100 FTE) were 2.15 times greater than those of RNs (17.2 per 100 FTE) [60].

Although injury claims and sickness absences among HCAs in Canada outnumbered those of RNs [61, 63], the associated annualized costs were greater for RNs [63]; this may have been due to differences in RN and HCA compensation. In two Canadian studies [61, 63], HCA and LPN sickness rates and number of days lost due to falls were more similar than those of LPNs and RNs. Also in Canada, average sick days per-person year productive hours [63] and median days lost [61] were greater among HCAs and LPNs than among RNs. Absenteeism, in the form of sickness absence [63] and absence due to injury [24] was addressed in two studies with equivocal results.

In the U.S., HCAs with less training (*p* < 0.05) [50], less HCA experience (*p* < 0.05) [50] or who reported feeling less prepared by their training for work (*p* < 0.05) [94] were more likely to be injured on the job. Availability of equipment was related to reduced rate of injury (*p* < 0.01 [50]) [50, 64]. Workplace aggression was reported as a risk factor for injury [44, 64].

The relationship between HCA age and injury and illness rates, in Canadian settings, was equivocal [60, 62]. Gender differences were apparent in Canadian settings, with the evidence suggesting that injury rates were higher among females [60, 62]. The relationship between injury rate and gender differed by job status - female HCAs working full-time hours had higher injury rates than those working casual hours (*p* < 0.05), while injury rates among male HCAs working part-time hours were higher than their full-time counterparts (*p* < 0.05) [62].

## Discussion

The HCA workforce is both invisible and ubiquitous; as long as this is the case, governments and healthcare organizations will be limited in their ability to develop and implement feasible, effective workforce plans for HCAs. The continued undervaluation of HCAs adversely impacts care providers, the institutions they work for and those who depend on their care [5].

Globally, health systems are called upon to do more with less – less space, less staff, less money. In high-income countries, current LTC costs and funding models have been characterized as unsustainable. The costs associated with LTC in high income countries, as a proportion of global domestic product, are expected to double within the next 5 years. Global aging is acknowledged by Standard and Poor’s, a credit rating agency, as a notable threat to the stability of the economy. Low and middle income countries are also affected; in these countries, where care has traditionally been provided by family members there will likely be a discernible shift from informal to formal care services [5]. Policy-makers need to think strategically and to proactively initiate strategies targeted at measuring the existing workforce, optimizing HCA training, attracting competent candidates to training programs and improving the working conditions for this workforce.

The results of this review make clear the degree to which HCAs, in general, are marginalized. Improvements to work conditions, respect and acknowledgement are hard won; HCAs cannot rely on others, be they professional groups or public-interest groups, to dedicate the time and energy required to effect change. Unfortunately, they face many challenges in their bid to achieve equitable treatment and recognition. Savage et al. [95], in their UK-based social class survey analysis, identified HCAs as one of the ‘over-represented’ occupations within the sizeable precariat class, which also includes cleaners and retail cashiers. The precariat are the poorest and most deprived of the seven identified classes - lacking in social, cultural and economic capital [95]. Poverty is associated with self-efficacy - that is, an individual’s judgement about their own ability to coordinate and carry out what is necessary to achieve a desired outcome; those with low self-efficacy approach career management with less maturity than those with high self-efficacy [96]. Interventions, either in the workplace or in initial training programs, targeted at improving self-efficacy within the HCA workforce, such as the introduction of a program offering vocational assistance [96], may better prepare HCAs to advocate for their occupation and to work productively alongside existing workforce advocates. Effective advocacy takes time, resources, experience, connections and confidence – luxuries that few working poor have to draw upon. Poverty has been shown to profoundly influence the behaviours, perceptions and relationships of individuals [96]. Strong unions, associations or particularly influential “champions” are often needed to initiate and carry through these changes. Champions like Leonila Vega, a Mexico-born lawyer, and the former executive director of the Direct Care Alliance, an organization that focuses on fostering a grassroots movement among HCA leaders [97], are needed across the globe. This organization, along with others, played a key role in pushing the U.S. federal government to extend the Fair Labor Standards Act to protect the rights of domestic service workers (including privately employed home health aides). The new regulations were finalized in September 2013 and will be in effect January, 2015 [98]. Until HCAs are in a position to effectively advocate for themselves or are effectively advocated for around the globe, it behooves health care administrators and policy makers to develop policy and legislation that protects this workforce, and indirectly, the vulnerable older persons who rely on their care.

Our results clearly demonstrate a hierarchy within the HCA workforce, home health aides have the least job stability, lowest pay, fewest work hours and are the least likely to have fringe benefits, as compared to HCAs employed in hospitals and NHs. This seems counter-intuitive, as home health aides work autonomously and rely on a more comprehensive knowledge and skill base than their facility-employed counterparts. Additionally, due to the informal nature of many home health care employment arrangements, existing workforce data does not truly reflect their numbers or working conditions. Evidence suggests that HCAs can be hired on the grey market at an appreciable ‘discount’ [5]. Proportionately, more home health aides are immigrants – migrant workers are more likely to be paid under the table, reducing the likelihood of labour law enforcement [99]. Migrant HCAs, as employees in their client’s home, may encounter such problems as poor living conditions, harassment, and limited personal time. Meanwhile, their home country’s increasing reliance on the money they send home [5, 100] prevents them from speaking out and risking deportation. The extension of the Fair Labor Standards Act in the U.S. is a step in the right direction. Internationally, policy makers should seek to better measure the demographics of the home health workforce and to legislate and enforce safeguards that protect all involved – employee, employer and the dependent client.

A clear knowledge gap exists in relation to the education of the HCA workforce. None of the included studies reported on program entrance requirements or provided information related to program structure and length. We were unable to make any conclusions about: which content is essential to a HCA program curriculum; which types of students are attracted to these programs; how much time is required for students to learn all that is needed or how much of that time should be spent in the classroom versus in a practice setting. The lack of clarity surrounding the education of HCAs is, undoubtedly, related to the lack of clarity regarding the HCA role(s). The delineation of HCA competencies and scope of practice is essential prior to moving forward with standardization of educational programs. A recent Alzheimer’s Disease International (ADI) report [5] echoed the recommendation of the Institute of Medicine’s Committee on the Future Health Care Workforce for Older Americans [101] that the minimum number of required training hours for HCAs be 120 (in all countries) and that HCAs be required to demonstrate competence in caring for older adults prior to certification; the current federal minimum in the U.S. is 75 training hours, an amount established in 1987 [5]. Additionally, ADI recommends that, for existing uncertified HCAs, competencies be identified and categorized as either core (required) or advanced (optional) [5]; these competencies could then be used to guide HCAs, training organizations and regulators in determining what types of training and education are needed to ensure a high quality of care.

Governing bodies that have jurisdiction over care provider regulation and educational institutions vary from country to country. Regardless of the country, multiple governmental departments need to be involved in the development of policy that impacts the HCA workforce (e.g. ministries of education, health, immigration). Policy makers, as civil servants, are duty-bound to collaborate with all relevant departments and ministries in order to develop and rally support for policies that optimize HCA education and employment and, in turn, optimize the safety and quality of care provided to those persons accessing the services of a HCA.

Many questions remain unanswered about the current and future state of the HCA workforce. We recommend future research, including targeted systematic reviews of the literature, in five areas:

1. Education: Optimal program content and delivery – What is the best location for training programs? Is there a benefit to offering setting-specific streams for training (e.g. hospital, NH/LTC, home health) or should all HCAs be trained to work in all settings? Internationally, how do existing programs compare?
2. Supply: None of the included studies described the students entering HCA training programs, methods used to attract students into programs or quantified existing surpluses/ shortages of HCAs. Are HCAs-to-be similar to existing HCAs? What is the attrition rate between training and employment? What made students decide to enter the training program? Are there international differences? Is there an existing shortage of HCAs? How has/would regulation and/or mandatory certification of HCAs impacted supply?
3. Use: Heterogeneity of role descriptions – What tasks and competencies are universally accepted as appropriate for HCAs? What are the motivations of health administrators asking HCAs to complete tasks not traditionally completed by HCAs? What is the optimal setting-specific skill-mix?
4. Demand: Turnover and continuity of care – Are the predictors of turnover among HCAs similar or different to the predictors of turnover among other health professionals or among similarly-educated employees in other sectors? Would the introduction of career laddering opportunities enhance job satisfaction and reduce turnover?
5. Injury and illness: Safe practice, absenteeism and continuity of care – Does regulation of the workforce by policy makers ensure safe and quality practice? What is the average rate of absence among HCAs? How does it compare across professions (both in frequency and in reason for absence)? Does absenteeism related to injury and illness in this workforce negatively impact client and resident outcomes? What are employers currently doing to reduce rates of injury and illness among HCAs?

Future research on the HCA workforce will depend on repeated, institutionalized collection of demographic information. In the absence of regulation, at minimum, inclusive HCA registries should be instituted in all countries. It takes time to successfully plan and implement a nation-wide registry. Assuming countries heed our advice and begin the process of instituting a nation-wide, inclusive registry of HCAs, it will be a minimum of three years before workforce planners and researchers will have reliable access to registry data. Many countries have publicized projected future care needs [102] but few have the data required to evaluate the gap between the future need (as projected) and the current supply and/or existing training capacity.

### Limitations

We conducted a scoping review of the HCA workforce literature. We are confident in the rigour of our methodology but recognize that independent review of all titles and abstracts by more than one author may have enhanced the reliability of our screening. We excluded articles published before 1995; it is possible that the inclusion of relevant articles published prior to 1995 may have added to our results. Use of validated tools to evaluate the quality of included studies may have allowed us to more definitively evaluate the reliability of the synthesized findings. However, as we were seeking to determine the breadth and depth of the HCA workforce literature, and thus planned to include all studies meeting inclusion criteria in synthesis, completion of quality assessments is unlikely to have significantly altered our results. Although we had, *a priori*, identified positions statements as sources of information on the HCA workforce (particularly in the category of Use), we excluded many of them by including only peer-reviewed literature; many position statements are never published in peer-reviewed journals. In the absence of formal quality assessments, following a thorough reading and review of all included studies, we would rate the overall quality of included papers as fair. This is in recognition of identified weaknesses in either or all of study design, methodology, and analysis.

## Conclusion

This scoping review offers policy makers a review of the breadth and depth of current knowledge of the HCA workforce. Categorization of results into education, supply, use, demand and injury and illness allowed for maximal clarity in synthesis and in the presentation of results. The results present a picture of a marginalized workforce charged with caring for a vulnerable segment of the global population. Home health aides face the poorest working conditions. Notable gaps in the literature were apparent, particularly in the areas of education and HCA use. Future research will require national HCA registries or, at minimum, directories; policy-makers in countries without registries seeking to better plan for this workforce should seek to initiate their development. Proactive, strategic initiatives and legislation may lead to a better understanding of the existing workforce and could serve to protect all involved – employers, employees and dependent clients. The consequences of inaction – on workers, clients and the global economy – will be significant and universally experienced.

## Additional files

Additional file 1: **PRISMA Checklist.** (DOC 66 kb)
Additional file 2: **Detailed MEDLINE® Search.** (DOCX 118 kb)
Additional file 3: **Characteristics of Included Studies.** (DOCX 336 kb)
Additional file 4: **Synthesis Tracking Tables.** (DOCX 332 kb)

## Abbreviations

ADI: Alzheimer’s Disease International
FTE: Full-time equivalent
HCA: Healthcare aide
HPRD: Hours per resident day
LPN: Licensed Practical Nurse
LTC: Long-term care
NH: Nursing home
NNAS: National Nursing Assistant Survey
OECD: Organisation for Economic and Co-Operative Development
RN: Registered Nurse
USD: United States Dollars
