# Supplementary material for: Invisible no more: a scoping review of the health care aide workforce literature
Source: BMC Nurs. 2015 Jul 22;14:38. doi: 10.1186/s12912-015-0090-x (PMC4511030; doi:10.1186/s12912-015-0090-x)
Supplement: Additional file 2: — Detailed MEDLINE® Search. (DOCX 118 kb) [file 12912_2015_90_MOESM2_ESM.docx]

MEDLINE® In-Process and Other Non-Indexed Citations, Ovid MEDLINE ® Daily and Ovid MEDLINE ® 1946-Present

Search conducted March 16, 2013

1. nurses' aides/ or psychiatric aides/

2. ((unregistered or unregulated or unlicensed) adj (professional* or worker or assistant* or nurs*)).mp. [mp=title, abstract, original title, name of substance word, subject heading word, keyword heading word, protocol supplementary concept, rare disease supplementary concept, unique identifier]

3. (Care aide* or Care Attendant* or Care Guide or paid Caregiver* or professional caregiver or Certified Nursing Assistant* or CNA or CNAs or Client Care Attendant* or Direct care worker or Client Care Attendant* or Geriatric Health Aide* or Health Care Assistant*).tw.

4. (Nursing attendant* or Nursing home aide* or Personal Care Attendant* or Personal Care Nurse* or Personal Care Assistant* or Personal Care Attendant* or Personal support worker* or Residential Aide*).tw.

5. (Health Care Assistant* or Institutional Aide* or Medical Assistant* or Nurses aide* or Nursing assistant* or home support worker or resident care attendant* or continuing care assistant*).tw.

6. or/1-5

7. (og or sn).fs.

8. capacity building/ or health manpower/

9. health services administration/ or "organization and administration"/ or health facility administration/ or exp hospital administration/ or exp personnel management/ or exp "salaries and fringe benefits"/

10. (manpower or workforce or recruitment or education or training or professional development or staff development or retention or loyalty).tw.

11. (administration or human resource* or management or manger* or supervis*).tw.

12. absenteeism/ or efficiency/ or exp "task performance and analysis"/ or time management/ or vocational guidance/

13. (absentee* or job satisfaction* or workload or work load).tw.

14. exp Education, Continuing/ or exp Staff Development/ or exp communication/ or exp income/

15. or/8-14

16. 6 and 15

17. limit 16 to (english language and yr="1995 -Current")

Number of results: 1975
