# Supplementary material for: Invisible no more: a scoping review of the health care aide workforce literature
Source: BMC Nurs. 2015 Jul 22;14:38. doi: 10.1186/s12912-015-0090-x (PMC4511030; doi:10.1186/s12912-015-0090-x)
Supplement: Additional file 3: — Characteristics of Included Studies. (DOCX 336 kb) [file 12912_2015_90_MOESM3_ESM.docx]

| **EDUCATION** | | | | | | | | | | | | | | | | | | | | | | | | | | | | | | | | | | | | | | | | | | | | | | | | | | | | | | | | | | | | | | | | | | | | | | | | | |
| --- | --- | --- | --- | --- | --- | --- | --- | --- | --- | --- | --- | --- | --- | --- | --- | --- | --- | --- | --- | --- | --- | --- | --- | --- | --- | --- | --- | --- | --- | --- | --- | --- | --- | --- | --- | --- | --- | --- | --- | --- | --- | --- | --- | --- | --- | --- | --- | --- | --- | --- | --- | --- | --- | --- | --- | --- | --- | --- | --- | --- | --- | --- | --- | --- | --- | --- | --- | --- | --- | --- | --- | --- | --- |
| **Themes** | **Outcomes** | | | | | | | | | | | | | | | | | | |  | | | | | | | | | | | | | | | | | | | | | | | | | | | | | | | | | | | | | | | | | | | | | | | | | | | **Ref.** | | |
| **Reasons for becoming NA**  **% (SD or SE)** | **By Setting**  Helping others  Want to work in health care  Job security  Other reason | | | | | | | | | | | | | | | | | | | **NH**  63 (0.01)  8 (0.01)  8(0.01)  21(0.01) | | | | | | | | | | | | | | | | | | | | | | | | **Immigrant**  62.5  7.8  6.6  23.2 | | | | | | | | | | | | | | | | | | | | | **Non-Immigrant**  61.9  8.3  8.4  21.4 | | | | | | ^56^  ^33^  ^56^  ^33^  ^56^  ^33^  ^56^  ^33^ | | |
|  | **Most important reason**  Like helping other people  Want to work in health care  Job security  Job readily available or close to home**  Other | | | | | | | | | | | | | | | | | | | **Total**  61.9  8.2  4.3  4.9  20.5 | | | | | | | | | | **Urban**  61.7  8.5  4.2  4.3  21.0 | | | | | | | | | | | | | | | | | | | **Micropolitan**  66.7  6.0  4.5  5.3  17.5 | | | | | | | | | | | | | | | | **Other rural**  57.5  8.8  4.5  8.9  20.1 | | | | | | ^53^ | | |
| **Transition into career as NA**  % | Other Job  School  Home/Children  Unemployed  Other | | | | | | | | | | | | | | | | | | | 52.2  21.7  17.5  4.6  4.1 | | | | | | | | | | | | | | | |  | | | | | | | | | | | | | | | | | | | | | | | | | | | | |  | | | | | | ^53^  ^72^ | | |
|  | Not working, not disabled  Not working, disabled  Not working, in school  Other job (RN, LPN, medical technician, child care, household service, retail, cook) | | | | | | | | | | | | | | | | | | | | | | | | | | | | | | | | | | | 25.1  3.3  3.7  14.9 | | | | | | | | | | | | | | | | | | | | | | | | | | | | |  | | | | | |  |  |  |
|  | **By Location (NH)**  Other Job  School  Home/Children  Unemployed  Other | | | | | | | | | | | | | | | | | | | **Urban**  52.7  21.3  17.8  4.5  3.8 | | | | | | | | | | | | | | | | **Micropolitan**  52.3  22.2  17.2  4.6  3.7 | | | | | | | | | | | | | | | | | | | | | | | | | | | | | **Other Rural**  48.2  23.7  16.1  5.3  6.7 | | | | | | ^53^ | | |
| **Perceptions on**  **Training**  % (SE) | **Initial Training Prepared Them** | | | | | | | | | | | | | | | | | | | **Well** | | | | | | | | | | | | | | | | **Somewhat well** | | | | | | | | | | | | | | | | | | | | | | | | | | | | | **Not at all Well** | | | | | |  | | |
|  |  |  |  |  |  |  |  |  |  |  |  |  |  |  |  |  |  |  |  | >50 | | | | | | | | | | | | | | | |  | | | | | | | | | | | | | | | | | | | | | | | | | | | | |  | | | | | | ^34^ | | |
|  |  |  |  |  |  |  |  |  |  |  |  |  |  |  |  |  |  |  |  | 38 | | | | | | | | | | | | | | | |  | | | | | | | | | | | | | | | | | | | | | | | | | | | | |  | | | | | | ^42^ | | |
|  |  |  |  |  |  |  |  |  |  |  |  |  |  |  |  |  |  |  |  | 96.5 NH | | | | | | | | | | | | | | | | | | | | | | | | | | | | | | | | | | | | | | | | | | | | |  | | | | | | ^33^ | | |
|  |  |  |  |  |  |  |  |  |  |  |  |  |  |  |  |  |  |  |  | 66.2 (1.2) | | | | | | | | | | | | | | | | 30.3 (1.2) | | | | | | | | | | | | | | | | | | | | | | | | | | | | | 3.5 (0.5) | | | | | | ^21^ | | |
|  |  |  |  |  |  |  |  |  |  |  |  |  |  |  |  |  |  |  |  | 66 (n=2221) | | | | | | | | | | | | | | | | 31 (n=2221) | | | | | | | | | | | | | | | | | | | | | | | | | | | | | 3 (n=2221) | | | | | | ^56^ | | |
|  | **HHA** | | | | | | | | | | | | | | | | | | | 81.8 (2.3) | | | | | | | | | | | | | | | |  | | | | | | | | | | | | | | | | | | | | | | | | | | | | |  | | | | | | ^35^ | | |
|  | **CNA** | | | | | | | | | | | | | | | | | | | 65.7 (1.2) | | | | | | | | | | | | | | | |  | | | | | | | | | | | | | | | | | | | | | | | | | | | | |  | | | | | |  |  |  |
|  |  | Tenure>1yr | | | | | | | | | | | | | | | | | | 66 (n=1312) | | | | | | | | | | | | | | | | 31 (n=1312) | | | | | | | | | | | | | | | | | | | | | | 3 (n=1312) | | | | | | | | | | | | | ^56^ | | |
|  |  | Male | | | | | | | | | | | | | | | | | | 64.6 | | | | | | | | | | | | | | | | 33.2 | | | | | | | | | | | | | | | | | | | | | | 2.3 | | | | | | | | | | | | | ^52^ | | |
|  |  | Female | | | | | | | | | | | | | | | | | | 63.7 | | | | | | | | | | | | | | | | 32.9 | | | | | | | | | | | | | | | | | | | | | | 3.4 | | | | | | | | | | | | |  |  |  |
|  |  | Immigrant | | | | | | | | | | | | | | | | | | 98.4 NH | | | | | | | | | | | | | | | | | | | | | | | | | | | | | | | | | | | | | |  | | | | | | | | | | | | | ^33^ | | |
|  |  | Non Immigrant | | | | | | | | | | | | | | | | | | 96.1 NH | | | | | | | | | | | | | | | | | | | | | | | | | | | | | | | | | | | | | |  | | | | | | | | | | | | |  |  |  |
| **Current Initial Training Topics in Curriculum**  Mean (N) 5=very important  1=not important | **Importance of Topic** | | | | | | | | | | | | | | | | | | | | | | | | | | | | | | | | | | | **Home Based Providers** | | | | | | | | | | | | | | | | | | | | | | | | | | | | | **Residential Care Providers** | | | | | | ^28^  ^28^ | | |
|  | **Patient Care** | | | | | Personal Care | | | | | | | | | | | | | | | | | | | | | | | | | | | | | | 4.6 (11) | | | | | | | | | | | | | | | | | | | | | | | | | | | | | 4.9 (37) | | | | | |  |  |  |
|  |  |  |  |  |  | Advanced Personal Care | | | | | | | | | | | | | | | | | | | | | | | | | | | | | | 3.6 (8) | | | | | | | | | | | | | | | | | | | | | | | | | | | | | 3.7 (33) | | | | | |  |  |  |
|  |  |  |  |  |  | First Aid | | | | | | | | | | | | | | | | | | | | | | | | | | | | | | 4.5 (11) | | | | | | | | | | | | | | | | | | | | | | | | | | | | | 4.5 (32) | | | | | |  |  |  |
|  |  |  |  |  |  | Lifting/Handling | | | | | | | | | | | | | | | | | | | | | | | | | | | | | | 4.5 (10) | | | | | | | | | | | | | | | | | | | | | | | | | | | | | 4.9 (41) | | | | | |  |  |  |
|  |  |  |  |  |  | Fall Prevention | | | | | | | | | | | | | | | | | | | | | | | | | | | | | | 3.9 (7) | | | | | | | | | | | | | | | | | | | | | | | | | | | | | 4.8 (36) | | | | | |  |  |  |
|  |  |  |  |  |  | Medications | | | | | | | | | | | | | | | | | | | | | | | | | | | | | | 3.9 (7) | | | | | | | | | | | | | | | | | | | | | | | | | | | | | 4.9 (40) | | | | | |  |  |  |
|  |  |  |  |  |  | Nutrition | | | | | | | | | | | | | | | | | | | | | | | | | | | | | | 4.0 (9) | | | | | | | | | | | | | | | | | | | | | | | | | | | | | 4.5 (32) | | | | | |  |  |  |
|  |  |  |  |  |  | Continence | | | | | | | | | | | | | | | | | | | | | | | | | | | | | | 3.7 (9) | | | | | | | | | | | | | | | | | | | | | | | | | | | | | 4.7 (38) | | | | | |  |  |  |
|  |  |  |  |  |  | Oral Hygiene | | | | | | | | | | | | | | | | | | | | | | | | | | | | | | 3.5 (8) | | | | | | | | | | | | | | | | | | | | | | | | | | | | | 4.5 (37) | | | | | |  |  |  |
|  | **Holistic Care** | | | | | Recognizing Abuse | | | | | | | | | | | | | | | | | | | | | | | | | | | | | | 4.3 (10) | | | | | | | | | | | | | | | | | | | | | | | | | | | | | 4.6 (40) | | | | | |  |  |  |
|  |  |  |  |  |  | Philosophy and Values | | | | | | | | | | | | | | | | | | | | | | | | | | | | | | 4.3 (8) | | | | | | | | | | | | | | | | | | | | | | | | | | | | | 4.7 (40) | | | | | |  |  |  |
|  |  |  |  |  |  | Cultural Safety | | | | | | | | | | | | | | | | | | | | | | | | | | | | | | 4.2 (12) | | | | | | | | | | | | | | | | | | | | | | | | | | | | | 3.8 (40) | | | | | |  |  |  |
|  |  |  |  |  |  | Housework | | | | | | | | | | | | | | | | | | | | | | | | | | | | | | 4.1 (8) | | | | | | | | | | | | | | | | | | | | | | | | | | | | | 3.4 (27) | | | | | |  |  |  |
|  |  |  |  |  |  | Common Disabilities | | | | | | | | | | | | | | | | | | | | | | | | | | | | | | 4.0 (11) | | | | | | | | | | | | | | | | | | | | | | | | | | | | | 4.3 (38) | | | | | |  |  |  |
|  |  |  |  |  |  | Physical Rehab | | | | | | | | | | | | | | | | | | | | | | | | | | | | | | 3.6 (8) | | | | | | | | | | | | | | | | | | | | | | | | | | | | | 4.1 (35) | | | | | |  |  |  |
|  |  |  |  |  |  | Social Rehab | | | | | | | | | | | | | | | | | | | | | | | | | | | | | | 3.6 (7) | | | | | | | | | | | | | | | | | | | | | | | | | | | | | 4.6 (37) | | | | | |  |  |  |
|  |  |  |  |  |  | Sexuality | | | | | | | | | | | | | | | | | | | | | | | | | | | | | | 2.2 (6) | | | | | | | | | | | | | | | | | | | | | | | | | | | | | 4.0 (38) | | | | | |  |  |  |
|  | **Provider & Policy** | | | | | Personal safety | | | | | | | | | | | | | | | | | | | | | | | | | | | | | | 4.5 (10) | | | | | | | | | | | | | | | | | | | | | | | | | | | | | 4.7 (38) | | | | | |  |  |  |
|  |  |  |  |  |  | Emergency Procedures | | | | | | | | | | | | | | | | | | | | | | | | | | | | | | 4.6 (9) | | | | | | | | | | | | | | | | | | | | | | | | | | | | | 5.0 (42) | | | | | |  |  |  |
|  |  |  |  |  |  | Infection Control | | | | | | | | | | | | | | | | | | | | | | | | | | | | | | 4.4 (9) | | | | | | | | | | | | | | | | | | | | | | | | | | | | | 5.0 (40) | | | | | |  |  |  |
|  |  |  |  |  |  | Service Policy/Protocol | | | | | | | | | | | | | | | | | | | | | | | | | | | | | | 3.7 (9) | | | | | | | | | | | | | | | | | | | | | | | | | | | | | 4.4 (37) | | | | | |  |  |  |
|  |  |  |  |  |  | Risk Management | | | | | | | | | | | | | | | | | | | | | | | | | | | | | | 3.8 (8) | | | | | | | | | | | | | | | | | | | | | | | | | | | | | 4.6 (35) | | | | | |  |  |  |
|  |  |  |  |  |  | Fire and Safety | | | | | | | | | | | | | | | | | | | | | | | | | | | | | | 4.1 (9) | | | | | | | | | | | | | | | | | | | | | | | | | | | | | 5.0 (4.1) | | | | | |  |  |  |
| **Perception of Initial Training by Topic**  % |  | | | | | **Rated Excellent** | | | | | | | | | | | | | | **Total** | | | | | | | | | | | | | | | | **Immigrant** | | | | | | | | | | | | | | | | | | | | | | | | | | | | | **Non immigrant** | | | | | |  | | |
|  | **Patient Care** | | | | | Resident Care | | | | | | | | | | | | | | 66.6 | | | | | | | | | | | | | | | |  | | | | | | | | | | | | | | | | | | | | | | | | | | | | |  | | | | | | ^21^ | | |
|  |  |  |  |  |  |  | | | | | | | | | | | | | | 94.4 | | | | | | | | | | | | | | | | 96.2 | | | | | | | | | | | | | | | | | | | | | | | | | | | | | 93.9 | | | | | | ^33^ | | |
|  |  |  |  |  |  | Talk with Residents | | | | | | | | | | | | | | 57.9 | | | | | | | | | | | | | | | |  | | | | | | | | | | | | | | | | | | | | | | | | | | | | |  | | | | | | ^21^ | | |
|  |  |  |  |  |  |  | | | | | | | | | | | | | | 90.8 | | | | | | | | | | | | | | | | 94.2 | | | | | | | | | | | | | | | | | | | | | | | | | | | | | 90.0 | | | | | | ^33^ | | |
|  | **Holistic Care** | | | | | Dementia Care | | | | | | | | | | | | | | 44.6 | | | | | | | | | | | | | | | |  | | | | | | | | | | | | | | | | | | | | | | | | | | | | |  | | | | | | ^21^ | | |
|  |  |  |  |  |  |  | | | | | | | | | | | | | | 80.8 | | | | | | | | | | | | | | | | 90.7 | | | | | | | | | | | | | | | | | | | | | | | | | | | | | 78.3 | | | | | | ^33^ | | |
|  |  |  |  |  |  | Discuss Resident Care with Family | | | | | | | | | | | | | | 42.2 | | | | | | | | | | | | | | | |  | | | | | | | | | | | | | | | | | | | | | | | | | | | | |  | | | | | | ^21^ | | |
|  |  |  |  |  |  |  | | | | | | | | | | | | | | 75.6 | | | | | | | | | | | | | | | | 83.1 | | | | | | | | | | | | | | | | | | | | | | | | | | | | | 73.8 | | | | | | ^33^ | | |
|  |  |  |  |  |  | Work with Abusive Residents | | | | | | | | | | | | | | 41.9 | | | | | | | | | | | | | | | |  | | | | | | | | | | | | | | | | | | | | | | | | | | | | |  | | | | | | ^21^ | | |
|  |  |  |  |  |  |  | | | | | | | | | | | | | | 76.3 | | | | | | | | | | | | | | | | 85.8 | | | | | | | | | | | | | | | | | | | | | | | | | | | | | 73.9 | | | | | | ^33^ | | |
|  | **Provider & Policy** | | | | | Record Resident Info | | | | | | | | | | | | | | 52.8 | | | | | | | | | | | | | | | |  | | | | | | | | | | | | | | | | | | | | | | | | | | | | |  | | | | | | ^21^ | | |
|  |  |  |  |  |  |  | | | | | | | | | | | | | | 88.9 | | | | | | | | | | | | | | | | 89.5 | | | | | | | | | | | | | | | | | | | | | | | | | | | | | 88.7 | | | | | | ^33^ | | |
|  |  |  |  |  |  | Prevent Work Injuries | | | | | | | | | | | | | | 51.9 | | | | | | | | | | | | | | | |  | | | | | | | | | | | | | | | | | | | | | | | | | | | | |  | | | | | | ^21^ | | |
|  |  |  |  |  |  |  | | | | | | | | | | | | | | 90.2 | | | | | | | | | | | | | | | | 94.9 | | | | | | | | | | | | | | | | | | | | | | | | | | | | | 89.0 | | | | | | ^33^ | | |
|  |  |  |  |  |  | Organize Tasks | | | | | | | | | | | | | | 49.4 | | | | | | | | | | | | | | | |  | | | | | | | | | | | | | | | | | | | | | | | | | | | | |  | | | | | | ^21^ | | |
|  |  |  |  |  |  |  | | | | | | | | | | | | | | 85.0 | | | | | | | | | | | | | | | | 93.8 | | | | | | | | | | | | | | | | | | | | | | | | | | | | | 82.6 | | | | | | ^33^ | | |
|  |  |  |  |  |  | Work with Supervisors | | | | | | | | | | | | | | 39.8 | | | | | | | | | | | | | | | |  | | | | | | | | | | | | | | | | | | | | | | | | | | | | |  | | | | | | ^21^ | | |
|  |  |  |  |  |  |  | | | | | | | | | | | | | | 81.5 | | | | | | | | | | | | | | | | 88.3 | | | | | | | | | | | | | | | | | | | | | | | | | | | | | 79.7 | | | | | | ^33^ | | |
|  |  |  |  |  |  | Work with Coworkers | | | | | | | | | | | | | | 37.3 | | | | | | | | | | | | | | | |  | | | | | | | | | | | | | | | | | | | | | | | | | | | | |  | | | | | | ^21^ | | |
|  |  |  |  |  |  |  | | | | | | | | | | | | | | 79.2 | | | | | | | | | | | | | | | | 90.0 | | | | | | | | | | | | | | | | | | | | | | | | | | | | | 76.5 | | | | | | ^33^ | | |
|  |  |  |  |  |  | Problem solve work issues | | | | | | | | | | | | | | 32.2 | | | | | | | | | | | | | | | |  | | | | | | | | | | | | | | | | | | | | | | | | | | | | |  | | | | | | ^21^ | | |
|  |  |  |  |  |  |  | | | | | | | | | | | | | | 74.8 | | | | | | | | | | | | | | | | 86.2 | | | | | | | | | | | | | | | | | | | | | | | | | | | | | 72.0 | | | | | | ^33^ | | |
| **Requested Topics**  % (SE) |  | | | | |  | | | | | | | | | | | | | | | | | | | | | | | | | | | | | | **For Initial Training** | | | | | | | | | | | | | | | | | | | | | | | | | | | | | | | | | | |  | | |
|  | **Patient Care** | | | | | Care Skills | | | | | | | | | | | | | | | | | | | | | | | | | | | | | | 11.4 (1.5) | | | | | | | | | | | | | | | | | | | | | | | | | | | | |  | | | | | | ^21,29^ | | |
|  |  |  |  |  |  | Talk with residents | | | | | | | | | | | | | | | | | | | | | | | | | | | | | |  | | | | | | | | | | | | | | | | | | | | | | | | | | | | |  | | | | | | ^21^ | | |
|  |  |  |  |  |  | Medication Management | | | | | | | | | | | | | | | | | | | | | | | | | | | | | |  | | | | | | | | | | | | | | | | | | | | | | | | | | | | |  | | | | | |  | | |
|  |  |  |  |  |  | Pain Management | | | | | | | | | | | | | | | | | | | | | | | | | | | | | |  | | | | | | | | | | | | | | | | | | | | | | | | | | | | |  | | | | | |  | | |
|  | **Holistic Care** | | | | | Abusive Residents | | | | | | | | | | | | | | | | | | | | | | | | | | | | | | 18.7 (1.9) | | | | | | | | | | | | | | | | | | | | | | | | | | | | |  | | | | | | ^21,29^ | | |
|  |  |  |  |  |  | Discuss resident care with family members | | | | | | | | | | | | | | | | | | | | | | | | | | | | | | 7.3 (1.2) | | | | | | | | | | | | | | | | | | | | | | | | | | | | |  | | | | | | ^21^ | | |
|  |  |  |  |  |  | Work with Residents Family | | | | | | | | | | | | | | | | | | | | | | | | | | | | | |  | | | | | | | | | | | | | | | | | | | | | | | | | | | | |  | | | | | |  |  |  |
|  |  |  |  |  |  | Dementia Care | | | | | | | | | | | | | | | | | | | | | | | | | | | | | | 18.0 (1.9) | | | | | | | | | | | | | | | | | | | | | | | | | | | | |  | | | | | |  | | |
|  |  |  |  |  |  | Mental Health training | | | | | | | | | | | | | | | | | | | | | | | | | | | | | |  | | | | | | | | | | | | | | | | | | | | | | | | | | | | |  | | | | | | ^29^ | | |
|  |  |  |  |  |  | Integrating Health Promotion | | | | | | | | | | | | | | | | | | | | | | | | | | | | | |  | | | | | | | | | | | | | | | | | | | | | | | | | | | | |  | | | | | |  | | |
|  |  |  |  |  |  | Multicultural training | | | | | | | | | | | | | | | | | | | | | | | | | | | | | |  | | | | | | | | | | | | | | | | | | | | | | | | | | | | |  | | | | | |  |  |  |
|  |  |  |  |  |  | Challenging Behavior skills | | | | | | | | | | | | | | | | | | | | | | | | | | | | | |  | | | | | | | | | | | | | | | | | | | | | | | | | | | | |  | | | | | |  |  |  |
|  | **Provider & Policy** | | | | | Work with coworkers | | | | | | | | | | | | | | | | | | | | | | | | | | | | | | 11.3 (1.6) | | | | | | | | | | | | | | | | | | | | | | | | | | | | |  | | | | | | ^21^ | | |
|  |  |  |  |  |  | Organize work tasks | | | | | | | | | | | | | | | | | | | | | | | | | | | | | | 11.2 (1.7) | | | | | | | | | | | | | | | | | | | | | | | | | | | | |  | | | | | |  | | |
|  |  |  |  |  |  | Work with supervisors | | | | | | | | | | | | | | | | | | | | | | | | | | | | | | 8.4 (1.6) | | | | | | | | | | | | | | | | | | | | | | | | | | | | |  | | | | | |  | | |
|  |  |  |  |  |  | Problem solving for work issues | | | | | | | | | | | | | | | | | | | | | | | | | | | | | | 5.0 (1.0) | | | | | | | | | | | | | | | | | | | | | | | | | | | | |  | | | | | |  | | |
|  |  |  |  |  |  | Record resident information | | | | | | | | | | | | | | | | | | | | | | | | | | | | | | 3.7 (0.9) | | | | | | | | | | | | | | | | | | | | | | | | | | | | |  | | | | | |  | | |
|  |  |  |  |  |  | Prevent work injuries | | | | | | | | | | | | | | | | | | | | | | | | | | | | | | 3.3 (0.9) | | | | | | | | | | | | | | | | | | | | | | | | | | | | |  | | | | | |  | | |
|  |  |  |  |  |  | Being physically capable to perform role | | | | | | | | | | | | | | | | | | | | | | | | | | | | | |  | | | | | | | | | | | | | | | | | | | | | | | | | | | | |  | | | | | | ^31^ | | |
|  |  |  |  |  |  | Training in management | | | | | | | | | | | | | | | | | | | | | | | | | | | | | |  | | | | | | | | | | | | | | | | | | | | | | | | | | | | |  | | | | | |  |  |  |
|  |  |  |  |  |  | Stress management | | | | | | | | | | | | | | | | | | | | | | | | | | | | | |  | | | | | | | | | | | | | | | | | | | | | | | | | | | | |  | | | | | |  |  |  |
| **Location of Initial or vocational Training**  % (SE) | **Facility Employing** | | | | | | | | | | | | | | | | | | | | 55.7 (NH) | | | | | | | | | | | | | | | | | | | | | | | | | | | | | | | | | | | | | | | | | | | | | | | | | ^21^ | |  |  |
|  |  |  |  |  |  |  |  |  |  |  |  |  |  |  |  |  |  |  |  |  | **Total** | | | | | | | | | **Urban** | | | | | | | | | | | | | | | | **Micropolitan** | | | | | | | | | | | | | | | | | | | | **Other Rural** | | | |  | |  |  |
|  |  |  |  |  |  |  |  |  |  |  |  |  |  |  |  |  |  |  |  |  | 55.1 | | | | | | | | | | | 52.5 | | | | | | | | | | | | | | | | | | | 61.4** | | | | | | | | | | | | | | | 65.3** | | | | ^53^ | |  |  |
|  |  |  |  |  |  |  |  |  |  |  |  |  |  |  |  |  |  |  |  |  | **CNA** | | | | | | | | | | **HHA** | | | | | | | | | | | | | | | | | | | | | | | | | | | | |  | | | | | | | | | |  | |  |  |
|  |  |  |  |  |  |  |  |  |  |  |  |  |  |  |  |  |  |  |  |  | 55 | | | | | | | | | | 47 | | | | | | | | | | | | | | | | | | | | | | | | | | | | |  |  |  |  |  |  |  |  |  |  |  |  |  |  |
|  |  |  |  |  |  |  |  |  |  |  |  |  |  |  |  |  |  |  |  |  | **Initial Training** | | | | | | | | | | | | | | | | | | | | | | | | | | | | | | | | | | | | | | |  | | | | | | | | | | ^35^ | |  |  |
|  |  |  |  |  |  |  |  |  |  |  |  |  |  |  |  |  |  |  |  |  | 55.7(1.5) | | | | | | | | | | | | | | | | | | | | | | | | | | | | | | | | | | | | | | |  | | | | | | | | | | ^21^ | |  |  |
|  |  |  |  |  |  |  |  |  |  |  |  |  |  |  |  |  |  |  |  |  | **Assisted Living** | | | | | | | | | | | | | | | | | | | | | | **LTC** | | | | | | | | | | | | | | | | | **Other Facility Type** | | | | | | | | | |  | |  |  |
|  |  |  |  |  |  |  |  |  |  |  |  |  |  |  |  |  |  |  |  |  | 49 | | | | | | | | | | | | | | | | | | | | | | 70 | | | | | | | | | | | | | | | | | 47 | | | | | | | | | | ^14^ | |  |  |
|  |  |  |  |  |  |  |  |  |  |  |  |  |  |  |  |  |  |  |  |  | **Total** | | | | | | | | | | | | | | | | | | | | | | **Immigrant** | | | | | | | | | | | | | | | | | **Non Immigrant** | | | | | | | | | |  | |  |  |
|  |  |  |  |  |  |  |  |  |  |  |  |  |  |  |  |  |  |  |  |  | 55.1 | | | | | | | | | | | | | | | | | | | | | | 43.1* | | | | | | | | | | | | | | | | | 58.0* | | | | | | | | | | ^33^ | |  |  |
|  |  |  |  |  |  |  |  |  |  |  |  |  |  |  |  |  |  |  |  |  | Obtained through 6 month in house training | | | | | | | | | | | | | | | | | | | | | | | | | | | | | | | | | | | | | | | | | | | | | | | | | | ^45^ | | |
|  |  |  |  |  |  |  |  |  |  |  |  |  |  |  |  |  |  |  |  |  | Local acute trusts have begun to implement their own training schemes in response to the individual needs of specific clinical areas. | | | | | | | | | | | | | | | | | | | | | | | | | | | | | | | | | | | | | | | | | | | | | | | | | |  |  |  |
|  |  |  |  |  |  |  |  |  |  |  |  |  |  |  |  |  |  |  |  |  | Trained informally by nurses in the clinical environment. | | | | | | | | | | | | | | | | | | | | | | | | | | | | | | | | | | | | | | | | | | | | | | | | | | ^47^ | | |
|  |  |  |  |  |  |  |  |  |  |  |  |  |  |  |  |  |  |  |  |  | NVQs involve competency-based learning with on the job training. | | | | | | | | | | | | | | | | | | | | | | | | | | | | | | | | | | | | | | | | | | | | | | | | | |  |  |  |
|  |  |  |  |  |  |  |  |  |  |  |  |  |  |  |  |  |  |  |  |  | Offered informal in- house training | | | | | | | | | | | | | | | | | | | | | | | | | | | | | | | | | | | | | | | | | | | | | | | | | | ^29^ | | |
|  |  |  |  |  |  |  |  |  |  |  |  |  |  |  |  |  |  |  |  |  | In house or NVQ level 1-4 most predominant form of education in intermediate care setting | | | | | | | | | | | | | | | | | | | | | | | | | | | | | | | | | | | | | | | | | | | | | | | | | | ^18^ | | |
|  |  |  |  |  |  |  |  |  |  |  |  |  |  |  |  |  |  |  |  |  | Reduced prevalence of NH-based programs during the study period  Most CNAs are now receiving their pre-employment training outside the NH setting | | | | | | | | | | | | | | | | | | | | | | | | | | | | | | | | | | | | | | | | | | | | | | | | | | ^26^ | | |
|  | **Community College** | | | | | | | | | | | | | | | | | | | In Canada required formal training usually at college level | | | | | | | | | | | | | | | | | | | | | | | | | | | | | | | | | | | | | | | | | | | | | | | | | | | ^44^ | | |
|  |  | | | | | | | | | | | | | | | | | | | **Total** | | | | | | | | **Urban** | | | | | | | | | | | | | | | | | | | **Micropolitan** | | | | | | | | | | | | | | | | | | **Other Rural** | | | | | | ^53^ | | |
|  |  |  |  |  |  |  |  |  |  |  |  |  |  |  |  |  |  |  |  | 17.9 | | | | | | | | 16.7 | | | | | | | | | | | | | | | | | | | 23.8** | | | | | | | | | | | | | | | | | | 18.7** | | | | | |  |  |  |
|  |  |  |  |  |  |  |  |  |  |  |  |  |  |  |  |  |  |  |  | **Initial Training** | | | | | | | | | | | | | | | | | | | | | | | | | | | | | | | | | | | | | | |  | | | | | | | | | | | |  | |  |
|  |  |  |  |  |  |  |  |  |  |  |  |  |  |  |  |  |  |  |  | 19.4(1.1) | | | | | | | | | | | | | | | | | | | | | | | | | | | | | | | | | | | | | | |  | | | | | | | | | | | | ^21^ | |  |
|  |  |  |  |  |  |  |  |  |  |  |  |  |  |  |  |  |  |  |  | **Total** | | | | | | | | | | | | | | | | | | **Immigrant** | | | | | | | | | | | | | | | | | | | **Non Immigrant** | | | | | | | | | | | | | |  | |  |
|  |  |  |  |  |  |  |  |  |  |  |  |  |  |  |  |  |  |  |  | 17.9 | | | | | | | | | | | | | | | | | | 15.7* | | | | | | | | | | | | | | | | | | | 18.6* | | | | | | | | | | | | | | ^33^ | |  |
|  | **High School** | | | | | | | | | | | | | | | | | | | **Total** | | | | | | | | **Urban** | | | | | | | | | | | | | | | | | | | **Micropolitan** | | | | | | | | | | | | | | | | | | **Other Rural** | | | | | | ^53^ | | |
|  |  |  |  |  |  |  |  |  |  |  |  |  |  |  |  |  |  |  |  | 6.1 | | | | | | | | 6.1 | | | | | | | | | | | | | | | | | | | 6.0** | | | | | | | | | | | | | | | | | | 6.3** | | | | | |  |  |  |
|  |  |  |  |  |  |  |  |  |  |  |  |  |  |  |  |  |  |  |  |  | | | | | | | |  | | | | | | | | | | | | | | | | | | |  | | | | | | | | | | | | | | | | | |  | | | | | |  | | |
|  |  |  |  |  |  |  |  |  |  |  |  |  |  |  |  |  |  |  |  | **Initial Training** | | | | | | | | | | | | | | | | | | | | | | | | | | |  | | | | | | | | | | | | | | | | | | | | | | | |  | |  |
|  |  |  |  |  |  |  |  |  |  |  |  |  |  |  |  |  |  |  |  | 6.2(0.7) | | | | | | | | | | | | | | | | | | | | | | | | | | |  | | | | | | | | | | | | | | | | | | | | | | | | ^21^ | |  |
|  | **Vocational or Trade School** | | | | | | | | | | | | | | | | | | | **Total** | | | | | | | | **Urban** | | | | | | | | | | | | | | | | | | | **Micropolitan** | | | | | | | | | | | | | | | | | | **Other Rural** | | | | | | ^53^ | | |
|  |  |  |  |  |  |  |  |  |  |  |  |  |  |  |  |  |  |  |  | 6.0 | | | | | | | | 6.6 | | | | | | | | | | | | | | | | | | | 5.4** | | | | | | | | | | | | | | | | | | 2.7** | | | | | |  |  |  |
|  | **Other/Not Specified** | | | | | | | | | | | | | | | | | | | **Total** | | | | | | | | **Urban** | | | | | | | | | | | | | | | | | | | **Micropolitan** | | | | | | | | | | | | | | | | | | **Other Rural** | | | | | | ^53^ | | |
|  |  |  |  |  |  |  |  |  |  |  |  |  |  |  |  |  |  |  |  | 14.9 | | | | | | | | 18.0 | | | | | | | | | | | | | | | | | | | 3.3** | | | | | | | | | | | | | | | | | | 7.0** | | | | | |  |  |  |
|  |  |  |  |  |  |  |  |  |  |  |  |  |  |  |  |  |  |  |  | **CNA** | | | | | | | | **HHA** | | | | | | | | | | | | | | | | | | |  | | | | | | | | | | | | | | | | | | | | | | | | ^35^ | |  |
|  |  |  |  |  |  |  |  |  |  |  |  |  |  |  |  |  |  |  |  | 21 | | | | | | | | 4.9 | | | | | | | | | | | | | | | | | | |  |  |  |  |  |  |  |  |  |  |  |  |  |  |  |  |  |  |  |  |  |  |  |  |  |  |  |
|  |  |  |  |  |  |  |  |  |  |  |  |  |  |  |  |  |  |  |  | **Total** | | | | | | | | | | | | | | | | | | | **Immigrant** | | | | | | | | | | | | | | | | | | **Non Immigrant** | | | | | | | | | | | | | |  | |  |
|  |  |  |  |  |  |  |  |  |  |  |  |  |  |  |  |  |  |  |  | 27.0 | | | | | | | | | | | | | | | | | | | 41.2* | | | | | | | | | | | | | | | | | | 23.4* | | | | | | | | | | | | | | ^33^ | |  |
|  |  |  |  |  |  |  |  |  |  |  |  |  |  |  |  |  |  |  |  | Unaccredited/private “Universities” | | | | | | | | | | | | | | | | | | | | | | | | | | | | | | | | | | | | | | | | | | | | | | | | | | | ^40^ | |  |
| **Cost of training**  % | **Paid by Employer** | | | | | | |  | | | | | | | | | | | | | | 78 | | | | | | | | | | | | | | | | | | | | | | | | | | | | | | | | | | | | | | | | | | | | | | | | | ^28^ | | |
|  |  |  |  |  |  |  |  |  | | | | | | | | | | | | | | **Total** | | | | | **Urban** | | | | | | | | | | | | | | | | | | | | | **Micropolitan** | | | | | | | | | | | | | | | | | **Other Rural** | | | | | | ^53^ | | |
|  |  |  |  |  |  |  |  |  |  |  |  |  |  |  |  |  |  |  |  |  |  | 70.1 | | | | | 67.9 | | | | | | | | | | | | | | | | | | | | | 74.6** | | | | | | | | | | | | | | | | | 77.9** | | | | | |  |  |  |
|  |  |  |  |  |  |  |  |  | | | | | | | | | | | | | | **Initial Training** | | | | | | | | | | | | | | | | | | | | | | | | | | | | | |  | | | | | | | | | | | | | | | | | | | ^21^ | | |
|  |  |  |  |  |  |  |  | Partial | | | | | | | | | | | | | | 55.2 (1.6) | | | | | | | | | | | | | | | | | | | | | | | | | | | | | |  | | | | | | | | | | | | | | | | | | |  |  |  |
|  | **Paid by Employee** | | | | | | |  | | | | | | | | | | | | | | **Total** | | | | | **Urban** | | | | | | | | | | | | | | | | | | | | | **Micropolitan** | | | | | | | | | | | | | | | | | **Other Rural** | | | | | | ^53^ | | |
|  |  |  |  |  |  |  |  | All | | | | | | | | | | | | | | 27.0 | | | | | 29.4 | | | | | | | | | | | | | | | | | | | | | 18.1** | | | | | | | | | | | | | | | | | 21.0** | | | | | |  |  |  |
|  |  |  |  |  |  |  |  | Part | | | | | | | | | | | | | | 8.5 | | | | | 9.0 | | | | | | | | | | | | | | | | | | | | | 6.2** | | | | | | | | | | | | | | | | | 8.1** | | | | | |  |  |  |
|  |  |  |  |  |  |  |  | None | | | | | | | | | | | | | | 64.6 | | | | | 61.7 | | | | | | | | | | | | | | | | | | | | | 75.7** | | | | | | | | | | | | | | | | | 70.9** | | | | | |  |  |  |
|  |  |  |  |  |  |  |  |  | | | | | | | | | | | | | | Most CNAs are now paying a greater share of their training costs | | | | | | | | | | | | | | | | | | | | | | | | | | | | | | | | | | | | | | | | | | | | | | | | | ^26^ | | |
|  | **Paid by Other** | | | | | | |  | | | | | | | | | | | | | | **Total** | | | | | **Urban** | | | | | | | | | | | | | | | | | | | | | **Micropolitan** | | | | | | | | | | | | | | | | | **Other Rural** | | | | | | ^53^ | | |
|  |  |  |  |  |  |  |  |  |  |  |  |  |  |  |  |  |  |  |  |  |  | 29.9 | | | | | 32.1 | | | | | | | | | | | | | | | | | | | | | 25.4 | | | | | | | | | | | | | | | | | 22.1 | | | | | |  |  |  |
|  | **Factors Associated with Cost** | | | | | | | 48.0 (1.8) | | | | | | | | | | | | | | Monetary Incentive would encourage increased training | | | | | | | | | | | | | | | | | | | | | | | | | | | | | | | | | | | | | | | | | | | | | | | | | ^21^ | | |
|  |  |  |  |  |  |  |  | 92.2 (0.9) | | | | | | | | | | | | | | Nonmonetary incentive (eg. convenience) would encourage increased training | | | | | | | | | | | | | | | | | | | | | | | | | | | | | | | | | | | | | | | | | | | | | | | | |  |  |  |
|  |  |  |  |  |  |  |  |  | | | | | | | | | | | | | | Providers indicated biggest barriers for poor attendance are lack of funding and problem releasing staff | | | | | | | | | | | | | | | | | | | | | | | | | | | | | | | | | | | | | | | | | | | | | | | | | ^28^ | | |
|  |  |  |  |  |  |  |  |  | | | | | | | | | | | | | | Funding for SW comes from local authorities, social services, in-house budgets and usually requires a combination of sources | | | | | | | | | | | | | | | | | | | | | | | | | | | | | | | | | | | | | | | | | | | | | | | | | ^18^ | | |
| **Training Hours**  Median Hours  Ratio (%)  % total respondents |  | | | | | | | | | | | | | | | | **Overall** | | | | | | | | | | | | **NH** | | | | | | | | | | | | | | | **Assisted Living** | | | | | | | | | | | | | | **Other LTC** | | | | | | | | | **Other Facilities** | | | |  | | |
|  | **Total training hours** | | | | | | | | | | | | | | | | 56 | | | | | | | | | | | | | 80 | | | | | | | | | | | | | | 56 | | | | | | | | | | | | | | 40 | | | | | | | | | 21.5 | | | | ^14^ | | |
|  | **Classroom:**  **Clinical** | | | | | | | | | | | | | | | | 74:26 | | | | | | | | | | | | | 75:25 | | | | | | | | | | | | | | 71:29 | | | | | | | | | | | | | | 95:5 | | | | | | | | | 83:17 | | | |  | | |
|  |  | | | | | | | | | |  | | | | | | 82:18 | | | | | | | | | | | | | | | | | | | | | | | | | | | | | | | | | | | | | | | | | | | | | | | | | | | | | | ^72^ | | |
|  | Home Helper 3 | | | | | | | | | | | | | | | |  | | | | | | | | | | | | | | | | | | | | | | | |  | | | | | | | | | | | | | | | | | |  | | | | | | | | | 50:50 | | | ^69^ | | |
|  | With classroom | | | | | | | | | | | | | | | | 19.5 (1.1) | | | | | | | | | | | | | | | | | | | | | | | |  | | | | | | | | | | | | | | | | | | | | | | | | | | | | | | ^21^ | | |
|  | With clinical | | | | | | | | | | | | | | | | 27.4(1.2) | | | | | | | | | | | | | | | | | | | | | | | |  | | | | | | | | | | | | | | | | | | | | | | | | | | | | | |  | | |
|  | With both | | | | | | | | | | | | | | | | 53.1(1.3) | | | | | | | | | | | | | | | | | | | | | | | |  | | | | | | | | | | | | | | | | | | | | | | | | | | | | | |  | | |
| **Regulations**  % | **State** | | | | | | 71 (n=29)  32 (n=13) | | | | | | | | | | | **US** - clear mechanisms for reporting inappropriate use of UAPs  - Of state/jurisdiction, respondents **without** guidelines/regulation for RNs who supervised UAPs | | | | | | | | | | | | | | | | | | | | | | | | | | | | | | | | | | | | | | | | | | | | | | | | | | | | | ^25^ | | |
|  |  |  |  |  |  |  |  | | | | | | | | | | | **Japan** - Ministry of Health and Welfare (1995) sets guidelines for training requirements | | | | | | | | | | | | | | | | | | | | | | | | | | | | | | | | | | | | | | | | | | | | | | | | | | | | | ^69^ | | |
|  |  |  |  |  |  |  |  | | | | | | | | | | | **US**- Overall findings suggest that regulatory modifications could be beneficial to improve resident care outcomes in nursing homes | | | | | | | | | | | | | | | | | | | | | | | | | | | | | | | | | | | | | | | | | | | | | | | | | | | | | ^71^ | | |
|  |  | | | | | |  | | | | | | | | | | | - Only 4 states required additional yearly CE hours to maintain CNA certification | | | | | | | | | | | | | | | | | | | | | | | | | | | | | | | | | | | | | | | | | | | | | | | | | | | | |  |  |  |
|  | **Individual** | | | | | | 83 (n=34) | | | | | | | | | | | **US -** Of state boards, reported standardize curriculum in place for UAPs | | | | | | | | | | | | | | | | | | | | | | | | | | | | | | | | | | | | | | | | | | | | | | | | | | | | | ^25^ | | |
|  |  | | | | | | 63 | | | | | | | | | | | No plans existed for developing a standardized curriculum for UAP preparation | | | | | | | | | | | | | | | | | | | | | | | | | | | | | | | | | | | | | | | | | | | | | | | | | | | | |  | | |
|  |  | | | | | | 90 | | | | | | | | | | | **New Zealand -**Of providers indicated that training was necessary but that there were no legal consequences of non-attendance | | | | | | | | | | | | | | | | | | | | | | | | | | | | | | | | | | | | | | | | | | | | | | | | | | | | | ^28^ | | |
|  |  | | | | | |  | | | | | | | | | | | **Canada -**“HSWs in Canada are not regulated by any professional or governmental regulatory body” | | | | | | | | | | | | | | | | | | | | | | | | | | | | | | | | | | | | | | | | | | | | | | | | | | | | | ^44^ | | |
|  | **Registration & Licensure** | | | | | | | | | | | | | | | | | **Scotland** - HCAs are registered on voluntary basis but nowhere else in the UK | | | | | | | | | | | | | | | | | | | | | | | | | | | | | | | | | | | | | | | | | | | | | | | | | | | | | ^47^ | | |
|  |  | | | | | | | | | | | | | | | | | **Japan -** National licensure exists for direct care workers. Licensed care workers, provide ADL support “just like other direct care workers, but the training requirements are more extensive. The license is recognized both in institutions and community settings. What is unique about this licensure requirement is that direct care workers with no formal education can obtain a license through care experience and the licensure exam.” | | | | | | | | | | | | | | | | | | | | | | | | | | | | | | | | | | | | | | | | | | | | | | | | | | | | | ^69^ | | |
|  |  | | | | | | | | | | | | | | | | | **US** - Use of a registry or licensing board for credentialing was significantly related to lower catheter use | | | | | | | | | | | | | | | | | | | | | | | | | | | | | | | | | | | | | | | | | | | | | | | | | | | | | ^71^ | | |
|  |  | | | | | | | | | | | | | | | | | CNA licensure was significantly associated with lower odds of falls | | | | | | | | | | | | | | | | | | | | | | | | | | | | | | | | | | | | | | | | | | | | | | | | | | | | |  |  |  |
| **Qualifications**  **%** | **National qualification** | | | | | | | | | **Permanent FT** | | | | | | | | | | | | | | **Temporary/Casual FT** | | | | | | | | | | | | | | | | | | | | | | | | | | | | | | **Temporary/Casual PT** | | | | | | | | | | | | | | | | |  | | |
|  | With | | | | | | | | | 50.7 | | | | | | | | | | | | | | 29.5 | | | | | | | | | | | | | | | | | | | | | | | | | | | | | | 16.5 | | | | | | | | | | | | | | | | | ^67^ | | |
|  | Without | | | | | | | | | 49.3 | | | | | | | | | | | | | | 70.5 | | | | | | | | | | | | | | | | | | | | | | | | | | | | | | 83.5 | | | | | | | | | | | | | | | | |  | | |
|  | Intermediate care facility with % of qualified SW | | | | | | | | **0-25%** | | | | | | | | | | | | | | **26-50%** | | | | | | | | | | | | | | | | | | | | | | **51-75%** | | | | | | | | | | | | | | | | | | | **76-100%** | | | | | | |  | | |
|  |  |  |  |  |  |  |  |  | 30 | | | | | | | | | | | | | | 50 | | | | | | | | | | | | | | | | | | | | | | 7 | | | | | | | | | | | | | | | | | | | 13 | | | | | | | ^18^ | | |
|  | 24% | | | | | | | | Of TAFE certified DCWs with no high school qualifications | | | | | | | | | | | | | | | | | | | | | | | | | | | | | | | | | | | | | | | | | | | | | | | | | | | | | | | | | | | | | | ^15^ | | |
|  | ↓from 17-10% | | | | | | | | Decline since 2003 in proportion of personal care workers with nursing qualifications and who appear overqualified for their jobs | | | | | | | | | | | | | | | | | | | | | | | | | | | | | | | | | | | | | | | | | | | | | | | | | | | | | | | | | | | | | |  | | |
|  | 5.4% | | | | | | | | Proportion of PCAs with certification | | | | | | | | | | | | | | | | | | | | | | | | | | | | | | | | | | | | | | | | | | | | | | | | | | | | | | | | | | | | | | ^73^ | | |
|  |  | | | | | | | | Significant majority of non-nursing care staff for older people in Ireland do not possess the minimum qualification as laid down in national standards. | | | | | | | | | | | | | | | | | | | | | | | | | | | | | | | | | | | | | | | | | | | | | | | | | | | | | | | | | | | | | | ^20^ | | |
|  |  | | | | | | | | Of the staff that do hold a qualification, vast majority hold the minimum qualification and few either hold or are pursuing a higher level of qualification. | | | | | | | | | | | | | | | | | | | | | | | | | | | | | | | | | | | | | | | | | | | | | | | | | | | | | | | | | | | | | |  | | |
|  |  | | | | | | | | Program requirements vary from province to province – difficult to transfer | | | | | | | | | | | | | | | | | | | | | | | | | | | | | | | | | | | | | | | | | | | | | | | | | | | | | | | | | | | | | | ^44^ | | |
|  |  | | | | | | | | NVQs require the candidate to be assessed in his/her workplace, and some degree of knowledge and skill needs to be in place before commencement of qualification. | | | | | | | | | | | | | | | | | | | | | | | | | | | | | | | | | | | | | | | | | | | | | | | | | | | | | | | | | | | | | | ^47^ | | |
|  |  | | | | | | | | The Department of Health, in association with Skills for Health, has developed new qualifications for people working in the NHS. | | | | | | | | | | | | | | | | | | | | | | | | | | | | | | | | | | | | | | | | | | | | | | | | | | | | | | | | | | | | | | ^45^ | | |
|  | **Overseas qualifications** | | | | | | | | Formal qualifications to work in direct care are not required, however high proportions of “workers obtain relevant qualifications through the Technical and Further Education (TAFE) System” | | | | | | | | | | | | | | | | | | | | | | | | | | | | | | | | | | | | | | | | | | | | | | | | | | | | | | | | | | | | | | ^15^ | | |
|  |  | | | | | | | | Bridging courses enabling oversea workers to qualify for LTC | | | | | | | | | | | | | | | | | | | | | | | | | | | | | | | | | | | | | | | | | | | | | | | | | | | | | | | | | | | | | |  |  |  |
| **Program Specific Information** | **National Vocational Qualification (NVQ)** | | | | | | | | **Completed NVQ** | | | | | | | | | | | | | | | **2** | | | | | | | | | | | **3** | | | | | | | | | | | | | | | | | | **4** | | | | | | | | | **Other (not stated)** | | | | | | | | | ^18^ | | |
|  |  |  |  |  |  |  |  |  | 39 | | | | | | | | | | | | | | | 8 | | | | | | | | | | | 23 | | | | | | | | | | | | | | | | | | <1 | | | | | | | | | 8 | | | | | | | | |  |  |  |
|  |  |  |  |  |  |  |  |  | Estimate that by 2005 50% will have NVQ of at least level 2 in home care sector | | | | | | | | | | | | | | | | | | | | | | | | | | | | | | | | | | | | | | | | | | | | | | | | | | | | | | | | | | | | | | ^42^ | | |
|  |  |  |  |  |  |  |  |  | UK: Multiple level process of NVQ qualifications, with higher number levels involving higher competence | | | | | | | | | | | | | | | | | | | | | | | | | | | | | | | | | | | | | | | | | | | | | | | | | | | | | | | | | | | | | | ^47^ | | |
|  |  |  |  |  |  |  |  |  | Attainment of level 3 NVQ results in HCAs performing at a greater level in the provision of physical and psychosocial care than newly qualified nurses | | | | | | | | | | | | | | | | | | | | | | | | | | | | | | | | | | | | | | | | | | | | | | | | | | | | | | | | | | | | | |  |  |  |
|  |  |  |  |  |  |  |  |  | No specific time frames or criteria for when NVQ can be commenced. HCAs have experienced difficulty in accessing NVQs due to manager resistance and lack of funding; therefore, by the time a HCA commences an NVQ he/she may have already been in the role for some time | | | | | | | | | | | | | | | | | | | | | | | | | | | | | | | | | | | | | | | | | | | | | | | | | | | | | | | | | | | | | |  |  |  |
|  | **Home Helper** | | | | | | | | 3 level process for Home Helpers in Japan with Home Helper 3 being the most basic/entry-level  Home Helper 3: part-time or as “registered” worker  Home Helper 2: work full-time or close to full-time  Home Helper 1: home care team leaders/managers | | | | | | | | | | | | | | | | | | | | | | | | | | | | | | | | | | | | | | | | | | | | | | | | | | | | | | | | | | | | | | ^69^ | | |
|  | **Dementia Training Program** | | | | | | | | Form and content problems were prevalent across all curricular types. | | | | | | | | | | | | | | | | | | | | | | | | | | | | | | | | | | | | | | | | | | | | | | | | | | | | | | | | | | | | | | ^43^ | | |
|  |  |  |  |  |  |  |  |  | On initial submission, 90% of curricula submitted did not include learning objectives, time formats or didactic approach | | | | | | | | | | | | | | | | | | | | | | | | | | | | | | | | | | | | | | | | | | | | | | | | | | | | | | | | | | | | | |  |  |  |
|  | **QP** | | | | | | | | Professional training course for nursing auxiliaries | | | | | | | | | | | | | | | | | | | | | | | | | | | | | | | | | | | | | | | | | | | | | | | | | | | | | | | | | | | | | | ^19^ | | |
|  |  |  |  |  |  |  |  |  | 70.6 | | | | | | | Of students expect to learn new techniques and essential procedures for the profession | | | | | | | | | | | | | | | | | | | | | | | | | | | | | | | | | | | | | | | | | | | | | | | | | | | | | | |  |  |  |
|  |  |  |  |  |  |  |  |  | 61.5 | | | | | | | Of students expect a course that can give me knowledge to understand the meaning of the work I am doing | | | | | | | | | | | | | | | | | | | | | | | | | | | | | | | | | | | | | | | | | | | | | | | | | | | | | | |  |  |  |
| **Evaluation of Skill Competency**  % of agencies who assessed skill competency in the following way | Self-report | | | | | | | | | | | | | | | | | | | | | | | | | 58.5 (n=103) | | | | | | | | | | | | | | | | | | | | | | | | | | | | | | | | | | | | | | | | | | | | | ^40^ | | |
|  | Performed test of caregiver skills | | | | | | | | | | | | | | | | | | | | | | | | | 35.2 (n=62) | | | | | | | | | | | | | | | | | | | | | | | | | | | | | | | | | | | | | | | | | | | | |  |  |  |
|  | Client feedback | | | | | | | | | | | | | | | | | | | | | | | | | 35.2 (n=62) | | | | | | | | | | | | | | | | | | | | | | | | | | | | | | | | | | | | | | | | | | | | |  |  |  |
|  | Shadowing or on-site training | | | | | | | | | | | | | | | | | | | | | | | | | 15.3 (n=27) | | | | | | | | | | | | | | | | | | | | | | | | | | | | | | | | | | | | | | | | | | | | |  |  |  |
|  | Asking previous references about skill | | | | | | | | | | | | | | | | | | | | | | | | | 10.8 (n=19) | | | | | | | | | | | | | | | | | | | | | | | | | | | | | | | | | | | | | | | | | | | | |  |  |  |
|  | Giving the employee a manual | | | | | | | | | | | | | | | | | | | | | | | | | 1.1 (n=2) | | | | | | | | | | | | | | | | | | | | | | | | | | | | | | | | | | | | | | | | | | | | |  |  |  |
|  | Amount of time spent training (range) | | | | | | | | | | | | | | | | | | | | | | | | | None – 1 week | | | | | | | | | | | | | | | | | | | | | | | | | | | | | | | | | | | | | | | | | | | | |  |  |  |
| **Recommend** |  | | | Method of training promoted should be competency based training programs which improves self-confidence and self-reported performance. | | | | | | | | | | | | | | | | | | | | | | | | | | | | | | | | | | | | | | | | | | | | | | | | | | | | | | | | | | | | | | | | | | | ^47^ | | |
|  |  |  |  | Overall findings suggest that regulatory modifications could be beneficial to improve resident care outcomes in nursing homes | | | | | | | | | | | | | | | | | | | | | | | | | | | | | | | | | | | | | | | | | | | | | | | | | | | | | | | | | | | | | | | | | | | ^71^ | | |
|  |  |  |  | The study proposes the Florida credentialing program as a model to ensure that accurate and educationally sound curricula are used to train direct care workers. | | | | | | | | | | | | | | | | | | | | | | | | | | | | | | | | | | | | | | | | | | | | | | | | | | | | | | | | | | | | | | | | | | | ^43^ | | |
| **Training**  % | 93 | | | | Providers with training in place | | | | | | | | | | | | | | | | | | | | | | | | | | | | | | | | | | | | | | | | | | | | | | | | | | | | | | | | | | | | | | | | | | ^28^ | | |
|  | 100 | | | | CNA% Received Initial Training before employment in role | | | | | | | | | | | | | | | | | | | | | | | | | | | | | | | | | | | | | | | | | | | | | | | | | | | | | | | | | | | | | | | | | | ^35^ | | |
|  | 83.7 | | | | HHA% Received Initial Training before employment in role | | | | | | | | | | | | | | | | | | | | | | | | | | | | | | | | | | | | | | | | | | | | | | | | | | | | | | | | | | | | | | | | | |  |  |  |
|  | 40 | | | | Facilities that employ HCAs | | | | | | | | | | | | | | | | | | | | | | | | | | | | | | | | | | | | | | | | | | | | | | | | | | | | | | | | | | | | | | | | | | ^29^ | | |
|  |  | | 45.2 | | Facilities employing trained HCAs | | | | | | | | | | | | | | | | | | | | | | | | | | | | | | | | | | | | | | | | | | | | | | | | | | | | | | | | | | | | | | | | | |  |  |  |
|  |  | | 42.9 | | Facilities employing untrained HCAs | | | | | | | | | | | | | | | | | | | | | | | | | | | | | | | | | | | | | | | | | | | | | | | | | | | | | | | | | | | | | | | | | |  |  |  |
|  |  | | 7.1 | | Facilities that employ some trained and some untrained HCAs | | | | | | | | | | | | | | | | | | | | | | | | | | | | | | | | | | | | | | | | | | | | | | | | | | | | | | | | | | | | | | | | | |  | | |
|  | “At least two provinces in Canada report that many of their HSWs do not have formal training” | | | | | | | | | | | | | | | | | | | | | | | | | | | | | | | | | | | | | | | | | | | | | | | | | | | | | | | | | | | | | | | | | | | | | | ^44^ | | |
|  | **Students** | | | | | | | | | | | 62.1 | | | | | | | Work between 25-44 hours/week | | | | | | | | | | | | | | | | | | | | | | | | | | | | | | | | | | | | | | | | | | | | | | | | | | | | ^19^ | | |
|  |  | | | | | | | | | | | 91.7 | | | | | | | Active in general hospitals | | | | | | | | | | | | | | | | | | | | | | | | | | | | | | | | | | | | | | | | | | | | | | | | | | | |  | | |
|  | **Associated Factors** | | | | | | | | | | | 40 | | | | | | | Home-based providers average attendance for training | | | | | | | | | | | | | | | | | | | | | | | | | | | | | | | | | | | | | | | | | | | | | | | | | | | | ^28^ | | |
|  |  |  |  |  |  |  |  |  |  |  |  |  | | | | | | | Combination of increased initial training and annual CE hours was significantly associated with NH reporting lower antidepressant and antipsychotic use and lower average medication use. | | | | | | | | | | | | | | | | | | | | | | | | | | | | | | | | | | | | | | | | | | | | | | | | | | | | ^71^ | | |
|  |  |  |  |  |  |  |  |  |  |  |  |  | | | | | | | 26 states required CNAs to have more initial training hours than the federal requirement of 75 hours | | | | | | | | | | | | | | | | | | | | | | | | | | | | | | | | | | | | | | | | | | | | | | | | | | | |  | | |
|  |  | | | | | | | | | | |  | | | | | | | Currently few SW have any formal education | | | | | | | | | | | | | | | | | | | | | | | | | | | | | | | | | | | | | | | | | | | | | | | | | | | | ^42^ | | |
|  |  | | | | | | | | | | |  | | | | | | | “At least two provinces in Canada report that many of their HSWs do not have formal training” | | | | | | | | | | | | | | | | | | | | | | | | | | | | | | | | | | | | | | | | | | | | | | | | | | | | ^44^ | | |
|  | **Barriers to attend Training** | | | | | | | | | | | | Providers believe poor attendance due to absence of incentives, other commitments (family, secondary employment, etc.), and distance from work | | | | | | | | | | | | | | | | | | | | | | | | | | | | | | | | | | | | | | | | | | | | | | | | | | | | | | | | | | ^28^ | | |
|  |  | | | | | | | | | | | | 51.9 | | | | | | Cost of transportation to school | | | | | | | | | | | | | | | | | | | | | | | | | | | | | | | | | | | | | | | | | | | | | | | | | | | | ^19^ | | |
|  |  | | | | | | | | | | | | 36.1 | | | | | | Tiredness because of work | | | | | | | | | | | | | | | | | | | | | | | | | | | | | | | | | | | | | | | | | | | | | | | | | | | |  | | |
|  |  | | | | | | | | | | | | 23.6 | | | | | | Not released by employer to attend | | | | | | | | | | | | | | | | | | | | | | | | | | | | | | | | | | | | | | | | | | | | | | | | | | | |  | | |
|  | **Dropout** | | | | | | | | | | | | 29.0 | | | | | | Dropout Rate | | | | | | | | | | | | | | | | | | | | | | | | | | | | | | | | | | | | | | | | | | | | | | | | | | | | ^36^ | | |
|  |  | Females with a significant history of heavy physical workload, failure to pass the back extension test, and low Mental Health scores were predictive of drop out when a recent history of Low Back Pain was present | | | | | | | | | | | | | | | | | | | | | | | | | | | | | | | | | | | | | | | | | | | | | | | | | | | | | | | | | | | | | | | | | | | | |  | | |
|  |  | Recent Low Back Pain was not an independent risk factor of dropout among female NA students | | | | | | | | | | | | | | | | | | | | | | | | | | | | | | | | | | | | | | | | | | | | | | | | | | | | | | | | | | | | | | | | | | | | |  |  |  |
| **SUPPLY** | | | | | | | | | | | | | | | | | | | | | | | | | | | | | | | | | | | | | | | | | | | | | | | | | | | | | | | | | | | | | | | | | | | | | | | | | |
| **Themes** | **Outcomes** | | | | | | | | | | | | | | | | | | |  | | | | | | | | | | | | | | | | | | | | | | | | | | | | | | | | | | | | | | | | | | | | | | | | | | | **Ref.** | | |
| **Mean Age**  (SD or SE)  * indicates most frequent category (for age category only) | 37.6 (13.83) | | | | | | | | | | | | | | | | | | |  | | | | | | | | | | | | | | | | | | | | | | | | | | | | | | | | | | | | | | | | | | | | | | | | | | | ^77^ | | |
|  | 38.6 (12.61) | | | | | | | | | | | | | | | | | | |  | | | | | | | | | | | | | | | | | | | | | | | | | | | | | | | | | | | | | | | | | | | | | | | | | | | ^51^ | | |
|  | 39 | | | | | | | | | | | | | | | | | | |  | | | | | | | | | | | | | | | | | | | | | | | | | | | | | | | | | | | | | | | | | | | | | | | | | | | ^76^ | | |
|  | 41 | | | | | | | | | | | | | | | | | | |  | | | | | | | | | | | | | | | | | | | | | | | | | | | | | | | | | | | | | | | | | | | | | | | | | | | ^22^ | | |
|  | 41.9(8.2) | | | | | | | | | | | | | | | | | | |  | | | | | | | | | | | | | | | | | | | | | | | | | | | | | | | | | | | | | | | | | | | | | | | | | | | ^66^ | | |
|  | 42.83 (13. 58) | | | | | | | | | | | | | | | | | | |  | | | | | | | | | | | | | | | | | | | | | | | | | | | | | | | | | | | | | | | | | | | | | | | | | | | ^79^ | | |
|  | 45 (medication aides) | | | | | | | | | | | | | | | | | | |  | | | | | | | | | | | | | | | | | | | | | | | | | | | | | | | | | | | | | | | | | | | | | | | | | | | ^14^ | | |
|  | 47.6 (9.08) | | | | | | | | | | | | | | | | | | |  | | | | | | | | | | | | | | | | | | | | | | | | | | | | | | | | | | | | | | | | | | | | | | | | | | | ^32^ | | |
|  | 40-49* (long-term care) | | | | | | | | | | | | | | | | | | |  | | | | | | | | | | | | | | | | | | | | | | | | | | | | | | | | | | | | | | | | | | | | | | | | | | | ^62^ | | |
|  | 40-49 (mode) | | | | | | | | | | | | | | | | | | |  | | | | | | | | | | | | | | | | | | | | | | | | | | | | | | | | | | | | | | | | | | | | | | | | | | | ^20^ | | |
|  | 31-50 (highest %, students) | | | | | | | | | | | | | | | | | | |  | | | | | | | | | | | | | | | | | | | | | | | | | | | | | | | | | | | | | | | | | | | | | | | | | | | ^19^ | | |
|  | **By Setting** | | | | | | | | | | | | | **H** | | | | | | | | | | | | | | | | | | | **NH** | | | | | | | | | | | | | | | | | | | | | | | | | | | | **HH** | | | | | | | | | |  | | |
|  |  | | | | | | | | | | | | | 36.3 | | | | | | | | | | | | | | | | | | | 36.6 | | | | | | | | | | | | | | | | | | | | | | | | | | | | 46.7 | | | | | | | | | | ^81^ | | |
|  | ** | | | | | | | | | | | | | 38.0 | | | | | | | | | | | | | | | | | | | 36.4 | | | | | | | | | | | | | | | | | | | | | | | | | | | | 42.8 | | | | | | | | | | ^39^ | | |
|  |  | | | | | | | | | | | | | 40.5 | | | | | | | | | | | | | | | | | | | 38 | | | | | | | | | | | | | | | | | | | | | | | | | | | | 46.2 | | | | | | | | | | ^17^ | | |
|  |  | | | | | | | | | | | | | 40 | | | | | | | | | | | | | | | | | | | 38 | | | | | | | | | | | | | | | | | | | | | | | | | | | | 45 | | | | | | | | | | ^22^ | | |
|  |  | | | | | | | | | | | | | 36 (11) | | | | | | | | | | | | | | | | | | |  | | | | | | | | | | | | | | | | | | | | | | | | | | | |  | | | | | | | | | | ^78^ | | |
|  |  | | | | | | | | | | | | |  | | | | | | | | | | | | | | | | | | | 31.2 (8.5) | | | | | | | | | | | | | | | | | | | | | | | | | | | |  | | | | | | | | | | ^80^ | | |
|  |  | | | | | | | | | | | | |  | | | | | | | | | | | | | | | | | | | 37.7 (11.8) | | | | | | | | | | | | | | | | | | | | | | | | | | | | ^66^ | | | | | | | | | |  | | |
|  |  | | | | | | | | | | | | |  | | | | | | | | | | | | | | | | | | | 38.2 (0.40) | | | | | | | | | | | | | | | | | | | | | | | | | | | |  | | | | | | | | | | ^49^ | | |
|  |  | | | | | | | | | | | | |  | | | | | | | | | | | | | | | | | | | 38.52 (0.34) | | | | | | | | | | | | | | | | | | | | | | | | | | | |  | | | | | | | | | | ^24^ | | |
|  |  | | | | | | | | | | | | |  | | | | | | | | | | | | | | | | | | | 38.7 (0.3) | | | | | | | | | | | | | | | | | | | | | | | | | | | |  | | | | | | | | | | ^54^ | | |
|  |  | | | | | | | | | | | | |  | | | | | | | | | | | | | | | | | | | 39.39 (12.96) | | | | | | | | | | | | | | | | | | | | | | | | | | | | 46.56 (13.33) | | | | | | | | | | ^79^ | | |
|  |  | | | | | | | | | | | | |  | | | | | | | | | | | | | | | | | | |  | | | | | | | | | | | | | | | | | | | | | | | | | | | | 43.6 (10.77) | | | | | | | | | | ^27^ | | |
|  |  | | | | | | | | | | | | |  | | | | | | | | | | | | | | | | | | |  | | | | | | | | | | | | | | | | | | | | | | | | | | | | 44 | | | | | | | | | | ^41^ | | |
|  |  | | | | | | | | | | | | |  | | | | | | | | | | | | | | | | | | |  | | | | | | | | | | | | | | | | | | | | | | | | | | | | 45.5 (13.6) | | | | | | | | | | ^73^ | | |
|  |  | | | | | | | | | | | | |  | | | | | | | | | | | | | | | | | | | 18-30* and 31-45* | | | | | | | | | | | | | | | | | | | | | | | | | | | |  | | | | | | | | | | ^50^ | | |
|  |  | | | | | | | | | | | | |  | | | | | | | | | | | | | | | | | | | 31-35 * | | | | | | | | | | | | | | | | | | | | | | | | | | | |  | | | | | | | | | | ^55^ | | |
|  |  | | | | | | | | | | | | |  | | | | | | | | | | | | | | | | | | | 30-44 * | | | | | | | | | | | | | | | | | | | | | | | | | | | |  | | | | | | | | | | ^53^ | | |
|  |  | | | | | | | | | | | | |  | | | | | | | | | | | | | | | | | | | 41-50 * | | | | | | | | | | | | | | | | | | | | | | | | | | | |  | | | | | | | | | | ^75^ | | |
|  |  | | | | | | | | | | | | |  | | | | | | | | | | | | | | | | | | | 30-45 * | | | | | | | | | | | | | | | | | | | | | | | | | | | |  | | | | | | | | | | ^56^ | | |
|  |  | | | | | | | | | | | | |  | | | | | | | | | | | | | | | | | | | ≥45 (tenure >1 year) | | | | | | | | | | | | | | | | | | | | | | | | | | | |  | | | | | | | | | |  | | |
|  |  | | | | | | | | | | | | |  | | | | | | | | | | | | | | | | | | | **NH** | | | | | | | | | | | | | | | | | | | | | | | | | | | | **Group home** | | | | | | | | | |  | | |
|  |  | | | | | | | | | | | | |  | | | | | | | | | | | | | | | | | | | 37.3 (11.7) | | | | | | | | | | | | | | | | | | | | | | | | | | | | 38.0 (11.7) | | | | | | | | | | ^68^ | | |
|  |  | | | | | | | | | | | | |  | | | | | | | | | | | | | | | | | | | **Assisted living** | | | | | | | | | | | | | | | | | | | | | | | | | | | | **Adult day service** | | | | | | | | | |  | | |
|  |  | | | | | | | | | | | | |  | | | | | | | | | | | | | | | | | | | 41.44 (13.19) | | | | | | | | | | | | | | | | | | | | | | | | | | | | 44.39 (13.69) | | | | | | | | | | ^79^ | | |
|  |  | | | | | | | | | | | | |  | | | | | | | | | | | | | | | | | | | **Geriatric inter-med. facility** | | | | | | | | | | | | | | | | | | | | | | | | | | | | | | | | | | | | | |  | | |
|  |  | | | | | | | | | | | | |  | | | | | | | | | | | | | | | | | | | 38.5 (12.1) | | | | | | | | | | | | | | | | | | | | | | | | | | | |  | | | | | | | | | | ^68^ | | |
|  | **By Location** ( NH, Significant across all (p=0.02)) | | | | | | | | | | | | | | | | | | | | | | | | | | | | | | | | | | | | | | | | | | | | | | | | | | | | | | | | | | | | | | | | | | | | | |  | | |
|  |  | | | | | | | | | | | | | **Urban** | | | | | | | | | | | | | | | | | | | **Micropolitan** | | | | | | | | | | | | | | | | | | | | | | | | | | | | **Other Rural** | | | | | | | | | |  | | |
|  |  | | | | | | | | | | | | | 30-44 * | | | | | | | | | | | | | | | | | | | < 30 * | | | | | | | | | | | | | | | | | | | | | | | | | | | | 30-44 * | | | | | | | | | | ^53^ | | |
|  | **By Immigration Status** ( NH, Significant across all (p<0.05)) | | | | | | | | | | | | | | | | | | | | | | | | | | | | | | | | | | | | | | | | | | | | | | | | | | | | | | | | | | | | | | | | | | | | | |  | | |
|  |  | | | | | | | | | | | | |  | | | | | | | | | | | | | | | | | | | **Immigrant** | | | | | | | | | | | | | | | | | | | | | | | | | | | | **Non-immigrant** | | | | | | | | | |  | | |
|  |  | | | | | | | | | | | | |  | | | | | | | | | | | | | | | | | | | 30-44 * | | | | | | | | | | | | | | | | | | | | | | | | | | | | ≥45 * | | | | | | | | | | ^33^ | | |
|  | **By Gender** | | | | | | | | | | | | |  | | | | | | | | | | | | | | | | | | | **Female** | | | | | | | | | | | | | | | | | | | | | | | | | | | | **Male** | | | | | | | | | |  | | |
|  |  | | | | | | | | | | | | |  | | | | | | | | | | | | | | | | | | | 36.69 (12.7) | | | | | | | | | | | | | | | | | | | | | | | | | | | | 36.5 (11.8) | | | | | | | | | | ^52^ | | |
|  |  | | | | | | | | | | | | |  | | | | | | | | | | | | | | | | | | | 41-50 * | | | | | | | | | | | | | | | | | | | | | | | | | | | | 31-40 * | | | | | | | | | | ^28^ | | |
|  | **By Job Status** | | | | | | | | | | | | | **Permanent FT** | | | | | | | | | | | | | | | | | | | **Temporary/Casual FT** | | | | | | | | | | | | | | | | | | | | | | | | | | | | **Temporary/Casual PT** | | | | | | | | | |  | | |
|  |  | | | | | | | | | | | | | 18-29 * | | | | | | | | | | | | | | | | | | | 50-75 * | | | | | | | | | | | | | | | | | | | | | | | | | | | | 50-75* | | | | | | | | | | ^67^ | | |
|  |  | | | | | | | | | | | | |  | | | | | | | | | | | | | | | | | | | **Stayers** | | | | | | | | | | | | | | | | | | | | | | | | | | | | **Leavers** | | | | | | | | | | ^32^ | | |
|  |  | | | | | | | | | | | | |  | | | | | | | | | | | | | | | | | | | 41-50 * | | | | | | | | | | | | | | | | | | | | | | | | | | | | 51-60 * | | | | | | | | | |  | | |
|  |  | | | | | | | | | | | | |  | | | | | | | | | | | | | | | | | | | **CNA** | | | | | | | | | | | | | | | | | | | | | | | | | | | | **HHA** | | | | | | | | | |  | | |
|  |  | | | | | | | | | | | | |  | | | | | | | | | | | | | | | | | | | 38.7 | | | | | | | | | | | | | | | | | | | | | | | | | | | | 45.8** | | | | | | | | | | ^35^ | | |
| **Education** | **DCWs Overall** | | | | | | | | | | | | |  | | | | | | | | | | | | | | | | | | |  | | | | | | | | | | | | | | | | | | | | | | | | | | | |  | | | | | | | | | |  | | |
| % With High School Completion or Less  (SD or SE) | >90 | | | | | | | | | | | | |  | | | | | | | | | | | | | | | | | | |  | | | | | | | | | | | | | | | | | | | | | | | | | | | |  | | | | | | | | | | ^34^ | | |
|  | 75.32 | | | | | | | | | | | | |  | | | | | | | | | | | | | | | | | | |  | | | | | | | | | | | | | | | | | | | | | | | | | | | |  | | | | | | | | | | ^49^ | | |
|  | 67 | | | | | | | | | | | | |  | | | | | | | | | | | | | | | | | | |  | | | | | | | | | | | | | | | | | | | | | | | | | | | |  | | | | | | | | | | ^76^ | | |
|  | 62 | | | | | | | | | | | | |  | | | | | | | | | | | | | | | | | | |  | | | | | | | | | | | | | | | | | | | | | | | | | | | |  | | | | | | | | | | ^22^ | | |
|  | 55 | | | | | | | | | | | | |  | | | | | | | | | | | | | | | | | | |  | | | | | | | | | | | | | | | | | | | | | | | | | | | |  | | | | | | | | | | ^77^ | | |
|  | **By Setting** | | | | | | | | | | | | | **H** | | | | | | | | | | | | | | | | | | | **NH** | | | | | | | | | | | | | | | | | | | | | | | | | | | | **HH** | | | | | | | | | |  | | |
|  |  | | | | | | | | | | | | | 59.7 | | | | | | | | | | | | | | | | | | | 83.5 | | | | | | | | | | | | | | | | | | | | | | | | | | | | 69.4 | | | | | | | | | | ^81^ | | |
|  |  | | | | | | | | | | | | | 52.5 | | | | | | | | | | | | | | | | | | | 72.5 | | | | | | | | | | | | | | | | | | | | | | | | | | | | 70.9 | | | | | | | | | | ^39^ | | |
|  |  | | | | | | | | | | | | | 51 | | | | | | | | | | | | | | | | | | | 65 | | | | | | | | | | | | | | | | | | | | | | | | | | | | 64 | | | | | | | | | | ^22^ | | |
|  |  | | | | | | | | | | | | |  | | | | | | | | | | | | | | | | | | | 92 | | | | | | | | | | | | | | | | | | | | | | | | | | | |  | | | | | | | | | | ^80^ | | |
|  |  | | | | | | | | | | | | |  | | | | | | | | | | | | | | | | | | | 75.1 | | | | | | | | | | | | | | | | | | | | | | | | | | | |  | | | | | | | | | | ^53^ | | |
|  |  | | | | | | | | | | | | |  | | | | | | | | | | | | | | | | | | | 75 | | | | | | | | | | | | | | | | | | | | | | | | | | | |  | | | | | | | | | | ^50^ | | |
|  |  | | | | | | | | | | | | |  | | | | | | | | | | | | | | | | | | | 75 | | | | | | | | | | | | | | | | | | | | | | | | | | | |  | | | | | | | | | | ^55^ | | |
|  |  | | | | | | | | | | | | |  | | | | | | | | | | | | | | | | | | | 74.7 | | | | | | | | | | | | | | | | | | | | | | | | | | | |  | | | | | | | | | | ^54^ | | |
|  |  | | | | | | | | | | | | |  | | | | | | | | | | | | | | | | | | | 74 | | | | | | | | | | | | | | | | | | | | | | | | | | | |  | | | | | | | | | | ^24^ | | |
|  |  | | | | | | | | | | | | |  | | | | | | | | | | | | | | | | | | |  | | | | | | | | | | | | | | | | | | | | | | | | | | | |  | | | | | | | | | | ^56^ | | |
|  |  | | | | | | | | | | | | |  | | | | | | | | | | | | | | | | | | | 74 (tenure >1yr) | | | | | | | | | | | | | | | | | | | | | | | | | | | |  | | | | | | | | | |  | | |
|  |  | | | | | | | | | | | | |  | | | | | | | | | | | | | | | | | | | 68.9 (1.2) | | | | | | | | | | | | | | | | | | | | | | | | | | | |  | | | | | | | | | | ^21^ | | |
|  |  | | | | | | | | | | | | |  | | | | | | | | | | | | | | | | | | | 67.4(treatment) | | | | | | | | | | | | | | | | | | | | | | | | | | | |  | | | | | | | | | | ^75^ | | |
|  |  | | | | | | | | | | | | |  | | | | | | | | | | | | | | | | | | | 65.8 (control) | | | | | | | | | | | | | | | | | | | | | | | | | | | |  | | | | | | | | | |  | | |
|  |  | | | | | | | | | | | | |  | | | | | | | | | | | | | | | | | | |  | | | | | | | | | | | | | | | | | | | | | | | | | | | | 72.3 | | | | | | | | | | ^27^ | | |
|  |  | | | | | | | | | | | | |  | | | | | | | | | | | | | | | | | | |  | | | | | | | | | | | | | | | | | | | | | | | | | | | | 40.4 | | | | | | | | | | ^41^ | | |
|  |  | | | | | | | | | | | | |  | | | | | | | | | | | | | | | | | | | **NH** | | | | | | | | | | | | | | | | | | | | | | | | | | | | **Group Home** | | | | | | | | | |  | | |
|  |  | | | | | | | | | | | | |  | | | | | | | | | | | | | | | | | | | 39.8 | | | | | | | | | | | | | | | | | | | | | | | | | | | | 45.1 | | | | | | | | | | ^68^ | | |
|  |  | | | | | | | | | | | | |  | | | | | | | | | | | | | | | | | | | **Geriatric inter-med. facility** | | | | | | | | | | | | | | | | | | | | | | | | | | | | | | | | | | | | | |  | | |
|  |  | | | | | | | | | | | | |  | | | | | | | | | | | | | | | | | | | 46.3 | | | | | | | | | | | | | | | | | | | | | | | | | | | |  | | | | | | | | | | ^68^ | | |
|  |  | | | | | | | | | | | | | Typical education needed for entry: Less than high school for PCA & HCA | | | | | | | | | | | | | | | | | | | | | | | | | | | | | | | | | | | | | | | | | | | | | | | | | | | | | | | | | ^74^ | | |
|  | **By Location** | | | | | | | | | | | | | **H** | | | | | | | | | | | | | | | | | | | **NH** | | | | | | | | | | | | | | | | | | | | | | | | | | | | **HH** | | | | | | | | | |  | | |
|  |  | | | | | | | | | | | | | 74 | | | | | | | | | | | | | | | | | | | 78.4 | | | | | | | | | | | | | | | | | | | | | | | | | | | | 78.5 | | | | | | | | | | ^53^ | | |
|  | **By Immigration Status (NH)** | | | | | | | | | | | | | **Immigrant** | | | | | | | | | | | | | | | | | | | **Non-immigrant** | | | | | | | | | | | | | | | | | | | | | | | | | | | |  | | | | | | | | | |  | | |
|  |  |  |  |  |  |  |  |  |  |  |  |  |  |  | | | | | | | | | | | | | | | | | | | 60.2 | | | | | | | | | | | | | | | | | | | | | | | | | | | | 79 | | | | | | | | | | ^33^ | | |
|  | **By Gender** | | | | | | | | | | | | |  | | | | | | | | | | | | | | | | | | | **Female** | | | | | | | | | | | | | | | | | | | | | | | | | | | | **Male** | | | | | | | | | |  | | |
|  |  | | | | | | | | | | | | |  | | | | | | | | | | | | | | | | | | | 86.3 | | | | | | | | | | | | | | | | | | | | | | | | | | | | 94 | | | | | | | | | | ^52^ | | |
|  | **By Job Status** | | | | | | | | | | | | |  | | | | | | | | | | | | | | | | | | | **Stayers** | | | | | | | | | | | | | | | | | | | | | | | | | | | | **Leavers** | | | | | | | | | |  | | |
|  |  | | | | | | | | | | | | |  | | | | | | | | | | | | | | | | | | | 77.9 | | | | | | | | | | | | | | | | | | | | | | | | | | | | 78.4 | | | | | | | | | | ^32^ | | |
|  |  | | | | | | | | | | | | |  | | | | | | | | | | | | | | | | | | | **CNA** | | | | | | | | | | | | | | | | | | | | | | | | | | | | **HHA** | | | | | | | | | |  | | |
|  |  | | | | | | | | | | | | |  | | | | | | | | | | | | | | | | | | | 74.8 | | | | | | | | | | | | | | | | | | | | | | | | | | | | 60.3 | | | | | | | | | | ^35^ | | |
| % With Some College  (SD or SE) | **DCWs Overall** | | | | | | | | | | | | |  | | | | | | | | | | | | | | | | | | |  | | | | | | | | | | | | | | | | | | | | | | | | | | | |  | | | | | | | | | |  | | |
|  |  | | | | | | | | | | | | | 36.1 | | | | | | | | | | | | | | | | | | |  | | | | | | | | | | | | | | | | | | | | | | | | | | | |  | | | | | | | | | | ^77^ | | |
|  |  | | | | | | | | | | | | | 33 | | | | | | | | | | | | | | | | | | |  | | | | | | | | | | | | | | | | | | | | | | | | | | | |  | | | | | | | | | | ^76^ | | |
|  |  | | | | | | | | | | | | | 23 | | | | | | | | | | | | | | | | | | |  | | | | | | | | | | | | | | | | | | | | | | | | | | | |  | | | | | | | | | | ^22^ | | |
|  |  | | | | | | | | | | | | | 16.8 (37.3) | | | | | | | | | | | | | | | | | | |  | | | | | | | | | | | | | | | | | | | | | | | | | | | |  | | | | | | | | | | ^72^ | | |
|  |  | | | | | | | | | | | | | 8 | | | | | | | | | | | | | | | | | | |  | | | | | | | | | | | | | | | | | | | | | | | | | | | |  | | | | | | | | | | ^79^ | | |
|  | **By Setting** | | | | | | | | | | | | | **H** | | | | | | | | | | | | | | | | | | | **NH** | | | | | | | | | | | | | | | | | | | | | | | | | | | | **HH** | | | | | | | | | |  | | |
|  |  | | | | | | | | | | | | | 38.7 | | | | | | | | | | | | | | | | | | | 28.6 | | | | | | | | | | | | | | | | | | | | | | | | | | | | 34.7 | | | | | | | | | | ^17^ | | |
|  |  | | | | | | | | | | | | | 31 | | | | | | | | | | | | | | | | | | | 24 | | | | | | | | | | | | | | | | | | | | | | | | | | | | 19 | | | | | | | | | | ^22^ | | |
|  |  | | | | | | | | | | | | |  | | | | | | | | | | | | | | | | | | | 8 | | | | | | | | | | | | | | | | | | | | | | | | | | | | 8 | | | | | | | | | | ^79^ | | |
|  |  | | | | | | | | | | | | |  | | | | | | | | | | | | | | | | | | | 26.8 (treatment) | | | | | | | | | | | | | | | | | | | | | | | | | | | |  | | | | | | | | | | ^75^ | | |
|  |  | | | | | | | | | | | | |  | | | | | | | | | | | | | | | | | | | 26.6 (control) | | | | | | | | | | | | | | | | | | | | | | | | | | | |  | | | | | | | | | |  | | |
|  |  | | | | | | | | | | | | |  | | | | | | | | | | | | | | | | | | | 24.9 | | | | | | | | | | | | | | | | | | | | | | | | | | | |  | | | | | | | | | | ^53^ | | |
|  |  | | | | | | | | | | | | |  | | | | | | | | | | | | | | | | | | | 19.4 | | | | | | | | | | | | | | | | | | | | | | | | | | | |  | | | | | | | | | | ^24^ | | |
|  |  | | | | | | | | | | | | |  | | | | | | | | | | | | | | | | | | |  | | | | | | | | | | | | | | | | | | | | | | | | | | | | 8.8 | | | | | | | | | | ^41^ | | |
|  |  | | | | | | | | | | | | |  | | | | | | | | | | | | | | | | | | | **Assisted Living** | | | | | | | | | | | | | | | | | | | | | | | | | | | | **Adult day services** | | | | | | | | | |  | | |
|  |  | | | | | | | | | | | | |  | | | | | | | | | | | | | | | | | | | 8 | | | | | | | | | | | | | | | | | | | | | | | | | | | | 24 | | | | | | | | | | ^79^ | | |
|  | **By Location (NH)** | | | | | | | | | | | | | **Urban** | | | | | | | | | | | | | | | | | | | **Micropolitan** | | | | | | | | | | | | | | | | | | | | | | | | | | | | **Other Rural** | | | | | | | | | |  | | |
|  |  | | | | | | | | | | | | | 26.0 | | | | | | | | | | | | | | | | | | | 21.6 | | | | | | | | | | | | | | | | | | | | | | | | | | | | 21.5 | | | | | | | | | | ^53^ | | |
|  | **By Job Status** | | | | | | | | | | | | |  | | | | | | | | | | | | | | | | | | | **CNA** | | | | | | | | | | | | | | | | | | | | | | | | | | | | **HHA** | | | | | | | | | |  | | |
|  |  | | | | | | | | | | | | |  | | | | | | | | | | | | | | | | | | | 25.2 | | | | | | | | | | | | | | | | | | | | | | | | | | | | 39.7 | | | | | | | | | | ^35^ | | |
| % With Completed College or University Associate or Bachelor’s Degree  (SD or SE) | **DCWs Overall** | | | | | | | | | | | | |  | | | | | | | | | | | | | | | | | | |  | | | | | | | | | | | | | | | | | | | | | | | | | | | |  | | | | | | | | | |  | | |
|  |  | | | | | | | | | | | | | 36.5 | | | | | | | | | | | | | | | | | | |  | | | | | | | | | | | | | | | | | | | | | | | | | | | |  | | | | | | | | | | ^72^ | | |
|  |  | | | | | | | | | | | | | 15 | | | | | | | | | | | | | | | | | | |  | | | | | | | | | | | | | | | | | | | | | | | | | | | |  | | | | | | | | | | ^22^ | | |
|  |  | | | | | | | | | | | | | 8.9 | | | | | | | | | | | | | | | | | | |  | | | | | | | | | | | | | | | | | | | | | | | | | | | |  | | | | | | | | | | ^77^ | | |
|  | **By Setting** | | | | | | | | | | | | | **H** | | | | | | | | | | | | | | | | | | | **NH** | | | | | | | | | | | | | | | | | | | | | | | | | | | | **HH** | | | | | | | | | |  | | |
|  |  | | | | | | | | | | | | | 17 | | | | | | | | | | | | | | | | | | | 12 | | | | | | | | | | | | | | | | | | | | | | | | | | | | 17 | | | | | | | | | | ^22^ | | |
|  |  | | | | | | | | | | | | |  | | | | | | | | | | | | | | | | | | | 5.8 (treatment) | | | | | | | | | | | | | | | | | | | | | | | | | | | |  | | | | | | | | | | ^75^ | | |
|  |  | | | | | | | | | | | | |  | | | | | | | | | | | | | | | | | | | 7.6 (control) | | | | | | | | | | | | | | | | | | | | | | | | | | | |  | | | | | | | | | |  | | |
|  |  | | | | | | | | | | | | |  | | | | | | | | | | | | | | | | | | |  | | | | | | | | | | | | | | | | | | | | | | | | | | | | 50.9 | | | | | | | | | | ^41^ | | |
|  | **By Job Status** | | | | | | | | | | | | |  | | | | | | | | | | | | | | | | | | | **Stayers** | | | | | | | | | | | | | | | | | | | | | | | | | | | | **Leavers** | | | | | | | | | |  | | |
|  |  | | | | | | | | | | | | |  | | | | | | | | | | | | | | | | | | | 22.2 | | | | | | | | | | | | | | | | | | | | | | | | | | | | 21.7 | | | | | | | | | | ^32^ | | |
| Current Level of Education (SD or SE) | **NA Scores on Test of Basic Literacy (NAs) N=88** | | | | | | | | | | | | | | | | | | | | | | | | | | | | | | | | | | | | **Grade** | | | | | | | | | | | | | | | | | | | **Score** | | | | | | | | | | | | | **%** | | ^78^ | | |
|  |  | | | | | | | | | | | | | | | | | | | | | | | | | | | | | | | | | | | | 8.0-8.9 | | | | | | | | | | | | | | | | | | | 37-40 | | | | | | | | | | | | | 40 | |  | | |
|  |  | | | | | | | | | | | | | | | | | | | | | | | | | | | | | | | | | | | | 7.0-7.9 | | | | | | | | | | | | | | | | | | | 36 | | | | | | | | | | | | | 8 | |  | | |
|  |  | | | | | | | | | | | | | | | | | | | | | | | | | | | | | | | | | | | | 6.0-6.9 | | | | | | | | | | | | | | | | | | | 34-35 | | | | | | | | | | | | | 17 | |  | | |
|  |  | | | | | | | | | | | | | | | | | | | | | | | | | | | | | | | | | | | | <5.9 | | | | | | | | | | | | | | | | | | | <33 | | | | | | | | | | | | | 35 | |  | | |
|  | **School grade completed** | | | | | | | | | | | | | | | | | | | | | | | | | | | | | | | | | | | | 7.17 (1.56) | | | | | | | | | | | | | | | | | | |  | | | | | | | | | | | | |  | | ^78^ | | |
|  | **Able to use Computers (students)**  **Students finished basic education** | | | | | | | | | | | | | | | | | | | | | | | | | | | | | | | | | | | |  | | | | | | | | | | | | | | | | | | |  | | | | | | | | | | | | | 40.5  69.7 | | ^19^ | | |
| **Marital Status**  % Married/living with a partner (SD or SE) | **DCWs Overall** | | | | | | | | | | | | |  | | | | | | | | | | | | | | | | | | |  | | | | | | | | | | | | | | | | | | | | | | | | | | | |  | | | | | | | | | |  | | |
|  |  | | | | | | | | | | | | | 82.6 | | | | | | | | | | | | | | | | | | |  | | | | | | | | | | | | | | | | | | | | | | | | | | | |  | | | | | | | | | | ^66^ | | |
|  |  | | | | | | | | | | | | | 77.6 | | | | | | | | | | | | | | | | | | |  | | | | | | | | | | | | | | | | | | | | | | | | | | | |  | | | | | | | | | | ^32^ | | |
|  |  | | | | | | | | | | | | | 51.3 | | | | | | | | | | | | | | | | | | |  | | | | | | | | | | | | | | | | | | | | | | | | | | | |  | | | | | | | | | | ^51^ | | |
|  |  | | | | | | | | | | | | | 42 | | | | | | | | | | | | | | | | | | |  | | | | | | | | | | | | | | | | | | | | | | | | | | | |  | | | | | | | | | | ^75^ | | |
|  |  | | | | | | | | | | | | | 38 | | | | | | | | | | | | | | | | | | |  | | | | | | | | | | | | | | | | | | | | | | | | | | | |  | | | | | | | | | | ^22^ | | |
|  |  | | | | | | | | | | | | | 51.5 (students) | | | | | | | | | | | | | | | | | | |  | | | | | | | | | | | | | | | | | | | | | | | | | | | |  | | | | | | | | | | ^19^ | | |
|  | **By Setting** | | | | | | | | | | | | | **H** | | | | | | | | | | | | | | | | | | | **NH** | | | | | | | | | | | | | | | | | | | | | | | | | | | | **HH** | | | | | | | | | |  | | |
|  |  | | | | | | | | | | | | | 47.9 | | | | | | | | | | | | | | | | | | | 46.1 | | | | | | | | | | | | | | | | | | | | | | | | | | | | 39.9 | | | | | | | | | | ^81^ | | |
|  |  | | | | | | | | | | | | | 46.2 | | | | | | | | | | | | | | | | | | | 42.7 | | | | | | | | | | | | | | | | | | | | | | | | | | | | 44.2 | | | | | | | | | | ^17^ | | |
|  |  | | | | | | | | | | | | | 35 | | | | | | | | | | | | | | | | | | | 38 | | | | | | | | | | | | | | | | | | | | | | | | | | | | 39 | | | | | | | | | | ^22^ | | |
|  |  | | | | | | | | | | | | |  | | | | | | | | | | | | | | | | | | | 50.7(1.31) | | | | | | | | | | | | | | | | | | | | | | | | | | | |  | | | | | | | | | | ^24^ | | |
|  |  | | | | | | | | | | | | |  | | | | | | | | | | | | | | | | | | | 51.3 | | | | | | | | | | | | | | | | | | | | | | | | | | | |  | | | | | | | | | | ^21^ | | |
|  |  | | | | | | | | | | | | |  | | | | | | | | | | | | | | | | | | | 51.1 | | | | | | | | | | | | | | | | | | | | | | | | | | | |  | | | | | | | | | | ^33^ | | |
|  |  | | | | | | | | | | | | |  | | | | | | | | | | | | | | | | | | | 52(0.02) | | | | | | | | | | | | | | | | | | | | | | | | | | | |  | | | | | | | | | | ^56^ | | |
|  |  | | | | | | | | | | | | |  | | | | | | | | | | | | | | | | | | | 54(0.02) (tenure>1yr) | | | | | | | | | | | | | | | | | | | | | | | | | | | |  | | | | | | | | | |  | | |
|  | **By Immigration Status (NH)** | | | | | | | | | | | | | | | | | | | | | | | | | | | | | | | | **Immigrant** | | | | | | | | | | | | | | | | | | | | | | | | | | | | **Non-Immigrant** | | | | | | | | | |  | | |
|  |  | | | | | | | | | | | | |  | | | | | | | | | | | | | | | | | | | 61.7 | | | | | | | | | | | | | | | | | | | | | | | | | | | | 48.8 | | | | | | | | | | ^33^ | | |
|  | **By Job Status** | | | | | | | | | | | | | **Permanent (FT)** | | | | | | | | | | | | | | | | | | | **Temporary/Casual FT** | | | | | | | | | | | | | | | | | | | | | | | | | | | | **Temporary/Casual PT** | | | | | | | | | |  | | |
|  |  | | | | | | | | | | | | | 50.7 | | | | | | | | | | | | | | | | | | | 53.9 | | | | | | | | | | | | | | | | | | | | | | | | | | | | 78.6 | | | | | | | | | | ^67^ | | |
|  |  | | | | | | | | | | | | |  | | | | | | | | | | | | | | | | | | | **CNA** | | | | | | | | | | | | | | | | | | | | | | | | | | | | **HHA** | | | | | | | | | |  | | |
|  |  | | | | | | | | | | | | |  | | | | | | | | | | | | | | | | | | | 51.7 | | | | | | | | | | | | | | | | | | | | | | | | | | | | 51 | | | | | | | | | | ^35^ | | |
| % Widowed, divorced, or separated  (SD or SE) | **DCWs Overall** | | | | | | | | | | | | |  | | | | | | | | | | | | | | | | | | |  | | | | | | | | | | | | | | | | | | | | | | | | | | | |  | | | | | | | | | |  | | |
|  |  | | | | | | | | | | | | | 31 | | | | | | | | | | | | | | | | | | |  | | | | | | | | | | | | | | | | | | | | | | | | | | | |  | | | | | | | | | | ^22^ | | |
|  |  | | | | | | | | | | | | | 27 | | | | | | | | | | | | | | | | | | |  | | | | | | | | | | | | | | | | | | | | | | | | | | | |  | | | | | | | | | | ^76^ | | |
|  | **By Setting** | | | | | | | | | | | | | **H** | | | | | | | | | | | | | | | | | | | **NH** | | | | | | | | | | | | | | | | | | | | | | | | | | | | **HH** | | | | | | | | | |  | | |
|  |  | | | | | | | | | | | | | 27 | | | | | | | | | | | | | | | | | | | 27 | | | | | | | | | | | | | | | | | | | | | | | | | | | | 37 | | | | | | | | | | ^22^ | | |
|  |  | | | | | | | | | | | | | 23.8 | | | | | | | | | | | | | | | | | | | 25.1 | | | | | | | | | | | | | | | | | | | | | | | | | | | | 34.2 | | | | | | | | | | ^17^ | | |
|  |  | | | | | | | | | | | | | 22.5 | | | | | | | | | | | | | | | | | | | 25.9 | | | | | | | | | | | | | | | | | | | | | | | | | | | | 47.6 | | | | | | | | | | ^81^ | | |
|  |  | | | | | | | | | | | | |  | | | | | | | | | | | | | | | | | | | 24 (0.02) (tenure>yr) | | | | | | | | | | | | | | | | | | | | | | | | | | | |  | | | | | | | | | | ^56^ | | |
|  |  | | | | | | | | | | | | |  | | | | | | | | | | | | | | | | | | | 22(0.01) | | | | | | | | | | | | | | | | | | | | | | | | | | | |  | | | | | | | | | |  | | |
|  |  | | | | | | | | | | | | |  | | | | | | | | | | | | | | | | | | | 22.4 | | | | | | | | | | | | | | | | | | | | | | | | | | | |  | | | | | | | | | | ^21^ | | |
|  |  | | | | | | | | | | | | |  | | | | | | | | | | | | | | | | | | | 22.3 | | | | | | | | | | | | | | | | | | | | | | | | | | | |  | | | | | | | | | | ^33^ | | |
|  |  | | | | | | | | | | | | |  | | | | | | | | | | | | | | | | | | | 22.15 (1.05) | | | | | | | | | | | | | | | | | | | | | | | | | | | |  | | | | | | | | | | ^24^ | | |
|  | **By Immigration Status (NH)** | | | | | | | | | | | | | | | | | | | | | | | | | | | | | | | | **Immigrant** | | | | | | | | | | | | | | | | | | | | | | | | | | | | **Non-Immigrant** | | | | | | | | | |  | | |
|  |  | | | | | | | | | | | | |  | | | | | | | | | | | | | | | | | | | 19.0 | | | | | | | | | | | | | | | | | | | | | | | | | | | | 23.1 | | | | | | | | | | ^33^ | | |
|  | **By Job Status** | | | | | | | | | | | | | **Permanent FT** | | | | | | | | | | | | | | | | | | | **Temporary/Casual FT** | | | | | | | | | | | | | | | | | | | | | | | | | | | | **Temporary/Casual PT** | | | | | | | | | |  | | |
|  |  | | | | | | | | | | | | | 13 | | | | | | | | | | | | | | | | | | | 15.6 | | | | | | | | | | | | | | | | | | | | | | | | | | | | 12.5 | | | | | | | | | | ^67^ | | |
|  |  | | | | | | | | | | | | |  | | | | | | | | | | | | | | | | | | | **CNA** | | | | | | | | | | | | | | | | | | | | | | | | | | | | **HHA** | | | | | | | | | |  | | |
|  |  | | | | | | | | | | | | |  | | | | | | | | | | | | | | | | | | | 22.3 | | | | | | | | | | | | | | | | | | | | | | | | | | | | 32.2 | | | | | | | | | | ^35^ | | |
| % Single/ Never married  (SD or SE) | **DCWs Overall** | | | | | | | | | | | | | 31 | | | | | | | | | | | | | | | | | | |  | | | | | | | | | | | | | | | | | | | | | | | | | | | |  | | | | | | | | | | ^76^ | | |
|  |  | | | | | | | | | | | | | 31 | | | | | | | | | | | | | | | | | | |  | | | | | | | | | | | | | | | | | | | | | | | | | | | |  | | | | | | | | | | ^22^ | | |
|  |  | | | | | | | | | | | | | 26.22 | | | | | | | | | | | | | | | | | | |  | | | | | | | | | | | | | | | | | | | | | | | | | | | |  | | | | | | | | | | ^51^ | | |
|  | **By Setting** | | | | | | | | | | | | | **H** | | | | | | | | | | | | | | | | | | | **NH** | | | | | | | | | | | | | | | | | | | | | | | | | | | | **HH** | | | | | | | | | |  | | |
|  |  | | | | | | | | | | | | | 38 | | | | | | | | | | | | | | | | | | | 36 | | | | | | | | | | | | | | | | | | | | | | | | | | | | 24 | | | | | | | | | | ^22^ | | |
|  |  | | | | | | | | | | | | | 30.1 | | | | | | | | | | | | | | | | | | | 32.0 | | | | | | | | | | | | | | | | | | | | | | | | | | | | 21.6 | | | | | | | | | | ^17^ | | |
|  |  | | | | | | | | | | | | | 29.6 | | | | | | | | | | | | | | | | | | | 28.0 | | | | | | | | | | | | | | | | | | | | | | | | | | | | 12.5 | | | | | | | | | | ^81^ | | |
|  |  | | | | | | | | | | | | |  | | | | | | | | | | | | | | | | | | | 26.6 | | | | | | | | | | | | | | | | | | | | | | | | | | | |  | | | | | | | | | | ^33^ | | |
|  |  | | | | | | | | | | | | |  | | | | | | | | | | | | | | | | | | | 26.41(1.07) | | | | | | | | | | | | | | | | | | | | | | | | | | | |  | | | | | | | | | | ^24^ | | |
|  |  | | | | | | | | | | | | |  | | | | | | | | | | | | | | | | | | | 26.3 (1.1) | | | | | | | | | | | | | | | | | | | | | | | | | | | |  | | | | | | | | | | ^21^ | | |
|  |  | | | | | | | | | | | | |  | | | | | | | | | | | | | | | | | | | 26 (0.01) | | | | | | | | | | | | | | | | | | | | | | | | | | | |  | | | | | | | | | | ^56^ | | |
|  |  | | | | | | | | | | | | |  | | | | | | | | | | | | | | | | | | | 22 (0.01) (tenure>1yr) | | | | | | | | | | | | | | | | | | | | | | | | | | | |  | | | | | | | | | |  | | |
|  | **By Immigration Status (NH)** | | | | | | | | | | | | | | | | | | | | | | | | | | | | | | | | **Immigrant** | | | | | | | | | | | | | | | | | | | | | | | | | | | | **Non-Immigrant** | | | | | | | | | | ^33^ | | |
|  |  | | | | | | | | | | | | |  | | | | | | | | | | | | | | | | | | | 19.4 | | | | | | | | | | | | | | | | | | | | | | | | | | | | 28.1 | | | | | | | | | |  | | |
|  | **By Job Status** | | | | | | | | | | | | | **Permanent FT** | | | | | | | | | | | | | | | | | | | **Temporary/Casual FT** | | | | | | | | | | | | | | | | | | | | | | | | | | | | **Temporary/Casual PT** | | | | | | | | | |  | | |
|  |  | | | | | | | | | | | | | 35.6 | | | | | | | | | | | | | | | | | | | 26.8 | | | | | | | | | | | | | | | | | | | | | | | | | | | | 8.4 | | | | | | | | | | ^67^ | | |
|  |  | | | | | | | | | | | | |  | | | | | | | | | | | | | | | | | | | **CNA** | | | | | | | | | | | | | | | | | | | | | | | | | | | | **HHA** | | | | | | | | | |  | | |
|  |  | | | | | | | | | | | | |  | | | | | | | | | | | | | | | | | | | 26.0 | | | | | | | | | | | | | | | | | | | | | | | | | | | | 16.9 | | | | | | | | | | ^35^ | | |
| **Children**  % Caring for children or dependent other (SD or SE) | **DCWs Overall** | | | | | | | | | | | | | 85.4 (stayers)  90.2 (leavers) | | | | | | | | | | | | | | | | | | |  | | | | | | | | | | | | | | | | | | | | | | | | | | | |  | | | | | | | | | | ^32^ | | |
|  | **By Setting** | | | | | | | | | | | | |  | | | | | | | | | | | | | | | | | | | **NH** | | | | | | | | | | | | | | | | | | | | | | | | | | | |  | | | | | | | | | |  | | |
|  |  | | | | | | | | | | | | |  | | | | | | | | | | | | | | | | | | | 40(0.02) | | | | | | | | | | | | | | | | | | | | | | | | | | | |  | | | | | | | | | | ^56^ | | |
|  |  | | | | | | | | | | | | |  | | | | | | | | | | | | | | | | | | | 39(0.02) (tenure>1yr) | | | | | | | | | | | | | | | | | | | | | | | | | | | |  | | | | | | | | | |  | | |
|  |  | | | | | | | | | | | | |  | | | | | | | | | | | | | | | | | | | 39.3 | | | | | | | | | | | | | | | | | | | | | | | | | | | |  | | | | | | | | | | ^33^ | | |
|  | **By Immigration Status (NH)** | | | | | | | | | | | | | | | | | | | | | | | | | | | | | | | | **Immigrant** | | | | | | | | | | | | | | | | | | | | | | | | | | | | **Non-Immigrant** | | | | | | | | | |  | | |
|  |  | | | | | | | | | | | | |  | | | | | | | | | | | | | | | | | | | 40.6 | | | | | | | | | | | | | | | | | | | | | | | | | | | | 38.9 | | | | | | | | | | ^33^ | | |
|  | **By Gender** | | | | | | | | | | | | |  | | | | | | | | | | | | | | | | | | | **Female** | | | | | | | | | | | | | | | | | | | | | | | | | | | | **Male** | | | | | | | | | | ^52^ | | |
|  |  | | | | | | | | | | | | |  | | | | | | | | | | | | | | | | | | | 60.5 | | | | | | | | | | | | | | | | | | | | | | | | | | | | 52.3 | | | | | | | | | |  | | |
| % With Children under 18 | **DCWs Overall** | | | | | | | | | | | | |  | | | | | | | | | | | | | | | | | | |  | | | | | | | | | | | | | | | | | | | | | | | | | | | |  | | | | | | | | | |  | | |
|  | No children | | | | | | | | | | | | | 48 | | | | | | | | | | | | | | | | | | |  | | | | | | | | | | | | | | | | | | | | | | | | | | | |  | | | | | | | | | | ^76^ | | |
|  | Under 18 | | | | | | | | | | | | | 52 | | | | | | | | | | | | | | | | | | |  | | | | | | | | | | | | | | | | | | | | | | | | | | | |  | | | | | | | | | |  | | |
|  |  | | | | | | | | | | | | | 43 | | | | | | | | | | | | | | | | | | |  | | | | | | | | | | | | | | | | | | | | | | | | | | | |  | | | | | | | | | | ^22^ | | |
|  | **By Setting** | | | | | | | | | | | | | **H** | | | | | | | | | | | | | | | | | | | **NH** | | | | | | | | | | | | | | | | | | | | | | | | | | | | **HH** | | | | | | | | | |  | | |
|  | No children | | | | | | | | | | | | | 48.1 | | | | | | | | | | | | | | | | | | | 49.3 | | | | | | | | | | | | | | | | | | | | | | | | | | | | 66.5 | | | | | | | | | | ^81^ | | |
|  | Under 18 | | | | | | | | | | | | | 43 | | | | | | | | | | | | | | | | | | | 41 | | | | | | | | | | | | | | | | | | | | | | | | | | | | 28.8 | | | | | | | | | |  | | |
|  |  | | | | | | | | | | | | | 32 | | | | | | | | | | | | | | | | | | | 50 | | | | | | | | | | | | | | | | | | | | | | | | | | | | 40 | | | | | | | | | | ^22^ | | |
| % and # of children needing care  (SD or SE) | **By Setting** | | | | | | | | | | | | |  | | | | | | | | | | | | | | | | | | | **NH** | | | | | | | | | | | | | | | | | | | | | | | | | | | |  | | | | | | | | | |  | | |
|  | 0 | | | | | | | | | | | | |  | | | | | | | | | | | | | | | | | | | 27.62(1.16) | | | | | | | | | | | | | | | | | | | | | | | | | | | |  | | | | | | | | | | ^24^ | | |
|  | 1 | | | | | | | | | | | | |  | | | | | | | | | | | | | | | | | | | 15.49(0.91) | | | | | | | | | | | | | | | | | | | | | | | | | | | |  | | | | | | | | | |  | | |
|  | ≥2 | | | | | | | | | | | | |  | | | | | | | | | | | | | | | | | | | 12.32(0.83) | | | | | | | | | | | | | | | | | | | | | | | | | | | |  | | | | | | | | | |  | | |
|  | No children | | | | | | | | | | | | |  | | | | | | | | | | | | | | | | | | | 41.08 (1.37) | | | | | | | | | | | | | | | | | | | | | | | | | | | |  | | | | | | | | | |  | | |
| Single Mothers | **DCWs Overall** | | | | | | | | | | | | | 24 | | | | | | | | | | | | | | | | | | |  | | | | | | | | | | | | | | | | | | | | | | | | | | | |  | | | | | | | | | | ^22^ | | |
|  | **By Setting** | | | | | | | | | | | | | **H** | | | | | | | | | | | | | | | | | | | **NH** | | | | | | | | | | | | | | | | | | | | | | | | | | | | **HH** | | | | | | | | | |  | | |
|  |  | | | | | | | | | | | | | 17 | | | | | | | | | | | | | | | | | | | 28 | | | | | | | | | | | | | | | | | | | | | | | | | | | | 22 | | | | | | | | | |  | | |
| **Language** | **DCWs Overall** | | | | | | | | | | | | |  | | | | | | | | | | | | | | | | | | |  | | | | | | | | | | | | | | | | | | | | | | | | | | | |  | | | | | | | | | |  | | |
| % with English as primary language  (SD or SE) | English | | | | | | | | | | | | | 76 | | | | | | | | | | | | | | | | | | |  | | | | | | | | | | | | | | | | | | | | | | | | | | | |  | | | | | | | | | | ^78^ | | |
|  | **By Setting** | | | | | | | | | | | | | **H** | | | | | | | | | | | | | | | | | | | **NH** | | | | | | | | | | | | | | | | | | | | | | | | | | | | **HH** | | | | | | | | | |  | | |
|  | English | | | | | | | | | | | | | 80.5 | | | | | | | | | | | | | | | | | | | 84.6 | | | | | | | | | | | | | | | | | | | | | | | | | | | | 74.5 | | | | | | | | | | ^17^ | | |
|  |  | | | | | | | | | | | | |  | | | | | | | | | | | | | | | | | | | 89.0 | | | | | | | | | | | | | | | | | | | | | | | | | | | |  | | | | | | | | | | ^53^ | | |
|  |  | | | | | | | | | | | | |  | | | | | | | | | | | | | | | | | | | 88.9 | | | | | | | | | | | | | | | | | | | | | | | | | | | |  | | | | | | | | | | ^54^ | | |
|  | non-English | | | | | | | | | | | | | 19.5 | | | | | | | | | | | | | | | | | | | 15.4 | | | | | | | | | | | | | | | | | | | | | | | | | | | | 25.5 | | | | | | | | | | ^17^ | | |
|  |  | | | | | | | | | | | | |  | | | | | | | | | | | | | | | | | | | 11 (0.01) | | | | | | | | | | | | | | | | | | | | | | | | | | | |  | | | | | | | | | | ^56^ | | |
|  | **By Location** | | | | | | | | | | | | | **Urban** | | | | | | | | | | | | | | | | | | | **Micropolitan** | | | | | | | | | | | | | | | | | | | | | | | | | | | | **Other Rural** | | | | | | | | | |  | | |
|  | English | | | | | | | | | | | | | 86.1 | | | | | | | | | | | | | | | | | | | 97.3 | | | | | | | | | | | | | | | | | | | | | | | | | | | | 99.1 | | | | | | | | | | ^53^ | | |
|  | non-English | | | | | | | | | | | | | 11.9 | | | | | | | | | | | | | | | | | | | 2.7 | | | | | | | | | | | | | | | | | | | | | | | | | | | | 0.9 | | | | | | | | | |  | | |
|  | **By Immigration Status (NH)** | | | | | | | | | | | | | | | | | | | | | | | | | | | | | | | | **Immigrant** | | | | | | | | | | | | | | | | | | | | | | | | | | | | **Non-Immigrant** | | | | | | | | | |  | | |
|  | non-English | | | | | | | | | | | | |  | | | | | | | | | | | | | | | | | | | 50.9 | | | | | | | | | | | | | | | | | | | | | | | | | | | | 1.0 | | | | | | | | | | ^33^ | | |
| % language use on job | **Overall (NH)** | | | | | | | | | | | | |  | | | | | | | | | | | | | | | | | | |  | | | | | | | | | | | | | | | | | | | | | | | | | | | |  | | | | | | | | | |  | | |
|  | Has difficulty communicating with residents because of language | | | | | | | | | | | | | | | | | | | | | | | | | | | | | | | | 43.0 | | | | | | | | | | | | | | | | | | | | | | | | | | | |  | | | | | | | | | | ^33^ | | |
|  | Has difficulty communicating with residents because of language | | | | | | | | | | | | | | | | | | | | | | | | | | | | | | | | 26.0 | | | | | | | | | | | | | | | | | | | | | | | | | | | |  | | | | | | | | | |  | | |
|  | **By Setting** | | | | | | | | | | | | | | | | | | | | | | | | | | | | | | | | **Community Care** | | | | | | | | | | | | | | | | | | | | | | | | | | | | **Residential Care** | | | | | | | | | |  | | |
|  | Staff speak language other than English | | | | | | | | | | | | | | | | | | | | | | | | | | | | | | | | 23.9 | | | | | | | | | | | | | | | | | | | | | | | | | | | | 29.4 | | | | | | | | | | ^15^ | | |
|  | Bilingual direct care staff that use their second language in their job | | | | | | | | | | | | | | | | | | | | | | | | | | | | | | | | 65.6 | | | | | | | | | | | | | | | | | | | | | | | | | | | | 46.7 | | | | | | | | | |  | | |
|  | **By Immigration Status (NH)** | | | | | | | | | | | | | | | | | | | | | | | | | | | | | | | | **Immigrant** | | | | | | | | | | | | | | | | | | | | | | | | | | | | **Non-Immigrant** | | | | | | | | | |  | | |
|  | Has difficulty communicating with residents because of language | | | | | | | | | | | | | | | | | | | | | | | | | | | | | | | | 51.3 | | | | | | | | | | | | | | | | | | | | | | | | | | | | 40.9 | | | | | | | | | | ^33^ | | |
|  | Has difficulty communicating with residents because of language | | | | | | | | | | | | | | | | | | | | | | | | | | | | | | | | 31.3 | | | | | | | | | | | | | | | | | | | | | | | | | | | | 24.7 | | | | | | | | | |  | | |
| **Immigration Status** | **DCWs Overall** | | | | | | | | | | | | |  | | | | | | | | | | | | | | | | | | |  | | | | | | | | | | | | | | | | | | | | | | | | | | | |  | | | | | | | | | |  | | |
|  |  | | | | | | | | | | | | |  | | | | | | | | | | | | | | | | | | |  | | | | | | | | | | | | | | | | | | | | | | | | | | | |  | | | | | | | | | |  | | |
| United States | US citizen | | | | | | | | | | | | | 88 | | | | | | | | | | | | | | | | | | |  | | | | | | | | | | | | | | | | | | | | | | | | | | | |  | | | | | | | | | | ^76^ | | |
| % US citizen in Direct care occupations  (SD or SE) | Foreign born | | | | | | | | | | | | | 20 | | | | | | | | | | | | | | | | | | |  | | | | | | | | | | | | | | | | | | | | | | | | | | | |  | | | | | | | | | | ^22^ | | |
|  |  | | | | | | | | | | | | | 85.6 (Of Foreign workers in the US, 85.6% are in Direct Care) | | | | | | | | | | | | | | | | | | | | | | | | | | | | | | | | | | | | | | | | | | | | | | | | | | | | | | | | | ^16^ | | |
|  | **By Setting** | | | | | | | | | | | | | **H** | | | | | | | | | | | | | | | | | | | **NH** | | | | | | | | | | | | | | | | | | | | | | | | | | | | **HH** | | | | | | | | | |  | | |
|  | US citizen | | | | | | | | | | | | |  | | | | | | | | | | | | | | | | | | | 92 | | | | | | | | | | | | | | | | | | | | | | | | | | | |  | | | | | | | | | | ^55^ | | |
|  |  | | | | | | | | | | | | |  | | | | | | | | | | | | | | | | | | | 90.9 | | | | | | | | | | | | | | | | | | | | | | | | | | | |  | | | | | | | | | | ^53^ | | |
|  | US born | | | | | | | | | | | | | 81.5 | | | | | | | | | | | | | | | | | | | 85.5 | | | | | | | | | | | | | | | | | | | | | | | | | | | | 75.1 | | | | | | | | | | ^17^ | | |
|  |  | | | | | | | | | | | | | 85.3 | | | | | | | | | | | | | | | | | | | 85.4 | | | | | | | | | | | | | | | | | | | | | | | | | | | | 74.5 | | | | | | | | | | ^39^ | | |
|  | Foreign born and  naturalized | | | | | | | | | | | | | 9.4 | | | | | | | | | | | | | | | | | | | 6.1 | | | | | | | | | | | | | | | | | | | | | | | | | | | | 10.8 | | | | | | | | | | ^17^ | | |
|  |  | | | | | | | | | | | | | 6 | | | | | | | | | | | | | | | | | | | 4 | | | | | | | | | | | | | | | | | | | | | | | | | | | | 5.6 | | | | | | | | | | ^39^ | | |
|  | Not US citizen | | | | | | | | | | | | | 7.7 | | | | | | | | | | | | | | | | | | | 7.2 | | | | | | | | | | | | | | | | | | | | | | | | | | | | 12.5 | | | | | | | | | | ^17^ | | |
|  |  | | | | | | | | | | | | | 6.4 | | | | | | | | | | | | | | | | | | | 8.2 | | | | | | | | | | | | | | | | | | | | | | | | | | | | 16.2 | | | | | | | | | | ^39^ | | |
|  | Foreign born | | | | | | | | | | | | | 19 | | | | | | | | | | | | | | | | | | | 17 | | | | | | | | | | | | | | | | | | | | | | | | | | | | 22 | | | | | | | | | | ^22^ | | |
|  |  | | | | | | | | | | | | | 17.9 | | | | | | | | | | | | | | | | | | | 16.9 | | | | | | | | | | | | | | | | | | | | | | | | | | | | 27.3 | | | | | | | | | | ^16^ | | |
|  |  | | | | | | | | | | | | |  | | | | | | | | | | | | | | | | | | | 19 (.02) | | | | | | | | | | | | | | | | | | | | | | | | | | | |  | | | | | | | | | | ^56^ | | |
|  |  | | | | | | | | | | | | |  | | | | | | | | | | | | | | | | | | | 21(.02) (tenure >1yr) | | | | | | | | | | | | | | | | | | | | | | | | | | | |  | | | | | | | | | |  | | |
|  | **By Location (NH)** | | | | | | | | | | | | | **Urban** | | | | | | | | | | | | | | | | | | | **Micropolitan** | | | | | | | | | | | | | | | | | | | | | | | | | | | | **Other Rural** | | | | | | | | | |  | | |
|  | US citizen | | | | | | | | | | | | | 88.4** | | | | | | | | | | | | | | | | | | | 98.3** | | | | | | | | | | | | | | | | | | | | | | | | | | | | 99.3** | | | | | | | | | | ^53^ | | |
|  | **By Immigration status (NH)** | | | | | | | | | | | | | | | | | | | | | | | | | | | | | | | | **Immigrant** | | | | | | | | | | | | | | | | | | | | | | | | | | | | **Non-immigrant** | | | | | | | | | |  | | |
|  | Minority status | | | | | | | | | | | | | | | | | | | | | | | | | | | | | | | | 78.7 | | | | | | | | | | | | | | | | | | | | | | | | | | | | 38.6 | | | | | | | | | | ^33^ | | |
|  | Non-American citizenship | | | | | | | | | | | | | | | | | | | | | | | | | | | | | | | | 45.1 | | | | | | | | | | | | | | | | | | | | | | | | | | | |  | | | | | | | | | |  | | |
|  | **By Gender** | | | | | | | | | | | | |  | | | | | | | | | | | | | | | | | | | **Females** | | | | | | | | | | | | | | | | | | | | | | | | | | | | **Males** | | | | | | | | | |  | | |
|  | Non-US citizen | | | | | | | | | | | | |  | | | | | | | | | | | | | | | | | | | 6 | | | | | | | | | | | | | | | | | | | | | | | | | | | | 17.3 | | | | | | | | | | ^52^ | | |
| % by place of birth | **Place of birth** of foreign DCWs (share in each occupational group by nationality, top 3 countries) | | | | | | | | | | | | | **Mexico and Central America** | | | | | | | | | | | | | | | | | | | **Caribbean and Atlantic Islands** | | | | | | | | | | | | | | | | | | | | | | | | | | | | **South America** | | | | | | | | | |  | | |
|  |  |  |  |  |  |  |  |  |  |  |  |  |  | 95 | | | | | | | | | | | | | | | | | | | 88.7 | | | | | | | | | | | | | | | | | | | | | | | | | | | | 88.3 | | | | | | | | | | ^16^ | | |
| Australia | **By Setting** | | | | | | | | | | | | |  | | | | | | | | | | | | | | | | | | | **Community care** | | | | | | | | | | | | | | | | | | | | | | | | | | | | **Residential care** | | | | | | | | | |  | | |
|  | Australia | | | | | | | | | | | | |  | | | | | | | | | | | | | | | | | | | 73.3 | | | | | | | | | | | | | | | | | | | | | | | | | | | | 67.5 | | | | | | | | | | ^15^ | | |
|  | All Overseas countries | | | | | | | | | | | | | | | | | | | | | | | | | | | | | | | | 26.7 | | | | | | | | | | | | | | | | | | | | | | | | | | | | 32.5 | | | | | | | | | |  | | |
|  | English-speaking countries | | | | | | | | | | | | | | | | | | | | | | | | | | | | | | | | 11.9 | | | | | | | | | | | | | | | | | | | | | | | | | | | | 12.7 | | | | | | | | | |  | | |
|  | UK, Ireland, South Africa | | | | | | | | | | | | | | | | | | | | | | | | | | | | | | | | 8.5 | | | | | | | | | | | | | | | | | | | | | | | | | | | | 9.2 | | | | | | | | | |  | | |
|  | New Zealand | | | | | | | | | | | | | | | | | | | | | | | | | | | | | | | | 3.4 | | | | | | | | | | | | | | | | | | | | | | | | | | | | 3.5 | | | | | | | | | |  | | |
|  | Non-English-speaking countries | | | | | | | | | | | | | | | | | | | | | | | | | | | | | | | | 14.9 | | | | | | | | | | | | | | | | | | | | | | | | | | | | 19.9 | | | | | | | | | |  | | |
|  | China, Vietnam, Philippines | | | | | | | | | | | | | | | | | | | | | | | | | | | | | | | | 2.3 | | | | | | | | | | | | | | | | | | | | | | | | | | | | 5.2 | | | | | | | | | |  | | |
|  | Italy, Greece, Germany, Netherlands | | | | | | | | | | | | | | | | | | | | | | | | | | | | | | | | 3.1 | | | | | | | | | | | | | | | | | | | | | | | | | | | | 1.9 | | | | | | | | | |  | | |
|  | India | | | | | | | | | | | | | | | | | | | | | | | | | | | | | | | | 0.4 | | | | | | | | | | | | | | | | | | | | | | | | | | | | 1.3 | | | | | | | | | |  | | |
|  | Poland | | | | | | | | | | | | | | | | | | | | | | | | | | | | | | | | 1.1 | | | | | | | | | | | | | | | | | | | | | | | | | | | | 0.3 | | | | | | | | | |  | | |
|  | Fiji | | | | | | | | | | | | | | | | | | | | | | | | | | | | | | | | 0.3 | | | | | | | | | | | | | | | | | | | | | | | | | | | | 1.6 | | | | | | | | | |  | | |
|  | Other | | | | | | | | | | | | | | | | | | | | | | | | | | | | | | | | 7.7 | | | | | | | | | | | | | | | | | | | | | | | | | | | | 9.6 | | | | | | | | | |  | | |
| Canada | **By Setting** | | | | | | | | | | | | | | | | | | | | | | | | | | | | | | | |  | | | | | | | | | | | | | | | | | | | | | | | | | | | | **HH** | | | | | | | | | |  | | |
| % by place of birth | Canada | | | | | | | | | | | | | | | | | | | | | | | | | | | | | | | |  | | | | | | | | | | | | | | | | | | | | | | | | | | | | 52.63 | | | | | | | | | | ^41^ | | |
|  | Asia | | | | | | | | | | | | | | | | | | | | | | | | | | | | | | | |  | | | | | | | | | | | | | | | | | | | | | | | | | | | | 36.84 | | | | | | | | | |  | | |
|  | Other | | | | | | | | | | | | | | | | | | | | | | | | | | | | | | | |  | | | | | | | | | | | | | | | | | | | | | | | | | | | | 10.53 | | | | | | | | | |  | | |
| **Ethnicity**  % by Ethnicity  (SD or SE) | **DCWs Overall** | | | | | | | | | | | | |  | | | | | | | | | | | | | | | | | | |  | | | | | | | | | | | | | | | | | | | | | | | | | | | |  | | | | | | | | | |  | | |
|  | Caucasian | | | | | | | | | | | | | 55.4 | | | | | | | | | | | | | | | | | | |  | | | | | | | | | | | | | | | | | | | | | | | | | | | |  | | | | | | | | | | ^77^ | | |
|  |  | | | | | | | | | | | | | 47 | | | | | | | | | | | | | | | | | | |  | | | | | | | | | | | | | | | | | | | | | | | | | | | |  | | | | | | | | | | ^76^ | | |
|  |  | | | | | | | | | | | | | 53.32 | | | | | | | | | | | | | | | | | | |  | | | | | | | | | | | | | | | | | | | | | | | | | | | |  | | | | | | | | | | ^51^ | | |
|  |  | | | | | | | | | | | | | 45.16 | | | | | | | | | | | | | | | | | | |  | | | | | | | | | | | | | | | | | | | | | | | | | | | |  | | | | | | | | | | ^49^ | | |
|  |  | | | | | | | | | | | | | 51 | | | | | | | | | | | | | | | | | | |  | | | | | | | | | | | | | | | | | | | | | | | | | | | |  | | | | | | | | | | ^22^ | | |
|  |  | | | | | | | | | | | | | ~66 | | | | | | | | | | | | | | | | | | |  | | | | | | | | | | | | | | | | | | | | | | | | | | | |  | | | | | | | | | | ^79^ | | |
|  |  | | | | | | | | | | | | | 60 | | | | | | | | | | | | | | | | | | |  | | | | | | | | | | | | | | | | | | | | | | | | | | | |  | | | | | | | | | | ^14^ | | |
|  | Non-Caucasian | | | | | | | | | | | | | 58.4 | | | | | | | | | | | | | | | | | | |  | | | | | | | | | | | | | | | | | | | | | | | | | | | |  | | | | | | | | | | ^49^ | | |
|  |  | | | | | | | | | | | | | 59.4 (found that race is significant predictor of full time employment) | | | | | | | | | | | | | | | | | | | | | | | | | | | | | | | | | | | | | | | | | | | | | | | | | | | | | | | | | ^34^ | | |
|  | African American | | | | | | | | | | | | | 29.0 | | | | | | | | | | | | | | | | | | |  | | | | | | | | | | | | | | | | | | | | | | | | | | | |  | | | | | | | | | | ^77^ | | |
|  |  | | | | | | | | | | | | | 34.3 (47.5) | | | | | | | | | | | | | | | | | | |  | | | | | | | | | | | | | | | | | | | | | | | | | | | |  | | | | | | | | | | ^72^ | | |
|  |  | | | | | | | | | | | | | 35 | | | | | | | | | | | | | | | | | | |  | | | | | | | | | | | | | | | | | | | | | | | | | | | |  | | | | | | | | | | ^76^ | | |
|  |  | | | | | | | | | | | | | 38.74 | | | | | | | | | | | | | | | | | | |  | | | | | | | | | | | | | | | | | | | | | | | | | | | |  | | | | | | | | | | ^51^ | | |
|  |  | | | | | | | | | | | | | 29 | | | | | | | | | | | | | | | | | | |  | | | | | | | | | | | | | | | | | | | | | | | | | | | |  | | | | | | | | | | ^22^ | | |
|  |  | | | | | | | | | | | | | 27 | | | | | | | | | | | | | | | | | | |  | | | | | | | | | | | | | | | | | | | | | | | | | | | |  | | | | | | | | | | ^14^ | | |
|  | Hispanic | | | | | | | | | | | | | 10.5 | | | | | | | | | | | | | | | | | | |  | | | | | | | | | | | | | | | | | | | | | | | | | | | |  | | | | | | | | | | ^77^ | | |
|  |  | | | | | | | | | | | | | 11.8 (32.2) | | | | | | | | | | | | | | | | | | |  | | | | | | | | | | | | | | | | | | | | | | | | | | | |  | | | | | | | | | | ^72^ | | |
|  |  | | | | | | | | | | | | | 11 | | | | | | | | | | | | | | | | | | |  | | | | | | | | | | | | | | | | | | | | | | | | | | | |  | | | | | | | | | | ^76^ | | |
|  |  | | | | | | | | | | | | | 15 | | | | | | | | | | | | | | | | | | |  | | | | | | | | | | | | | | | | | | | | | | | | | | | |  | | | | | | | | | | ^22^ | | |
|  | Other | | | | | | | | | | | | | 5.1 | | | | | | | | | | | | | | | | | | |  | | | | | | | | | | | | | | | | | | | | | | | | | | | |  | | | | | | | | | | ^77^ | | |
|  |  | | | | | | | | | | | | | 4.2 (20.0) | | | | | | | | | | | | | | | | | | |  | | | | | | | | | | | | | | | | | | | | | | | | | | | |  | | | | | | | | | | ^72^ | | |
|  |  | | | | | | | | | | | | | 7 | | | | | | | | | | | | | | | | | | |  | | | | | | | | | | | | | | | | | | | | | | | | | | | |  | | | | | | | | | | ^76^ | | |
|  |  | | | | | | | | | | | | | 5 | | | | | | | | | | | | | | | | | | |  | | | | | | | | | | | | | | | | | | | | | | | | | | | |  | | | | | | | | | | ^22^ | | |
|  | **By Setting** | | | | | | | | | | | | | **H** | | | | | | | | | | | | | | | | | | | **NH** | | | | | | | | | | | | | | | | | | | | | | | | | | | | **HH** | | | | | | | | | |  | | |
|  | Caucasian | | | | | | | | | | | | | 66.7 | | | | | | | | | | | | | | | | | | | 69.1 | | | | | | | | | | | | | | | | | | | | | | | | | | | | 69.7 | | | | | | | | | | ^81^ | | |
|  |  | | | | | | | | | | | | | 69 | | | | | | | | | | | | | | | | | | | 70.6 | | | | | | | | | | | | | | | | | | | | | | | | | | | | 70.4 | | | | | | | | | | ^39^ | | |
|  |  | | | | | | | | | | | | | 48.4 | | | | | | | | | | | | | | | | | | | 55.6 | | | | | | | | | | | | | | | | | | | | | | | | | | | | 50.3 | | | | | | | | | | ^17^ | | |
|  |  | | | | | | | | | | | | | 55 | | | | | | | | | | | | | | | | | | | 51 | | | | | | | | | | | | | | | | | | | | | | | | | | | | 49 | | | | | | | | | | ^22^ | | |
|  |  | | | | | | | | | | | | |  | | | | | | | | | | | | | | | | | | | 46.8 | | | | | | | | | | | | | | | | | | | | | | | | | | | |  | | | | | | | | | | ^53^ | | |
|  |  | | | | | | | | | | | | |  | | | | | | | | | | | | | | | | | | | 53.44 | | | | | | | | | | | | | | | | | | | | | | | | | | | |  | | | | | | | | | | ^24^ | | |
|  |  | | | | | | | | | | | | |  | | | | | | | | | | | | | | | | | | | 53.3 (1.9) | | | | | | | | | | | | | | | | | | | | | | | | | | | |  | | | | | | | | | | ^21^ | | |
|  | Non-Caucasian | | | | | | | | | | | | |  | | | | | | | | | | | | | | | | | | |  | | | | | | | | | | | | | | | | | | | | | | | | | | | | 14.5 | | | | | | | | | | ^73^ | | |
|  |  | | | | | | | | | | | | |  | | | | | | | | | | | | | | | | | | | 31 | | | | | | | | | | | | | | | | | | | | | | | | | | | | 32 | | | | | | | | | | ^79^ | | |
|  |  | | | | | | | | | | | | |  | | | | | | | | | | | | | | | | | | | 38 | | | | | | | | | | | | | | | | | | | | | | | | | | | |  | | | | | | | | | | ^50^ | | |
|  |  | | | | | | | | | | | | |  | | | | | | | | | | | | | | | | | | | 46 | | | | | | | | | | | | | | | | | | | | | | | | | | | |  | | | | | | | | | | ^55^ | | |
|  |  | | | | | | | | | | | | |  | | | | | | | | | | | | | | | | | | | 74 | | | | | | | | | | | | | | | | | | | | | | | | | | | |  | | | | | | | | | | ^80^ | | |
|  | African American | | | | | | | | | | | | | 31.8 | | | | | | | | | | | | | | | | | | | 26.9 | | | | | | | | | | | | | | | | | | | | | | | | | | | | 29.2 | | | | | | | | | | ^81^ | | |
|  |  | | | | | | | | | | | | | 26.1 | | | | | | | | | | | | | | | | | | | 25 | | | | | | | | | | | | | | | | | | | | | | | | | | | | 25.9 | | | | | | | | | | ^39^ | | |
|  |  | | | | | | | | | | | | | 33 | | | | | | | | | | | | | | | | | | | 30.4 | | | | | | | | | | | | | | | | | | | | | | | | | | | | 26.4 | | | | | | | | | | ^17^ | | |
|  |  | | | | | | | | | | | | | 30 | | | | | | | | | | | | | | | | | | | 35 | | | | | | | | | | | | | | | | | | | | | | | | | | | | 24 | | | | | | | | | | ^22^ | | |
|  |  | | | | | | | | | | | | |  | | | | | | | | | | | | | | | | | | | 37.0 | | | | | | | | | | | | | | | | | | | | | | | | | | | |  | | | | | | | | | | ^53^ | | |
|  |  | | | | | | | | | | | | |  | | | | | | | | | | | | | | | | | | | 38.66 | | | | | | | | | | | | | | | | | | | | | | | | | | | |  | | | | | | | | | | ^24^ | | |
|  |  | | | | | | | | | | | | |  | | | | | | | | | | | | | | | | | | | 38.8 (1.9) | | | | | | | | | | | | | | | | | | | | | | | | | | | |  | | | | | | | | | | ^21^ | | |
|  |  | | | | | | | | | | | | |  | | | | | | | | | | | | | | | | | | |  | | | | | | | | | | | | | | | | | | | | | | | | | | | | 69.5 | | | | | | | | | | ^73^ | | |
|  | Hispanic | | | | | | | | | | | | | 11 | | | | | | | | | | | | | | | | | | | 10 | | | | | | | | | | | | | | | | | | | | | | | | | | | | 21 | | | | | | | | | | ^22^ | | |
|  |  | | | | | | | | | | | | | 10.7 | | | | | | | | | | | | | | | | | | | 7.8 | | | | | | | | | | | | | | | | | | | | | | | | | | | | 15.9 | | | | | | | | | | ^17^ | | |
|  |  | | | | | | | | | | | | |  | | | | | | | | | | | | | | | | | | | 9.5 | | | | | | | | | | | | | | | | | | | | | | | | | | | |  | | | | | | | | | | ^53^ | | |
|  |  | | | | | | | | | | | | |  | | | | | | | | | | | | | | | | | | | 9.5 (0.9) | | | | | | | | | | | | | | | | | | | | | | | | | | | |  | | | | | | | | | | ^21^ | | |
|  |  | | | | | | | | | | | | |  | | | | | | | | | | | | | | | | | | | 10 | | | | | | | | | | | | | | | | | | | | | | | | | | | |  | | | | | | | | | | ^55^ | | |
|  |  | | | | | | | | | | | | |  | | | | | | | | | | | | | | | | | | | 10 | | | | | | | | | | | | | | | | | | | | | | | | | | | |  | | | | | | | | | | ^50^ | | |
|  |  | | | | | | | | | | | | |  | | | | | | | | | | | | | | | | | | |  | | | | | | | | | | | | | | | | | | | | | | | | | | | | 3.8 | | | | | | | | | | ^73^ | | |
|  | Other | | | | | | | | | | | | | 4.9 | | | | | | | | | | | | | | | | | | | 4.4 | | | | | | | | | | | | | | | | | | | | | | | | | | | | 3.7 | | | | | | | | | | ^39^ | | |
|  |  | | | | | | | | | | | | | 5 | | | | | | | | | | | | | | | | | | | 4 | | | | | | | | | | | | | | | | | | | | | | | | | | | | 7 | | | | | | | | | | ^22^ | | |
|  |  | | | | | | | | | | | | |  | | | | | | | | | | | | | | | | | | | 6.7 | | | | | | | | | | | | | | | | | | | | | | | | | | | |  | | | | | | | | | | ^53^ | | |
|  |  | | | | | | | | | | | | |  | | | | | | | | | | | | | | | | | | | 7.9 (0.9) | | | | | | | | | | | | | | | | | | | | | | | | | | | |  | | | | | | | | | | ^21^ | | |
|  |  | | | | | | | | | | | | |  | | | | | | | | | | | | | | | | | | | **Assisted Living** | | | | | | | | | | | | | | | | | | | | | | | | | | | | **Adult Day Services** | | | | | | | | | |  | | |
|  | Non-Caucasian | | | | | | | | | | | | |  | | | | | | | | | | | | | | | | | | | 50 | | | | | | | | | | | | | | | | | | | | | | | | | | | | 24 | | | | | | | | | | ^79^ | | |
|  | **By Location (NH)** | | | | | | | | | | | | | **Urban** | | | | | | | | | | | | | | | | | | | **Micropolitan** | | | | | | | | | | | | | | | | | | | | | | | | | | | | **Other Rural** | | | | | | | | | |  | | |
|  | Caucasian | | | | | | | | | | | | | 39.8 | | | | | | | | | | | | | | | | | | | 64.1 | | | | | | | | | | | | | | | | | | | | | | | | | | | | 74.8 | | | | | | | | | | ^53^ | | |
|  | African American | | | | | | | | | | | | | 42.1 | | | | | | | | | | | | | | | | | | | 23.8 | | | | | | | | | | | | | | | | | | | | | | | | | | | | 17.5 | | | | | | | | | |  | | |
|  | Hispanic | | | | | | | | | | | | | 10.2 | | | | | | | | | | | | | | | | | | | 9.0 | | | | | | | | | | | | | | | | | | | | | | | | | | | | 5.2 | | | | | | | | | |  | | |
|  | Other | | | | | | | | | | | | | 7.9 | | | | | | | | | | | | | | | | | | | 3.1 | | | | | | | | | | | | | | | | | | | | | | | | | | | | 2.5 | | | | | | | | | |  | | |
|  | **By Gender** | | | | | | | | | | | | |  | | | | | | | | | | | | | | | | | | | **Female** | | | | | | | | | | | | | | | | | | | | | | | | | | | | **Male** | | | | | | | | | |  | | |
|  | Caucasian | | | | | | | | | | | | |  | | | | | | | | | | | | | | | | | | | 55.1 | | | | | | | | | | | | | | | | | | | | | | | | | | | | 45.9 | | | | | | | | | | ^52^ | | |
|  | African American | | | | | | | | | | | | |  | | | | | | | | | | | | | | | | | | | 29.7 | | | | | | | | | | | | | | | | | | | | | | | | | | | | 32.2 | | | | | | | | | |  | | |
|  | Other | | | | | | | | | | | | |  | | | | | | | | | | | | | | | | | | | 15.2 | | | | | | | | | | | | | | | | | | | | | | | | | | | | 21.9 | | | | | | | | | |  | | |
|  | **By Job Status** | | | | | | | | | | | | |  | | | | | | | | | | | | | | | | | | | **CNA** | | | | | | | | | | | | | | | | | | | | | | | | | | | | **HHA** | | | | | | | | | |  | | |
|  | Caucasian | | | | | | | | | | | | |  | | | | | | | | | | | | | | | | | | | 47.6 | | | | | | | | | | | | | | | | | | | | | | | | | | | | 50.8 | | | | | | | | | | ^35^ | | |
|  | African American | | | | | | | | | | | | |  | | | | | | | | | | | | | | | | | | | 36.4 | | | | | | | | | | | | | | | | | | | | | | | | | | | | 34.1 | | | | | | | | | |  | | |
|  | Hispanic | | | | | | | | | | | | |  | | | | | | | | | | | | | | | | | | | 9.4 | | | | | | | | | | | | | | | | | | | | | | | | | | | | 8.0 | | | | | | | | | |  | | |
|  | Other | | | | | | | | | | | | |  | | | | | | | | | | | | | | | | | | | 6.6 | | | | | | | | | | | | | | | | | | | | | | | | | | | | 7.1 | | | | | | | | | |  | | |
| **Gender**  % of female DCWs | **DCWs Overall** | | | | | | | | | | | | | 83.1 | | | | | | | | | | | | | | | | | | |  | | | | | | | | | | | | | | | | | | | | | | | | | | | |  | | | | | | | | | | ^32^ | | |
|  |  | | | | | | | | | | | | | 85.7 (Students) | | | | | | | | | | | | | | | | | | |  | | | | | | | | | | | | | | | | | | | | | | | | | | | |  | | | | | | | | | | ^19^ | | |
|  |  | | | | | | | | | | | | | 87.2 | | | | | | | | | | | | | | | | | | |  | | | | | | | | | | | | | | | | | | | | | | | | | | | |  | | | | | | | | | | ^77^ | | |
|  |  | | | | | | | | | | | | | 89 | | | | | | | | | | | | | | | | | | |  | | | | | | | | | | | | | | | | | | | | | | | | | | | |  | | | | | | | | | | ^22^ | | |
|  |  | | | | | | | | | | | | | 91 | | | | | | | | | | | | | | | | | | |  | | | | | | | | | | | | | | | | | | | | | | | | | | | |  | | | | | | | | | | ^14^ | | |
|  |  | | | | | | | | | | | | | 92 | | | | | | | | | | | | | | | | | | |  | | | | | | | | | | | | | | | | | | | | | | | | | | | |  | | | | | | | | | | ^51^ | | |
|  |  | | | | | | | | | | | | | 92.6 (LTC) | | | | | | | | | | | | | | | | | | |  | | | | | | | | | | | | | | | | | | | | | | | | | | | |  | | | | | | | | | | ^62^ | | |
|  |  | | | | | | | | | | | | | 94.6 | | | | | | | | | | | | | | | | | | |  | | | | | | | | | | | | | | | | | | | | | | | | | | | |  | | | | | | | | | | ^34^ | | |
|  |  | | | | | | | | | | | | | 95.7 | | | | | | | | | | | | | | | | | | |  | | | | | | | | | | | | | | | | | | | | | | | | | | | |  | | | | | | | | | | ^70^ | | |
|  |  | | | | | | | | | | | | | 98.3 | | | | | | | | | | | | | | | | | | |  | | | | | | | | | | | | | | | | | | | | | | | | | | | |  | | | | | | | | | | ^66^ | | |
| % of male DCWs |  | | | | | | | | | | | | | 1.7 | | | | | | | | | | | | | | | | | | |  | | | | | | | | | | | | | | | | | | | | | | | | | | | |  | | | | | | | | | | ^66^ | | |
|  |  | | | | | | | | | | | | | 7.3 (LTC) | | | | | | | | | | | | | | | | | | |  | | | | | | | | | | | | | | | | | | | | | | | | | | | |  | | | | | | | | | | ^62^ | | |
|  |  | | | | | | | | | | | | | 7.7 | | | | | | | | | | | | | | | | | | |  | | | | | | | | | | | | | | | | | | | | | | | | | | | |  | | | | | | | | | | ^52^ | | |
| % of female DCWs  (SD or SE) | **By Setting** | | | | | | | | | | | | | **H** | | | | | | | | | | | | | | | | | | | **NH** | | | | | | | | | | | | | | | | | | | | | | | | | | | | **HH** | | | | | | | | | |  | | |
|  |  | | | | | | | | | | | | | 81.1 | | | | | | | | | | | | | | | | | | | 90.1 | | | | | | | | | | | | | | | | | | | | | | | | | | | | 88.4 | | | | | | | | | | ^39^ | | |
|  |  | | | | | | | | | | | | | 81.2 | | | | | | | | | | | | | | | | | | | 91.3 | | | | | | | | | | | | | | | | | | | | | | | | | | | | 91.8 | | | | | | | | | | ^17^ | | |
|  |  | | | | | | | | | | | | |  | | | | | | | | | | | | | | | | | | | 91.98 | | | | | | | | | | | | | | | | | | | | | | | | | | | |  | | | | | | | | | | ^24^ | | |
|  |  | | | | | | | | | | | | |  | | | | | | | | | | | | | | | | | | | 92.3 (0.7) | | | | | | | | | | | | | | | | | | | | | | | | | | | |  | | | | | | | | | | ^21^ | | |
|  |  | | | | | | | | | | | | |  | | | | | | | | | | | | | | | | | | | 92.3 | | | | | | | | | | | | | | | | | | | | | | | | | | | |  | | | | | | | | | | ^53^ | | |
|  |  | | | | | | | | | | | | |  | | | | | | | | | | | | | | | | | | | 92.3 | | | | | | | | | | | | | | | | | | | | | | | | | | | |  | | | | | | | | | | ^54^ | | |
|  |  | | | | | | | | | | | | |  | | | | | | | | | | | | | | | | | | | 92.4 (Control) | | | | | | | | | | | | | | | | | | | | | | | | | | | |  | | | | | | | | | | ^75^ | | |
|  |  | | | | | | | | | | | | |  | | | | | | | | | | | | | | | | | | | 93.4 (Treatment) | | | | | | | | | | | | | | | | | | | | | | | | | | | |  | | | | | | | | | |  | | |
|  |  | | | | | | | | | | | | |  | | | | | | | | | | | | | | | | | | | 98 | | | | | | | | | | | | | | | | | | | | | | | | | | | |  | | | | | | | | | | ^80^ | | |
|  |  | | | | | | | | | | | | |  | | | | | | | | | | | | | | | | | | |  | | | | | | | | | | | | | | | | | | | | | | | | | | | | 97.2 | | | | | | | | | | ^27^ | | |
|  |  | | | | | | | | | | | | |  | | | | | | | | | | | | | | | | | | |  | | | | | | | | | | | | | | | | | | | | | | | | | | | | 96.49 | | | | | | | | | | ^41^ | | |
| % of male DCWs  (SD or SE) | **By Setting** | | | | | | | | | | | | | **H** | | | | | | | | | | | | | | | | | | | **NH** | | | | | | | | | | | | | | | | | | | | | | | | | | | | **HH** | | | | | | | | | |  | | |
|  |  | | | | | | | | | | | | |  | | | | | | | | | | | | | | | | | | |  | | | | | | | | | | | | | | | | | | | | | | | | | | | | 7.1 | | | | | | | | | | ^73^ | | |
|  |  | | | | | | | | | | | | |  | | | | | | | | | | | | | | | | | | |  | | | | | | | | | | | | | | | | | | | | | | | | | | | | 7.7 (0.7) | | | | | | | | | | ^21^ | | |
|  |  | | | | | | | | | | | | |  | | | | | | | | | | | | | | | | | | |  | | | | | | | | | | | | | | | | | | | | | | | | | | | | 8 | | | | | | | | | | ^55^ | | |
|  |  | | | | | | | | | | | | |  | | | | | | | | | | | | | | | | | | |  | | | | | | | | | | | | | | | | | | | | | | | | | | | | 8 | | | | | | | | | | ^50^ | | |
|  |  | | | | | | | | | | | | |  | | | | | | | | | | | | | | | | | | |  | | | | | | | | | | | | | | | | | | | | | | | | | | | | 30.1 | | | | | | | | | | ^68^ | | |
|  |  | | | | | | | | | | | | |  | | | | | | | | | | | | | | | | | | | **Assisted Living** | | | | | | | | | | | | | | | | | | | | | | | | | | | | **Group home** | | | | | | | | | | ^68^ | | |
|  |  | | | | | | | | | | | | |  | | | | | | | | | | | | | | | | | | | 32.4 | | | | | | | | | | | | | | | | | | | | | | | | | | | | 29.2 | | | | | | | | | |  | | |
|  |  | | | | | | | | | | | | |  | | | | | | | | | | | | | | | | | | | 8 (.01) | | | | | | | | | | | | | | | | | | | | | | | | | | | |  | | | | | | | | | | ^56^ | | |
|  |  | | | | | | | | | | | | |  | | | | | | | | | | | | | | | | | | | 7 (.01)(tenure >1 year) | | | | | | | | | | | | | | | | | | | | | | | | | | | |  | | | | | | | | | |  | | |
|  |  | | | | | | | | | | | | |  | | | | | | | | | | | | | | | | | | | **Geriatric inter-med. facility** | | | | | | | | | | | | | | | | | | | | | | | | | | | |  | | | | | | | | | |  | | |
|  |  | | | | | | | | | | | | |  | | | | | | | | | | | | | | | | | | | 24.8 | | | | | | | | | | | | | | | | | | | | | | | | | | | |  | | | | | | | | | | ^68^ | | |
| % of female DCWs | **By Location (NH)** | | | | | | | | | | | | | **Urban** | | | | | | | | | | | | | | | | | | | **Micropolitan** | | | | | | | | | | | | | | | | | | | | | | | | | | | | **Other Rural** | | | | | | | | | |  | | |
|  |  | | | | | | | | | | | | | 91.5** | | | | | | | | | | | | | | | | | | | 95.4** | | | | | | | | | | | | | | | | | | | | | | | | | | | | 94.1** | | | | | | | | | | ^53^ | | |
| % of male DCWs | **By Immigration Status (NH)** | | | | | | | | | | | | | | | | | | | | | | | | | | | | | | | | **Immigrant** | | | | | | | | | | | | | | | | | | | | | | | | | | | | **Non-immigrant** | | | | | | | | | |  | | |
|  |  | | | | | | | | | | | | |  | | | | | | | | | | | | | | | | | | | 13.9 | | | | | | | | | | | | | | | | | | | | | | | | | | | | 6.2 | | | | | | | | | | ^33^ | | |
| % of female DCWs | **By Job Status** | | | | | | | | | | | | | **Permanent FT** | | | | | | | | | | | | | | | | | | | **Temporary/Casual FT** | | | | | | | | | | | | | | | | | | | | | | | | | | | | **Temporary/Casual PT** | | | | | | | | | |  | | |
|  |  | | | | | | | | | | | | | 76.0 | | | | | | | | | | | | | | | | | | | 85.5 | | | | | | | | | | | | | | | | | | | | | | | | | | | | 96.6 | | | | | | | | | | ^67^ | | |
|  |  | | | | | | | | | | | | |  | | | | | | | | | | | | | | | | | | | **Stayers** | | | | | | | | | | | | | | | | | | | | | | | | | | | | **Leavers** | | | | | | | | | |  | | |
|  |  | | | | | | | | | | | | |  | | | | | | | | | | | | | | | | | | | 79.8 | | | | | | | | | | | | | | | | | | | | | | | | | | | | 86.9 | | | | | | | | | | ^32^ | | |
|  |  | | | | | | | | | | | | |  | | | | | | | | | | | | | | | | | | | **CNA** | | | | | | | | | | | | | | | | | | | | | | | | | | | | **HHA** | | | | | | | | | |  | | |
|  |  | | | | | | | | | | | | |  | | | | | | | | | | | | | | | | | | | 92.1 | | | | | | | | | | | | | | | | | | | | | | | | | | | | 94.8* | | | | | | | | | | ^35^ | | |
| **Employment Characteristics**  % working full time | **DCWs Overall** | | | | | | | | | | | | | 69 | | | | | | | | | | | | | | | | | | |  | | | | | | | | | | | | | | | | | | | | | | | | | | | |  | | | | | | | | | | ^22^ | | |
|  |  | | | | | | | | | | | | | 62 | | | | | | | | | | | | | | | | | | |  | | | | | | | | | | | | | | | | | | | | | | | | | | | |  | | | | | | | | | | ^76^ | | |
|  |  | | | | | | | | | | | | | 14 (Alberta, 2009) | | | | | | | | | | | | | | | | | | |  | | | | | | | | | | | | | | | | | | | | | | | | | | | |  | | | | | | | | | | ^48^ | | |
|  | **By Setting** | | | | | | | | | | | | | **H** | | | | | | | | | | | | | | | | | | | **NH** | | | | | | | | | | | | | | | | | | | | | | | | | | | | **HH** | | | | | | | | | |  | | |
|  |  | | | | | | | | | | | | | 79.3 | | | | | | | | | | | | | | | | | | | 73.9 | | | | | | | | | | | | | | | | | | | | | | | | | | | | 55.1 | | | | | | | | | | ^81^ | | |
|  |  | | | | | | | | | | | | | 78.7 | | | | | | | | | | | | | | | | | | | 69.0 | | | | | | | | | | | | | | | | | | | | | | | | | | | | 65.2 | | | | | | | | | | ^39^ | | |
|  |  | | | | | | | | | | | | | 73 | | | | | | | | | | | | | | | | | | | 77 | | | | | | | | | | | | | | | | | | | | | | | | | | | | 61 | | | | | | | | | | ^22^ | | |
|  |  | | | | | | | | | | | | | 63.8 | | | | | | | | | | | | | | | | | | | 59.4 | | | | | | | | | | | | | | | | | | | | | | | | | | | | 53 | | | | | | | | | | ^17^ | | |
| % working part time | **DCWs Overall** | | | | | | | | | | | | | 46 (Alberta, 2009) | | | | | | | | | | | | | | | | | | |  | | | | | | | | | | | | | | | | | | | | | | | | | | | |  | | | | | | | | | | ^48^ | | |
|  |  | | | | | | | | | | | | | 40 (Casual, Alberta, 2009) | | | | | | | | | | | | | | | | | | | | | | | | | | | | | | | | | | | | | | | | | | | | | | |  | | | | | | | | | |  | | |
|  | **By Setting** | | | | | | | | | | | | | **H** | | | | | | | | | | | | | | | | | | | **NH** | | | | | | | | | | | | | | | | | | | | | | | | | | | | **HH** | | | | | | | | | |  | | |
|  |  | | | | | | | | | | | | |  | | | | | | | | | | | | | | | | | | | 78 | | | | | | | | | | | | | | | | | | | | | | | | | | | |  | | | | | | | | | | ^80^ | | |
|  |  | | | | | | | | | | | | |  | | | | | | | | | | | | | | | | | | | 13.1 | | | | | | | | | | | | | | | | | | | | | | | | | | | |  | | | | | | | | | | ^68^ | | |
|  |  | | | | | | | | | | | | |  | | | | | | | | | | | | | | | | | | |  | | | | | | | | | | | | | | | | | | | | | | | | | | | | 67.6 | | | | | | | | | | ^82^ | | |
|  |  | | | | | | | | | | | | |  | | | | | | | | | | | | | | | | | | | **NH** | | | | | | | | | | | | | | | | | | | | | | | | | | | | **Group home** | | | | | | | | | |  | | |
|  |  | | | | | | | | | | | | |  | | | | | | | | | | | | | | | | | | | 13.4 | | | | | | | | | | | | | | | | | | | | | | | | | | | | 11.2 | | | | | | | | | | ^68^ | | |
|  |  | | | | | | | | | | | | |  | | | | | | | | | | | | | | | | | | | **Geriatric inter-med. facility** | | | | | | | | | | | | | | | | | | | | | | | | | | | | | | | | | | | | | |  | | |
|  |  | | | | | | | | | | | | |  | | | | | | | | | | | | | | | | | | | 14.7 | | | | | | | | | | | | | | | | | | | | | | | | | | | |  | | | | | | | | | | ^68^ | | |
|  |  | | | | | | | | | | | | |  | | | | | | | | | | | | | | | | | | | **Hospice** | | | | | | | | | | | | | | | | | | | | | | | | | | | |  | | | | | | | | | | ^82^ | | |
|  |  | | | | | | | | | | | | |  | | | | | | | | | | | | | | | | | | | 27.6 | | | | | | | | | | | | | | | | | | | | | | | | | | | |  | | | | | | | | | |  | | |
| **Amount worked** | **DCWs Overall** | | | | | | | | | | | | | 44 | | | | | | | | | | | | | | | | | | |  | | | | | | | | | | | | | | | | | | | | | | | | | | | |  | | | | | | | | | | ^22^ | | |
| Mean weeks/year | **By Setting** | | | | | | | | | | | | | **H** | | | | | | | | | | | | | | | | | | | **NH** | | | | | | | | | | | | | | | | | | | | | | | | | | | | **HH** | | | | | | | | | |  | | |
|  |  | | | | | | | | | | | | | 47.5 | | | | | | | | | | | | | | | | | | | 45.7 | | | | | | | | | | | | | | | | | | | | | | | | | | | | 41.9 | | | | | | | | | | ^81^ | | |
|  |  | | | | | | | | | | | | | 46 | | | | | | | | | | | | | | | | | | | 44 | | | | | | | | | | | | | | | | | | | | | | | | | | | | 43 | | | | | | | | | | ^22^ | | |
|  |  | | | | | | | | | | | | | 44.0 | | | | | | | | | | | | | | | | | | | 42.5 | | | | | | | | | | | | | | | | | | | | | | | | | | | | 40.7 | | | | | | | | | | ^17^ | | |
| Mean hours/week  (SD or SE) | **DCWs Overall** | | | | | | | | | | | | | 37 | | | | | | | | | | | | | | | | | | |  | | | | | | | | | | | | | | | | | | | | | | | | | | | |  | | | | | | | | | | ^22^ | | |
|  |  | | | | | | | | | | | | | 24 | | | | | | | | | | | | | | | | | | |  | | | | | | | | | | | | | | | | | | | | | | | | | | | |  | | | | | | | | | | ^28^ | | |
|  | **By Setting** | | | | | | | | | | | | | **H** | | | | | | | | | | | | | | | | | | | **NH** | | | | | | | | | | | | | | | | | | | | | | | | | | | | **HH** | | | | | | | | | |  | | |
|  |  | | | | | | | | | | | | | 37 | | | | | | | | | | | | | | | | | | | 37.2 | | | | | | | | | | | | | | | | | | | | | | | | | | | | 37 | | | | | | | | | | ^17^ | | |
|  |  | | | | | | | | | | | | | 36 | | | | | | | | | | | | | | | | | | | 38 | | | | | | | | | | | | | | | | | | | | | | | | | | | | 36 | | | | | | | | | | ^22^ | | |
|  |  | | | | | | | | | | | | | 35.8 | | | | | | | | | | | | | | | | | | | 36.6 | | | | | | | | | | | | | | | | | | | | | | | | | | | | 34.7 | | | | | | | | | | ^81^ | | |
|  |  | | | | | | | | | | | | |  | | | | | | | | | | | | | | | | | | | 36.85 (0.23) | | | | | | | | | | | | | | | | | | | | | | | | | | | |  | | | | | | | | | | ^56^ | | |
|  |  | | | | | | | | | | | | |  | | | | | | | | | | | | | | | | | | | 37.22 (0.28) (tenure>1yr) | | | | | | | | | | | | | | | | | | | | | | | | | | | |  | | | | | | | | | |  | | |
|  |  | | | | | | | | | | | | |  | | | | | | | | | | | | | | | | | | | 36.81 | | | | | | | | | | | | | | | | | | | | | | | | | | | |  | | | | | | | | | | ^24^ | | |
|  |  | | | | | | | | | | | | |  | | | | | | | | | | | | | | | | | | | **Residential caregivers** | | | | | | | | | | | | | | | | | | | | | | | | | | | | **Home care** | | | | | | | | | |  | | |
|  |  | | | | | | | | | | | | |  | | | | | | | | | | | | | | | | | | | 25 | | | | | | | | | | | | | | | | | | | | | | | | | | | | 13 | | | | | | | | | | ^28^ | | |
|  | **By Immigration Status (NH)** | | | | | | | | | | | | | | | | | | | | | | | | | | | | | | | | **Immigrants** | | | | | | | | | | | | | | | | | | | | | | | | | | | | **Non-immigrants** | | | | | | | | | |  | | |
|  |  | | | | | | | | | | | | |  | | | | | | | | | | | | | | | | | | | 36.6 | | | | | | | | | | | | | | | | | | | | | | | | | | | | 37 | | | | | | | | | | ^33^ | | |
|  | **By Gender** | | | | | | | | | | | | |  | | | | | | | | | | | | | | | | | | | **Female** | | | | | | | | | | | | | | | | | | | | | | | | | | | | **Male** | | | | | | | | | |  | | |
|  |  | | | | | | | | | | | | |  | | | | | | | | | | | | | | | | | | | 36.4 | | | | | | | | | | | | | | | | | | | | | | | | | | | | 36.5 | | | | | | | | | | ^52^ | | |
| Mean overtime shifts/week for HCA staff  (SD or SE) | **DCWs Overall** | | | | | | | | | | | | | 9.71 (19.10) | | | | | | | | | | | | | | | | | | |  | | | | | | | | | | | | | | | | | | | | | | | | | | | |  | | | | | | | | | | ^57^ | | |
|  |  | | | | | | | | | | | | | 10.1 (19.8) | | | | | | | | | | | | | | | | | | |  | | | | | | | | | | | | | | | | | | | | | | | | | | | |  | | | | | | | | | | ^58^ | | |
|  | **By Immigration status (NH)** | | | | | | | | | | | | | | | | | | | | | | | | | | | | | | | | **Immigrant** | | | | | | | | | | | | | | | | | | | | | | | | | | | | **Non-immigrant** | | | | | | | | | |  | | |
|  | (mandatory) | | | | | | | | | | | | |  | | | | | | | | | | | | | | | | | | | 18.1 | | | | | | | | | | | | | | | | | | | | | | | | | | | | 22.8 | | | | | | | | | | ^33^ | | |
| % working overtime | **By Job status** | | | | | | | | | | | | | **Permanent FT** | | | | | | | | | | | | | | | | | | | **Precarious FT** | | | | | | | | | | | | | | | | | | | | | | | | | | | | **Precarious PT** | | | | | | | | | |  | | |
|  | <10 | | | | | | | | | | | | | 34.4 | | | | | | | | | | | | | | | | | | | 31.9 | | | | | | | | | | | | | | | | | | | | | | | | | | | | 10.5 | | | | | | | | | | ^67^ | | |
|  | ≥10 | | | | | | | | | | | | | 6.7 | | | | | | | | | | | | | | | | | | | 3.0 | | | | | | | | | | | | | | | | | | | | | | | | | | | | 0.8 | | | | | | | | | |  | | |
|  | No overtime work | | | | | | | | | | | | | 54.7 | | | | | | | | | | | | | | | | | | | 31.6 | | | | | | | | | | | | | | | | | | | | | | | | | | | | 84.5 | | | | | | | | | |  | | |
| % working shift | Mainly Day | | | | | | | | | | | | | 61 (NH) | | | | | | | | | | | | | | | | | | |  | | | | | | | | | | | | | | | | | | | | | | | | | | | |  | | | | | | | | | | ^80^ | | |
|  |  | | | | | | | | | | | | | 43.4 | | | | | | | | | | | | | | | | | | |  | | | | | | | | | | | | | | | | | | | | | | | | | | | |  | | | | | | | | | | ^66^ | | |
|  | Mainly Evening | | | | | | | | | | | | | 24.8 | | | | | | | | | | | | | | | | | | |  | | | | | | | | | | | | | | | | | | | | | | | | | | | |  | | | | | | | | | |  | | |
|  |  | | | | | | | | | | | | | 22 (NH) | | | | | | | | | | | | | | | | | | |  | | | | | | | | | | | | | | | | | | | | | | | | | | | |  | | | | | | | | | | ^80^ | | |
|  | Mainly Night | | | | | | | | | | | | | 17 (NH) | | | | | | | | | | | | | | | | | | |  | | | | | | | | | | | | | | | | | | | | | | | | | | | |  | | | | | | | | | |  | | |
|  |  | | | | | | | | | | | | | 10.5 | | | | | | | | | | | | | | | | | | |  | | | | | | | | | | | | | | | | | | | | | | | | | | | |  | | | | | | | | | | ^66^ | | |
|  | Mixed | | | | | | | | | | | | | 21.3 | | | | | | | | | | | | | | | | | | |  | | | | | | | | | | | | | | | | | | | | | | | | | | | |  | | | | | | | | | |  | | |
|  | **By Job status** | | | | | | | | | | | | | **Permanent FT** | | | | | | | | | | | | | | | | | | | **Precarious FT** | | | | | | | | | | | | | | | | | | | | | | | | | | | | **Precarious PT** | | | | | | | | | |  | | |
|  | Mainly Night | | | | | | | | | | | | | 33.5 | | | | | | | | | | | | | | | | | | | 23.4 | | | | | | | | | | | | | | | | | | | | | | | | | | | | 4.1 | | | | | | | | | | ^67^ | | |
| **Wage**  Mean US dollars unless otherwise stated  (SD or SE) | **DCWs Overall** | | | | | | | | | | | | |  | | | | | | | | | | | | | | | | | | |  | | | | | | | | | | | | | | | | | | | | | | | | | | | |  | | | | | | | | | |  | | |
|  | $14.56 | | | | | | | | | | | | |  | | | | | | | | | | | | | | | | | | |  | | | | | | | | | | | | | | | | | | | | | | | | | | | |  | | | | | | | | | | ^22^ | | |
|  | $11.47 ($6-14) | | | | | | | | | | | | |  | | | | | | | | | | | | | | | | | | |  | | | | | | | | | | | | | | | | | | | | | | | | | | | |  | | | | | | | | | | ^40^ | | |
|  | $10.82 | | | | | | | | | | | | |  | | | | | | | | | | | | | | | | | | |  | | | | | | | | | | | | | | | | | | | | | | | | | | | |  | | | | | | | | | | ^72^ | | |
|  | $10.36 (Median $8.83) | | | | | | | | | | | | | | | | | | | | | | | | | | | | | | | |  | | | | | | | | | | | | | | | | | | | | | | | | | | | |  | | | | | | | | | | ^77^ | | |
|  | $10.31 (12.13) | | | | | | | | | | | | |  | | | | | | | | | | | | | | | | | | |  | | | | | | | | | | | | | | | | | | | | | | | | | | | |  | | | | | | | | | | ^72^ | | |
|  | $10.30 (0.10) | | | | | | | | | | | | |  | | | | | | | | | | | | | | | | | | |  | | | | | | | | | | | | | | | | | | | | | | | | | | | |  | | | | | | | | | | ^49^ | | |
|  | $8.21 ( Median, US, 2000) | | | | | | | | | | | | | | | | | | | | | | | | | | | | | | | |  | | | | | | | | | | | | | | | | | | | | | | | | | | | |  | | | | | | | | | | ^84^ | | |
|  | $8.19 (1.158) | | | | | | | | | | | | |  | | | | | | | | | | | | | | | | | | |  | | | | | | | | | | | | | | | | | | | | | | | | | | | |  | | | | | | | | | | ^85^ | | |
|  | $7.86 (Median, North Carolina, 2000) | | | | | | | | | | | | | | | | | | | | | | | | | | | | | | | |  | | | | | | | | | | | | | | | | | | | | | | | | | | | |  | | | | | | | | | | ^84^ | | |
|  | **By Setting** | | | | | | | | | | | | | **H** | | | | | | | | | | | | | | | | | | | **NH** | | | | | | | | | | | | | | | | | | | | | | | | | | | | **HH** | | | | | | | | | |  | | |
|  |  | | | | | | | | | | | | | $7.94 | | | | | | | | | | | | | | | | | | | $5.60 | | | | | | | | | | | | | | | | | | | | | | | | | | | | $5.25 | | | | | | | | | | ^81^ | | |
|  |  | | | | | | | | | | | | | $7.99 | | | | | | | | | | | | | | | | | | | $7.45 | | | | | | | | | | | | | | | | | | | | | | | | | | | | $7.45 | | | | | | | | | | ^39^ | | |
|  |  | | | | | | | | | | | | | $14.44 | | | | | | | | | | | | | | | | | | | $11.46 | | | | | | | | | | | | | | | | | | | | | | | | | | | | $13.38 | | | | | | | | | | ^17^ | | |
|  |  | | | | | | | | | | | | | $12.06 | | | | | | | | | | | | | | | | | | | $12.20 | | | | | | | | | | | | | | | | | | | | | | | | | | | | $17.84 | | | | | | | | | | ^22^ | | |
|  |  | | | | | | | | | | | | |  | | | | | | | | | | | | | | | | | | | $8.70 (1.5) | | | | | | | | | | | | | | | | | | | | | | | | | | | |  | | | | | | | | | | ^58^ | | |
|  |  | | | | | | | | | | | | |  | | | | | | | | | | | | | | | | | | | $8.72 (starting wage, SD = 1.68) | | | | | | | | | | | | | | | | | | | | | | | | | | | | ^55^ | | | | | | | | | |  | | |
|  |  | | | | | | | | | | | | |  | | | | | | | | | | | | | | | | | | | $10.40 | | | | | | | | | | | | | | | | | | | | | | | | | | | |  | | | | | | | | | | ^33^ | | |
|  |  | | | | | | | | | | | | |  | | | | | | | | | | | | | | | | | | | $10.34 (0.1) | | | | | | | | | | | | | | | | | | | | | | | | | | | |  | | | | | | | | | | ^53^ | | |
|  |  | | | | | | | | | | | | |  | | | | | | | | | | | | | | | | | | | $10.33 | | | | | | | | | | | | | | | | | | | | | | | | | | | |  | | | | | | | | | | ^24^ | | |
|  |  | | | | | | | | | | | | |  | | | | | | | | | | | | | | | | | | |  | | | | | | | | | | | | | | | | | | | | | | | | | | | | $15.48 (CAN) | | | | | | | | | | ^41^ | | |
|  |  | | | | | | | | | | | | |  | | | | | | | | | | | | | | | | | | |  | | | | | | | | | | | | | | | | | | | | | | | | | | | | $10.00-$19.87(CAN) | | | | | | | | | |  | | |
|  |  | | | | | | | | | | | | |  | | | | | | | | | | | | | | | | | | | **Not for profit NH** | | | | | | | | | | | | | | | | | | | | | | | | | | | | **For Profit NH** | | | | | | | | | |  | | |
|  |  | | | | | | | | | | | | |  | | | | | | | | | | | | | | | | | | | $8.16 | | | | | | | | | | | | | | | | | | | | | | | | | | | | $8.20 | | | | | | | | | | ^87^ | | |
|  |  | | | | | | | | | | | | |  | | | | | | | | | | | | | | | | | | | **Residential** | | | | | | | | | | | | | | | | | | | | | | | | | | | | **Home care** | | | | | | | | | |  | | |
|  |  | | | | | | | | | | | | |  | | | | | | | | | | | | | | | | | | | $10.80 (NZ) | | | | | | | | | | | | | | | | | | | | | | | | | | | | $11.00(NZ) | | | | | | | | | | ^28^ | | |
|  | **By Immigration Status (NH)** | | | | | | | | | | | | | | | | | | | | | | | | | | | | | | | | **Immigrants** | | | | | | | | | | | | | | | | | | | | | | | | | | | | **Non-Immigrants** | | | | | | | | | |  | | |
|  |  | | | | | | | | | | | | |  | | | | | | | | | | | | | | | | | | | $11.20 | | | | | | | | | | | | | | | | | | | | | | | | | | | | $10.20 | | | | | | | | | | ^33^ | | |
|  | **By Gender** | | | | | | | | | | | | |  | | | | | | | | | | | | | | | | | | | **Females** | | | | | | | | | | | | | | | | | | | | | | | | | | | | **Males** | | | | | | | | | |  | | |
|  |  | | | | | | | | | | | | |  | | | | | | | | | | | | | | | | | | | $9.60 ( 2.0) | | | | | | | | | | | | | | | | | | | | | | | | | | | | $9.90 ( 2.2) | | | | | | | | | | ^52^ | | |
| Household Income  % by household income level |  | | | | | | | | | | | | | **DCWs Overall** | | | | | | | | | | | | | | | | | | | **NH** | | | | | | | | | | | | | | | | | | | | | | | | | | | | **HH** | | | | | | | | | |  | | |
|  | <$30,000 | | | | | | | | | | | | | 66.8 | | | | | | | | | | | | | | | | | | |  | | | | | | | | | | | | | | | | | | | | | | | | | | | |  | | | | | | | | | | ^33^ | | |
|  |  | | | | | | | | | | | | | 63 | | | | | | | | | | | | | | | | | | |  | | | | | | | | | | | | | | | | | | | | | | | | | | | |  | | | | | | | | | | ^49^ | | |
|  |  | | | | | | | | | | | | | 65.4 | | | | | | | | | | | | | | | | | | |  | | | | | | | | | | | | | | | | | | | | | | | | | | | | 49.6 | | | | | | | | | | ^33^ | | |
|  |  | | | | | | | | | | | | |  | | | | | | | | | | | | | | | | | | | 63.4 | | | | | | | | | | | | | | | | | | | | | | | | | | | |  | | | | | | | | | | ^22^ | | |
|  | ≥$30,000 | | | | | | | | | | | | |  | | | | | | | | | | | | | | | | | | | 32.6 | | | | | | | | | | | | | | | | | | | | | | | | | | | |  | | | | | | | | | |  | | |
|  | **By Gender** | | | | | | | | | | | | |  | | | | | | | | | | | | | | | | | | | **Female** | | | | | | | | | | | | | | | | | | | | | | | | | | | | **Male** | | | | | | | | | |  | | |
|  | <$30,000 | | | | | | | | | | | | |  | | | | | | | | | | | | | | | | | | | 70.3 | | | | | | | | | | | | | | | | | | | | | | | | | | | | 63.3 | | | | | | | | | | ^52^ | | |
|  | ≥$30,000 | | | | | | | | | | | | |  | | | | | | | | | | | | | | | | | | | 29.7 | | | | | | | | | | | | | | | | | | | | | | | | | | | | 36.6 | | | | | | | | | |  | | |
|  | **By Immigration Status (NH)** | | | | | | | | | | | | | | | | | | | | | | | | | | | | | | | | **Immigrant** | | | | | | | | | | | | | | | | | | | | | | | | | | | | **Non-immigrant** | | | | | | | | | |  | | |
|  | <$30,000 | | | | | | | | | | | | |  | | | | | | | | | | | | | | | | | | | 65.0 | | | | | | | | | | | | | | | | | | | | | | | | | | | | 67.2 | | | | | | | | | | ^33^ | | |
| % below federal poverty level |  | | | | | | | | | | | | | | | | | | | | | | | | | | | | | | | | **DCWs** | | | | | | | | | | | | | | | | | | | | | | | | | | | | **Other Workers** | | | | | | | | | |  | | |
|  | Family income is ≤ 150% of federal poverty level | | | | | | | | | | | | | | | | | | | | | | | | | | | | | | | | 33 | | | | | | | | | | | | | | | | | | | | | | | | | | | | 13 | | | | | | | | | | ^76^ | | |
|  |  | | | | | | | | | | | | | | | | | | | | | | | | | | | | | | | | 18 | | | | | | | | | | | | | | | | | | | | | | | | | | | | 11 | | | | | | | | | | ^84^ | | |
|  | In Poverty | | | | | | | | | | | | | | | | | | | | | | | | | | | | | | | | 19 | | | | | | | | | | | | | | | | | | | | | | | | | | | |  | | | | | | | | | | ^22^ | | |
|  | Low Income | | | | | | | | | | | | | | | | | | | | | | | | | | | | | | | | 49 | | | | | | | | | | | | | | | | | | | | | | | | | | | |  | | | | | | | | | |  | | |
|  | **By Setting** | | | | | | | | | | | | | **H** | | | | | | | | | | | | | | | | | | | **NH** | | | | | | | | | | | | | | | | | | | | | | | | | | | | **HH** | | | | | | | | | |  | | |
|  | In Poverty | | | | | | | | | | | | | 10 | | | | | | | | | | | | | | | | | | | 18 | | | | | | | | | | | | | | | | | | | | | | | | | | | | 23 | | | | | | | | | | ^22^ | | |
|  | Low Income | | | | | | | | | | | | | 34 | | | | | | | | | | | | | | | | | | | 52 | | | | | | | | | | | | | | | | | | | | | | | | | | | | 51 | | | | | | | | | |  | | |
| % by Income to Poverty ratio | **By Setting** | | | | | | | | | | | | | **H** | | | | | | | | | | | | | | | | | | | **NH** | | | | | | | | | | | | | | | | | | | | | | | | | | | | **HH** | | | | | | | | | |  | | |
|  | <1.00 | | | | | | | | | | | | | 9.2 | | | | | | | | | | | | | | | | | | | 16.1 | | | | | | | | | | | | | | | | | | | | | | | | | | | | 22.2 | | | | | | | | | | ^39^ | | |
|  |  | | | | | | | | | | | | | 11.4 | | | | | | | | | | | | | | | | | | | 16.4 | | | | | | | | | | | | | | | | | | | | | | | | | | | | 19.3 | | | | | | | | | | ^17^ | | |
|  | 1.00-1.49 | | | | | | | | | | | | | 10.0 | | | | | | | | | | | | | | | | | | | 13.4 | | | | | | | | | | | | | | | | | | | | | | | | | | | | 15.7 | | | | | | | | | | ^39^ | | |
|  |  | | | | | | | | | | | | | 10.1 | | | | | | | | | | | | | | | | | | | 14.2 | | | | | | | | | | | | | | | | | | | | | | | | | | | | 14.1 | | | | | | | | | | ^17^ | | |
|  | 1.50-1.99 | | | | | | | | | | | | | 11.4 | | | | | | | | | | | | | | | | | | | 16.0 | | | | | | | | | | | | | | | | | | | | | | | | | | | | 9.3 | | | | | | | | | | ^39^ | | |
|  |  | | | | | | | | | | | | | 11.6 | | | | | | | | | | | | | | | | | | | 14.3 | | | | | | | | | | | | | | | | | | | | | | | | | | | | 13.9 | | | | | | | | | | ^17^ | | |
|  | 2.00-2.49 | | | | | | | | | | | | | 12.6 | | | | | | | | | | | | | | | | | | | 14.2 | | | | | | | | | | | | | | | | | | | | | | | | | | | | 8.8 | | | | | | | | | | ^39^ | | |
|  |  | | | | | | | | | | | | | 12.4 | | | | | | | | | | | | | | | | | | | 12.9 | | | | | | | | | | | | | | | | | | | | | | | | | | | | 11.3 | | | | | | | | | | ^17^ | | |
|  | 2.50-2.99 | | | | | | | | | | | | | 11.3 | | | | | | | | | | | | | | | | | | | 11.1 | | | | | | | | | | | | | | | | | | | | | | | | | | | | 12.5 | | | | | | | | | | ^39^ | | |
|  |  | | | | | | | | | | | | | 10.9 | | | | | | | | | | | | | | | | | | | 10.4 | | | | | | | | | | | | | | | | | | | | | | | | | | | | 9.5 | | | | | | | | | | ^17^ | | |
|  | 3.00+ | | | | | | | | | | | | | 45.5 | | | | | | | | | | | | | | | | | | | 29.3 | | | | | | | | | | | | | | | | | | | | | | | | | | | | 31.5 | | | | | | | | | | ^39^ | | |
|  |  | | | | | | | | | | | | | 43.7 | | | | | | | | | | | | | | | | | | | 31.9 | | | | | | | | | | | | | | | | | | | | | | | | | | | | 31.8 | | | | | | | | | | ^17^ | | |
| % by main income earner | **By Job status** | | | | | | | | | | | | | **Permanent FT** | | | | | | | | | | | | | | | | | | | **Precarious FT** | | | | | | | | | | | | | | | | | | | | | | | | | | | | **Precarious PT** | | | | | | | | | |  | | |
|  | Myself | | | | | | | | | | | | | 40.4 | | | | | | | | | | | | | | | | | | | 30.6 | | | | | | | | | | | | | | | | | | | | | | | | | | | | 17.5 | | | | | | | | | | ^67^ | | |
|  | Other family members | | | | | | | | | | | | | 58.2 | | | | | | | | | | | | | | | | | | | 68.2 | | | | | | | | | | | | | | | | | | | | | | | | | | | | 81.5 | | | | | | | | | |  | | |
|  | Data missing | | | | | | | | | | | | | 1.5 | | | | | | | | | | | | | | | | | | | 1.2 | | | | | | | | | | | | | | | | | | | | | | | | | | | | 1.0 | | | | | | | | | |  | | |
| **Federal Assistance**  % using listed federal assistance program  (SD or SE) | “Nursing aides working home care and nursing homes are twice as likely as workers in other occupations to receive public benefits, particularly food stamps and/or Medicaid-covered health benefits” | | | | | | | | | | | | | | | | | | | | | | | | | | | | | | | | | | | | | | | | | | | | | | | | | | | | | | | | | | | | | | | | | | | | | | ^84^ | | |
|  | **Benefit reported** | | | | | | | | | | | | |  | | | | | | | | | | | | | | | | | | | **DCWs** | | | | | | | | | | | | | | | | | | | | | | | | | | | | **Other Workers** | | | | | | | | | |  | | |
|  | Receives public assistance | | | | | | | | | | | | | | | | | | | | | | | | | | | | | | | | 5 | | | | | | | | | | | | | | | | | | | | | | | | | | | | 1 | | | | | | | | | | ^76^ | | |
|  |  | | | | | | | | | | | | | | | | | | | | | | | | | | | | | | | | 31 (0.01) (NH) | | | | | | | | | | | | | | | | | | | | | | | | | | | |  | | | | | | | | | | ^56^ | | |
|  |  | | | | | | | | | | | | | | | | | | | | | | | | | | | | | | | | 31.4 (NH) | | | | | | | | | | | | | | | | | | | | | | | | | | | |  | | | | | | | | | | ^33^ | | |
|  |  | | | | | | | | | | | | | | | | | | | | | | | | | | | | | | | | 30.37 (1.16) (NH) | | | | | | | | | | | | | | | | | | | | | | | | | | | |  | | | | | | | | | | ^24^ | | |
|  | Ever received food stamps | | | | | | | | | | | | | | | | | | | | | | | | | | | | | | | | 42.74 (1.43)(NH) | | | | | | | | | | | | | | | | | | | | | | | | | | | |  | | | | | | | | | | ^24^ | | |
|  | Receives food stamps (1999) | | | | | | | | | | | | | | | | | | | | | | | | | | | | | | | | 14 | | | | | | | | | | | | | | | | | | | | | | | | | | | | 5.5 | | | | | | | | | | ^84^ | | |
|  | Receives food stamps | | | | | | | | | | | | | | | | | | | | | | | | | | | | | | | | 10.78 (0.80) (NH) | | | | | | | | | | | | | | | | | | | | | | | | | | | |  | | | | | | | | | | ^24^ | | |
|  | Ever received WIC (Special Supplemental Nutrition Program for Women, Infants and Children) | | | | | | | | | | | | | | | | | | | | | | | | | | | | | | | | 42.73(1.27)(NH) | | | | | | | | | | | | | | | | | | | | | | | | | | | |  | | | | | | | | | |  | | |
|  | Receives WIC | | | | | | | | | | | | | | | | | | | | | | | | | | | | | | | | 7 | | | | | | | | | | | | | | | | | | | | | | | | | | | | 2 | | | | | | | | | | ^76^ | | |
|  |  | | | | | | | | | | | | | | | | | | | | | | | | | | | | | | | | 8.99 (0.66)(NH) | | | | | | | | | | | | | | | | | | | | | | | | | | | |  | | | | | | | | | | ^24^ | | |
|  | Ever received TANF (Temporary Assistance for Needy Families) | | | | | | | | | | | | | | | | | | | | | | | | | | | | | | | | 23.44 (1.14)(NH) | | | | | | | | | | | | | | | | | | | | | | | | | | | |  | | | | | | | | | |  | | |
|  | Receives Medicaid (1999) | | | | | | | | | | | | | | | | | | | | | | | | | | | | | | | | 10 | | | | | | | | | | | | | | | | | | | | | | | | | | | | 4 | | | | | | | | | | ^74^ | | |
|  |  | | | | | | | | | | | | | | | | | | | | | | | | | | | | | | | | 23.06 (1.10)(NH) | | | | | | | | | | | | | | | | | | | | | | | | | | | |  | | | | | | | | | | ^24^ | | |
|  | Receives financial, housing, or energy assistance | | | | | | | | | | | | | | | | | | | | | | | | | | | | | | | | 7 | | | | | | | | | | | | | | | | | | | | | | | | | | | | 3 | | | | | | | | | | ^76^ | | |
|  | Lives in public housing or receives rental subsidy | | | | | | | | | | | | | | | | | | | | | | | | | | | | | | | | 7.48 (0.73)(NH) | | | | | | | | | | | | | | | | | | | | | | | | | | | |  | | | | | | | | | | ^24^ | | |
|  | **By Immigration Status (NH)** | | | | | | | | | | | | | | | | | | | | | | | | | | | | | | | | **Immigrant** | | | | | | | | | | | | | | | | | | | | | | | | | | | | **Non-immigrant** | | | | | | | | | |  | | |
|  |  | | | | | | | | | | | | | | | | | | | | | | | | | | | | | | | | 28.4 | | | | | | | | | | | | | | | | | | | | | | | | | | | | 31.9 | | | | | | | | | | ^33^ | | |
| **Previous/ Current Jobs**  Mean number of jobs (SD or SE) | **DCWs Overall** | | | | | | | | | | | | |  | | | | | | | | | | | | | | | | | | |  | | | | | | | | | | | | | | | | | | | | | | | | | | | |  | | | | | | | | | |  | | |
|  | # prior jobs as NA | | | | | | | | | | | | | 3.5 (0.8) | | | | | | | | | | | | | | | | | | |  | | | | | | | | | | | | | | | | | | | | | | | | | | | |  | | | | | | | | | | ^83^ | | |
|  | # prior jobs | | | | | | | | | | | | | 5.5 (1.5) | | | | | | | | | | | | | | | | | | |  | | | | | | | | | | | | | | | | | | | | | | | | | | | |  | | | | | | | | | | ^80^ | | |
|  | # of current jobs | | | | | | | | | | | | | “Workers in New Brunswick have expressed that without regular or guaranteed hours of employment in **home care**, a second job is often necessary to achieve adequate income” | | | | | | | | | | | | | | | | | | | | | | | | | | | | | | | | | | | | | | | | | | | | | | | | | | | | | | | | | ^44^ | | |
| % with multiple jobs | **DCWs Overall** | | | | | | | | | | | | | 11.36 | | | | | | | | | | | | | | | | | | |  | | | | | | | | | | | | | | | | | | | | | | | | | | | |  | | | | | | | | | | ^72^ | | |
|  | **By Job Status** | | | | | | | | | | | | | **Permanent FT** | | | | | | | | | | | | | | | | | | | **Precarious FT** | | | | | | | | | | | | | | | | | | | | | | | | | | | | **Precarious PT** | | | | | | | | | |  | | |
|  |  | | | | | | | | | | | | | 5.8 | | | | | | | | | | | | | | | | | | | 6.2 | | | | | | | | | | | | | | | | | | | | | | | | | | | | 15.4 | | | | | | | | | | ^67^ | | |
| **USE** | | | | | | | | | | | | | | | | | | | | | | | | | | | | | | | | | | | | | | | | | | | | | | | | | | | | | | | | | | | | | | | | | | | | | | | | | |
| **Themes** | **Outcomes** | | | | | | | | | | | | | | | | | | |  | | | | | | | | | | | | | | | | | | | | | | | | | | | | | | | | | | | | | | | | | | | | | | | | | | | **Ref.** | | |
| **Role of HCA** | Independent | | | | | | | | | | | | | | | | | | |  | | | | | | | | | | | | | | | | | | | | | | | | | | | | | | | | | | | | | | | | | | | | | | | | | | | ^42,45^ | | |
|  | Issues around independence | | | | | | | | | | | | | | | | | | |  | | | | | | | | | | | | | | | | | | | | | | | | | | | | | | | | | | | | | | | | | | | | | | | | | | | ^46^ | | |
|  | Dependent | | | | | | | | | | | | | | | | | | |  | | | | | | | | | | | | | | | | | | | | | | | | | | | | | | | | | | | | | | | | | | | | | | | | | | | ^45^ | | |
|  | Multidisciplinary working | | | | | | | | | | | | | | | | | | |  | | | | | | | | | | | | | | | | | | | | | | | | | | | | | | | | | | | | | | | | | | | | | | | | | | | ^18^ | | |
|  | Supportive & Assistive | | | | | | | | | | | | | | | | | | |  | | | | | | | | | | | | | | | | | | | | | | | | | | | | | | | | | | | | | | | | | | | | | | | | | | | ^29^ | | |
| **Models of Supervision** | Direct (formal or informal) | | | | | | | | | | | | | | | | | | |  | | | | | | | | | | | | | | | | | | | | | | | | | | | | | | | | | | | | | | | | | | | | | | | | | | | ^18, 42^ | | |
|  | Team supervision | | | | | | | | | | | | | | | | | | |  | | | | | | | | | | | | | | | | | | | | | | | | | | | | | | | | | | | | | | | | | | | | | | | | | | | ^18^ | | |
|  | Mentored | | | | | | | | | | | | | | | | | | |  | | | | | | | | | | | | | | | | | | | | | | | | | | | | | | | | | | | | | | | | | | | | | | | | | | |  | | |
| **Description of Role** | Patient Contact Tasks | | | | | | | | | | | | | | | | | | | Provide personal care general description  (Indirect & Direct) | | | | | | | | | | | | | | | | | | | | | | | | | | | | | | | | | | | | | | | | | | | | | | | | | | | ^18,42,^ ^40.45^ | | |
|  |  | | | | | | | | | | | | | | | | | | | Bathing  Clinical observations or vital sign recording | | | | | | | | | | | | | | | | | | | | | | | | | | | | | | | | | | | | | | | | | | | | | | | | | | | ^47^ | | |
|  |  | | | | | | | | | | | | | | | | | | | Feeding | | | | | | | | | | | | | | | | | | | | | | | | | | | | | | | | | | | | | | | | | | | | | | | | | | | ^40,45,47^ | | |
|  |  | | | | | | | | | | | | | | | | | | | Focus on enablement  Meet rehabilitation needs | | | | | | | | | | | | | | | | | | | | | | | | | | | | | | | | | | | | | | | | | | | | | | | | | | | ^18^ | | |
|  |  | | | | | | | | | | | | | | | | | | | Oral care | | | | | | | | | | | | | | | | | | | | | | | | | | | | | | | | | | | | | | | | | | | | | | | | | | | ^40,45^ | | |
|  |  | | | | | | | | | | | | | | | | | | | Shaving | | | | | | | | | | | | | | | | | | | | | | | | | | | | | | | | | | | | | | | | | | | | | | | | | | | ^45^ | | |
|  | Physical Tasks | | | | | | | | | | | | | | | | | | | Accompany older adults to doctors | | | | | | | | | | | | | | | | | | | | | | | | | | | | | | | | | | | | | | | | | | | | | | | | | | | ^40^ | | |
|  |  | | | | | | | | | | | | | | | | | | | Moving patients in bed  Supporting patients between bed and chair  Handling heavy objects at work | | | | | | | | | | | | | | | | | | | | | | | | | | | | | | | | | | | | | | | | | | | | | | | | | | | ^70^ | | |
|  | Clerical Tasks/ Administrative Duties | | | | | | | | | | | | | | | | | | | Generally | | | | | | | | | | | | | | | | | | | | | | | | | | | | | | | | | | | | | | | | | | | | | | | | | | | ^42,45,47^ | | |
|  |  | | | | | | | | | | | | | | | | | | | Schedule physician appointments | | | | | | | | | | | | | | | | | | | | | | | | | | | | | | | | | | | | | | | | | | | | | | | | | | | ^40^ | | |
|  |  | | | | | | | | | | | | | | | | | | | Pay bills or handle patient money | | | | | | | | | | | | | | | | | | | | | | | | | | | | | | | | | | | | | | | | | | | | | | | | | | |  | | |
|  | Non Patient Contact Tasks | | | | | | | | | | | | | | | | | | | Maintain care environment | | | | | | | | | | | | | | | | | | | | | | | | | | | | | | | | | | | | | | | | | | | | | | | | | | | ^42^ | | |
|  |  | | | | | | | | | | | | | | | | | | | Stocking or preparation of work environment | | | | | | | | | | | | | | | | | | | | | | | | | | | | | | | | | | | | | | | | | | | | | | | | | | | ^47^ | | |
|  |  | | | | | | | | | | | | | | | | | | | Laundry | | | | | | | | | | | | | | | | | | | | | | | | | | | | | | | | | | | | | | | | | | | | | | | | | | |  | | |
|  |  | | | | | | | | | | | | | | | | | | | Housekeeping | | | | | | | | | | | | | | | | | | | | | | | | | | | | | | | | | | | | | | | | | | | | | | | | | | | ^40,47^ | | |
|  | Similar tasks to RN | | | | | | | | | | | | | | | | | | | Generally described as similar | | | | | | | | | | | | | | | | | | | | | | | | | | | | | | | | | | | | | | | | | | | | | | | | | | | ^29^ | | |
|  |  | | | | | | | | | | | | | | | | | | | Administer medications | | | | | | | | | | | | | | | | | | | | | | | | | | | | | | | | | | | | | | | | | | | | | | | | | | | ^42,47^ | | |
|  |  | | | | | | | | | | | | | | | | | | | Catheterization | | | | | | | | | | | | | | | | | | | | | | | | | | | | | | | | | | | | | | | | | | | | | | | | | | | ^42,47^ | | |
|  |  | | | | | | | | | | | | | | | | | | | Could perform CPR and First aid | | | | | | | | | | | | | | | | | | | | | | | | | | | | | | | | | | | | | | | | | | | | | | | | | | | ^40^ | | |
|  |  | | | | | | | | | | | | | | | | | | | Ear syringing, ECG, Venipuncture | | | | | | | | | | | | | | | | | | | | | | | | | | | | | | | | | | | | | | | | | | | | | | | | | | | ^42^ | | |
|  |  | | | | | | | | | | | | | | | | | | | Handling syringes, Blood samples, Securing Endotracheal Tubes (ET), acting as scrub nurse, Suctioning ET tubes, Cardiac Arrest Massage, Blood Glucose Monitoring, Removal of IVs, Removal of Catheters, Cardiac Monitoring, Administration of Enemas and Suppositories, Neurological Observations, Percutaneous Gastrotomy Tube Feeding, Troponin Testing, Pregnancy Testing, Application of Plaster of Paris, Artificial Ventilation and Wound Care | | | | | | | | | | | | | | | | | | | | | | | | | | | | | | | | | | | | | | | | | | | | | | | | | | | ^47^ | | |
| **Factors associated with Role** | Need for role clarification | | | | | | | | | | | | | | | | | | | Lack of consensus or knowledge about the HCA role | | | | | | | | | | | | | | | | | | | | | | | | | | | | | | | | | | | | | | | | | | | | | | | | | | | ^29,42,45-47^ | | |
|  | Hierarchy of role | | | | | | | | | | | | | | | | | | |  | | | | | | | | | | | | | | | | | | | | | | | | | | | | | | | | | | | | | | | | | | | | | | | | | | | ^42,45,46^ | | |
|  | Boundary Crossing | | | | | | | | | | | | | | | | | | | With RNs or uncertain boundary between roles reported | | | | | | | | | | | | | | | | | | | | | | | | | | | | | | | | | | | | | | | | | | | | | | | | | | | ^29,42,45,47^ | | |
|  | Effect on RN | | | | | | | | | | | | | | | | | | | RN risks losing contact with patients  RN fear of job or role of RN lost to HCA | | | | | | | | | | | | | | | | | | | | | | | | | | | | | | | | | | | | | | | | | | | | | | | | | | | ^42^  ^46^ | | |
|  | Effect on Other Staff | | | | | | | | | | | | | | | | | | | Decreases staff morale | | | | | | | | | | | | | | | | | | | | | | | | | | | | | | | | | | | | | | | | | | | | | | | | | | | ^45^ | | |
|  | Effect on Patients | | | | | | | | | | | | | | | | | | | Improved quality of care | | | | | | | | | | | | | | | | | | | | | | | | | | | | | | | | | | | | | | | | | | | | | | | | | | | ^42,45^ | | |
|  |  | | | | | | | | | | | | | | | | | | | Improved patient satisfaction | | | | | | | | | | | | | | | | | | | | | | | | | | | | | | | | | | | | | | | | | | | | | | | | | | | ^45^ | | |
|  |  | | | | | | | | | | | | | | | | | | | Increased appropriate care | | | | | | | | | | | | | | | | | | | | | | | | | | | | | | | | | | | | | | | | | | | | | | | | | | |  | | |
|  |  | | | | | | | | | | | | | | | | | | | Increased length of stay | | | | | | | | | | | | | | | | | | | | | | | | | | | | | | | | | | | | | | | | | | | | | | | | | | |  | | |
| **Skill Mix**  Mean  qualified staff : SW  Staffing ratio |  | | | | | | | | | | | | | | | | | | | | | | | | | | | | | | | | | | | | | | | | | | | | | | | | | | | | | | | | | | | | | | | | | | | | | |  | | |
|  | 0.95 (0-4.09qualified staff for every SW) | | | | | | | | | | | | | | | | | | | | | | | | | | | | | | | | | | | | | | | | | | | | | | | | | | | | | | | | | | | | | | | | | | | | | | ^18^ | | |
|  | Declining rate | | | | | | | | | | | | | | | | | | | | | | | | | | | | | | | | | | | | | | | | | | | | | | | | | | | | | | | | | | | | | | | | | | | | | | ^45^ | | |
|  |  | | | | | | | | | | | | | | | | | | | | | | | | | | | | | | | | | | | | | | | | | | | | | | | | | | | | | | | | | | | | | | | | | | | | | |  | | |
|  | RNs / NA + LPNs | | | | | | | | | | | | | | | | | | | 0.13 (SD 0.58) | | | | | | | | | | | | | | | | | | | | | | | | | | | | | | | | | | | | | | | | | | | | | | | | | | | ^81^ | | |
| Mean % Change hours/resident/day (HPRD) (2006 to 2007) |  | | | | | | | | | | | | | | | | | | | **RN** | | | | | | | | | | | | | | | | | | | | | | **LPN** | | | | | | | | | | | | | | | | | | | | | **NA** | | | | | | | | ^30^ | | |
|  | Large Nursing Home | | | | | | | | | | | | | | | | | | | -20.263 | | | | | | | | | | | | | | | | | | | | | | 20.431 | | | | | | | | | | | | | | | | | | | | | 9.564 | | | | | | | |  | | |
|  | Medium Nursing Home | | | | | | | | | | | | | | | | | | | -18.625 | | | | | | | | | | | | | | | | | | | | | | 20.826 | | | | | | | | | | | | | | | | | | | | | 9.010 | | | | | | | |  | | |
|  | Small Nursing Home | | | | | | | | | | | | | | | | | | | 0.953 | | | | | | | | | | | | | | | | | | | | | | 5.085 | | | | | | | | | | | | | | | | | | | | | 5.108 | | | | | | | |  | | |
| Mean hrs/resident (or patient) day | NH Only | | | | | | | | | | | | | | | | | | |  | | | | | | | | | | | | | | | | | | | | | |  | | | | | | | | | | | | | | | | | | | | | 1.85(0.43) | | | | | | | | ^86^ | | |
|  |  | | | | | | | | | | | | | | | | | | |  | | | | | | | | | | | | | | | | | | | | | |  | | | | | | | | | | | | | | | | | | | | | 2.06 (0.03) | | | | | | | | ^87^ | | |
|  | All Sites | | | | | | | | | | | | | | | | | | |  | | | | | | | | | | | | | | | | | | | | | |  | | | | | | | | | | | | | | | | | | | | | 2.118 (0.425) | | | | | | | | ^85^ | | |
|  |  | | | | | | | | | | | | | | | | | | |  | | | | | | | | | | | | | | | | | | | | | |  | | | | | | | | | | | | | | | | | | | | | 2.51 | | | | | | | | ^57^ | | |
| FTE/100 Residents | NH only | | | | | | | | | | | | | | | | | | | 4.31 (3.60) | | | | | | | | | | | | | | | | | | | | | | 11.67 (6.04) | | | | | | | | | | | | | | | | | | | | | 33.54 (13.11) | | | | | | | | ^90^ | | |
|  |  | | | | | | | | | | | | | | | | | | | 21.2(6.0) | | | | | | | | | | | | | | | | | | | | | | 20.4 (4.0) | | | | | | | | | | | | | | | | | | | | | 31 (10.4) | | | | | | | | ^91^ | | |
|  | All Sites | | | | | | | | | | | | | | | | | | | 25.9 (4.6) | | | | | | | | | | | | | | | | | | | | | | 23.7(4.8) | | | | | | | | | | | | | | | | | | | | | 38.5 | | | | | | | | ^89^ | | |
|  |  | | | | | | | | | | | | | | | | | | | 8.5 (8.1) | | | | | | | | | | | | | | | | | | | | | | 11.2 (9.4) | | | | | | | | | | | | | | | | | | | | | 25.3 (8.6) | | | | | | | | ^80^ | | |
|  |  | | | | | | | | | | | | | | | | | | | n/a | | | | | | | | | | | | | | | | | | | | | | n/a | | | | | | | | | | | | | | | | | | | | | 30.5 | | | | | | | | ^88^ | | |
| Mean % Change HPRD |  | | | | | | | | | | | | | | | | | | | **1997-2007 (%)** | | | | | | | | | | | | | | | | | | | | | | | | | | | | | | | | | | | | | | | | | | | | | | | | | | |  | | |
|  | Large Nursing Home | | | | | | | | | | | | | | | | | | | **Profit** | | | | | | | | | | | | | | | | | | | | | | **Non-profit** | | | | | | | | | | | | | | | | | | | | | **Government** | | | | | | | |  | | |
|  | RN | | | | | | | | | | | | | | | | | | | -26.096 | | | | | | | | | | | | | | | | | | | | | | -14.163 | | | | | | | | | | | | | | | | | | | | | -3.967 | | | | | | | | ^30^ | | |
|  | LPN | | | | | | | | | | | | | | | | | | | 22.074 | | | | | | | | | | | | | | | | | | | | | | 17.159 | | | | | | | | | | | | | | | | | | | | | 19.400 | | | | | | | |  | | |
|  | NA | | | | | | | | | | | | | | | | | | | 10.678 | | | | | | | | | | | | | | | | | | | | | | 5.709 | | | | | | | | | | | | | | | | | | | | | 15.150 | | | | | | | |  | | |
|  | Medium Nursing Home | | | | | | | | | | | | | | | | | | |  | | | | | | | | | | | | | | | | | | | | | |  | | | | | | | | | | | | | | | | | | | | |  | | | | | | | |  | | |
|  | RN | | | | | | | | | | | | | | | | | | | -24.680 | | | | | | | | | | | | | | | | | | | | | | -8.938 | | | | | | | | | | | | | | | | | | | | | 11.326 | | | | | | | |  | | |
|  | LPN | | | | | | | | | | | | | | | | | | | 21.568 | | | | | | | | | | | | | | | | | | | | | | 18.799 | | | | | | | | | | | | | | | | | | | | | 19.656 | | | | | | | |  | | |
|  | NA | | | | | | | | | | | | | | | | | | | 9.526 | | | | | | | | | | | | | | | | | | | | | | 7.354 | | | | | | | | | | | | | | | | | | | | | 10.420 | | | | | | | |  | | |
|  | Small Nursing Home | | | | | | | | | | | | | | | | | | |  | | | | | | | | | | | | | | | | | | | | | |  | | | | | | | | | | | | | | | | | | | | |  | | | | | | | |  | | |
|  | RN | | | | | | | | | | | | | | | | | | | -16.568 | | | | | | | | | | | | | | | | | | | | | | 5.140 | | | | | | | | | | | | | | | | | | | | | 19.271 | | | | | | | |  | | |
|  | LPN | | | | | | | | | | | | | | | | | | | 9.324 | | | | | | | | | | | | | | | | | | | | | | 3.506 | | | | | | | | | | | | | | | | | | | | | 0.643 | | | | | | | |  | | |
|  | NA | | | | | | | | | | | | | | | | | | | 4.883 | | | | | | | | | | | | | | | | | | | | | | 5.053 | | | | | | | | | | | | | | | | | | | | | 5.814 | | | | | | | |  | | |
| Mean HPRD |  | | | | | | | | | | | | | | | | | | | **Profit** | | | | | | | | | | | | | | | | | | | | | | **Non-profit** | | | | | | | | | | | | | | | | | | | | |  | | | | | | | |  | | |
|  |  | | | | | | | | | | | | | | | | | | | 2.071** | | | | | | | | | | | | | | | | | | | | | | 2.346** | | | | | | | | | | | | | | | | | | | | |  | | | | | | | | ^87^ | | |
| **DEMAND** | | | | | | | | | | | | | | | | | | | | | | | | | | | | | | | | | | | | | | | | | | | | | | | | | | | | | | | | | | | | | | | | | | | | | | | | | |
| **Themes** | **Outcomes** | | | | | | | | | | | | | | | | | | |  | | | | | | | | | | | | | | | | | | | | | | | | | | | | | | | | | | | | | | | | | | | | | | | | | | | **Ref.** | | |
| **In Profession** | **Job** | | | | | | | | | | | | | **2000** | | | | | | | | | | | | | | | | | | | **2010 Projected** | | | | | | | | | | | | | | | | | | | | | | | | | | | | **% Increase** | | | | | | | | | |  | | |
| **Projected Growth**  Number of projected jobs and % increase | NAs, Orderlies, and Attendants | | | | | | | | | | | | | 1,373,000 | | | | | | | | | | | | | | | | | | | 1,697,000 | | | | | | | | | | | | | | | | | | | | | | | | | | | | 23.5 | | | | | | | | | | ^84^ | | |
|  | HHAs | | | | | | | | | | | | | 615,000 | | | | | | | | | | | | | | | | | | | 907,000 | | | | | | | | | | | | | | | | | | | | | | | | | | | | 47.3 | | | | | | | | | |  | | |
|  |  | | | | | | | | | | | | | Ranked 8^th^ fastest growing professions | | | | | | | | | | | | | | | | | | |  | | | | | | | | | | | | | | | | | | | | | | | | | | | |  | | | | | | | | | |  | | |
|  | Personal & HCAs | | | | | | | | | | | | | 414,000 | | | | | | | | | | | | | | | | | | | 672,000 | | | | | | | | | | | | | | | | | | | | | | | | | | | | 62.5 | | | | | | | | | |  | | |
|  | Total | | | | | | | | | | | | | 2,402,000 | | | | | | | | | | | | | | | | | | | 3,276,000 | | | | | | | | | | | | | | | | | | | | | | | | | | | | 36.3 | | | | | | | | | |  | | |
|  | Net Change Over Decade | | | | | | | | | | | | | | | | | | | | | | | | | | | | | | | | +874,000 | | | | | | | | | | | | | | | | | | | | | | | | | | | |  | | | | | | | | | |  | | |
|  | Growth in employment for direct care workers is more than double that of projected growth in overall employment (15.2%) during this same period | | | | | | | | | | | | | | | | | | | | | | | | | | | | | | | | | | | | | | | | | | | | | | | | | | | | | | | | | | | | | | | | | | | | | |  | | |
|  |  | | | | | | | | | | | | | **Total HCA demand/projected** | | | | | | | | | | | | | | | | | | | | | | | | | | | | | | | | | | | | | | | | | | | | | | | | | | | | | | | | |  | | |
|  |  | | | | | | | | | | | | | **2010** | | | | | | | | | | | | | | | | | | | **2020 Projected** | | | | | | | | | | | | | | | | | | | | | | | | | | | | **% Increase** | | | | | | | | | |  | | |
|  | Healthcare support occupations | | | | | | | | | | | | | Projected to be the fastest growing occupational group | | | | | | | | | | | | | | | | | | | | | | | | | | | | | | | | | | | | | | | | | | | | | | | | | | | | | | | | | ^74^ | | |
|  | HCA | | | | | | | | | | | | | 1526 (Alberta) | | | | | | | | | | | | | | | | | | | 3264 (Alberta) | | | | | | | | | | | | | | | | | | | | | | | | | | | | 114 | | | | | | | | | | ^48^ | | |
|  | HHA | | | | | | | | | | | | |  | | | | | | | | | | | | | | | | | | | +706,300 | | | | | | | | | | | | | | | | | | | | | | | | | | | | 69.4 | | | | | | | | | | ^74^ | | |
|  |  | | | | | | | | | | | | | Home health aide is projected to be the 3^rd^ fastest growing occupation in the US between 2008-2018 | | | | | | | | | | | | | | | | | | | | | | | | | | | | | | | | | | | | | | | | | | | | | | | | | | | | | | | | | ^72^ | | |
| **Tenure** |  | | | | | | | | | | | | | **Multiple Settings** | | | | | | | | | | | | | | | | | | | **NH** | | | | | | | | | | | | | | | | | | | | | | | | | | | | **HH** | | | | | | | | | |  | | |
| Mean months in Profession |  | | | | | | | | | | | | | 148.8 (134.4) | | | | | | | | | | | | | | | | | | |  | | | | | | | | | | | | | | | | | | | | | | | | | | | |  | | | | | | | | | | ^80^ | | |
|  |  | | | | | | | | | | | | | 130.8 (85.2) for NAs w/ >1 yr NA work during last 5 yrs | | | | | | | | | | | | | | | | | | | | | | | | | | | | | | | | | | | | | | | | | | | | | | | | | | | | | | | | | ^66^ | | |
|  |  | | | | | | | | | | | | |  | | | | | | | | | | | | | | | | | | | 79.2 | | | | | | | | | | | | | | | | | | | | | | | | | | | |  | | | | | | | | | | ^68^ | | |
| Mean % by years | < 1 | | | | | | | | | | | | | 3.6 (stayers) 64.1 (leavers) | | | | | | | | | | | | | | | | | | |  | | | | | | | | | | | | | | | | | | | | | | | | | | | |  | | | | | | | | | | ^32^ | | |
|  |  | | | | | | | | | | | | | 11.1 | | | | | | | | | | | | | | | | | | |  | | | | | | | | | | | | | | | | | | | | | | | | | | | |  | | | | | | | | | | ^54^ | | |
|  |  | | | | | | | | | | | | | 11.4 | | | | | | | | | | | | | | | | | | |  | | | | | | | | | | | | | | | | | | | | | | | | | | | |  | | | | | | | | | | ^24^ | | |
|  |  | | | | | | | | | | | | | 14 | | | | | | | | | | | | | | | | | | |  | | | | | | | | | | | | | | | | | | | | | | | | | | | |  | | | | | | | | | | ^50^ | | |
|  |  | | | | | | | | | | | | | 21.1 (permanent FT) | | | | | | | | | | | | | | | | | | |  | | | | | | | | | | | | | | | | | | | | | | | | | | | |  | | | | | | | | | | ^67^ | | |
|  |  | | | | | | | | | | | | | 22.9 (temp/casual FT) | | | | | | | | | | | | | | | | | | |  | | | | | | | | | | | | | | | | | | | | | | | | | | | |  | | | | | | | | | |  | | |
|  |  | | | | | | | | | | | | | 26.6 (temp/casual PT) | | | | | | | | | | | | | | | | | | |  | | | | | | | | | | | | | | | | | | | | | | | | | | | |  | | | | | | | | | |  | | |
|  | <1 to <2 | | | | | | | | | | | | |  | | | | | | | | | | | | | | | | | | | 8.4 | | | | | | | | | | | | | | | | | | | | | | | | | | | |  | | | | | | | | | | ^24^ | | |
|  |  | | | | | | | | | | | | |  | | | | | | | | | | | | | | | | | | | 10 | | | | | | | | | | | | | | | | | | | | | | | | | | | |  | | | | | | | | | | ^50^ | | |
|  | <2 | | | | | | | | | | | | | 8.3 | | | | | | | | | | | | | | | | | | |  | | | | | | | | | | | | | | | | | | | | | | | | | | | |  | | | | | | | | | | ^62^ | | |
|  |  | | | | | | | | | | | | | 19.3 | | | | | | | | | | | | | | | | | | |  | | | | | | | | | | | | | | | | | | | | | | | | | | | | 9.0 (1.4)** | | | | | | | | | | ^35^ | | |
|  |  | | | | | | | | | | | | |  | | | | | | | | | | | | | | | | | | | 19.3 | | | | | | | | | | | | | | | | | | | | | | | | | | | |  | | | | | | | | | | ^53^ | | |
|  | >1-5 | | | | | | | | | | | | | 26.3 | | | | | | | | | | | | | | | | | | |  | | | | | | | | | | | | | | | | | | | | | | | | | | | | 21.5 (2.1)* | | | | | | | | | | ^35^ | | |
|  |  | | | | | | | | | | | | | 29.0 | | | | | | | | | | | | | | | | | | |  | | | | | | | | | | | | | | | | | | | | | | | | | | | |  | | | | | | | | | | ^62^ | | |
|  |  | | | | | | | | | | | | |  | | | | | | | | | | | | | | | | | | | 26.2 | | | | | | | | | | | | | | | | | | | | | | | | | | | |  | | | | | | | | | | ^24^ | | |
|  |  | | | | | | | | | | | | |  | | | | | | | | | | | | | | | | | | |  | | | | | | | | | | | | | | | | | | | | | | | | | | | |  | | | | | | | | | | ^53^ | | |
|  |  | | | | | | | | | | | | |  | | | | | | | | | | | | | | | | | | | 27 | | | | | | | | | | | | | | | | | | | | | | | | | | | |  | | | | | | | | | | ^50^ | | |
|  |  | | | | | | | | | | | | |  | | | | | | | | | | | | | | | | | | | 34.4 | | | | | | | | | | | | | | | | | | | | | | | | | | | |  | | | | | | | | | | ^54^ | | |
|  |  | | | | | | | | | | | | |  | | | | | | | | | | | | | | | | | | | 40.3 (permanent FT) | | | | | | | | | | | | | | | | | | | | | | | | | | | |  | | | | | | | | | | ^67^ | | |
|  |  | | | | | | | | | | | | |  | | | | | | | | | | | | | | | | | | | 49.3 (temp/casual FT) | | | | | | | | | | | | | | | | | | | | | | | | | | | |  | | | | | | | | | |  | | |
|  |  | | | | | | | | | | | | |  | | | | | | | | | | | | | | | | | | | 52.2 (temp/casual PT) | | | | | | | | | | | | | | | | | | | | | | | | | | | |  | | | | | | | | | |  | | |
|  |  | | | | | | | | | | | | |  | | | | | | | | | | | | | | | | | | | 58.3 (stayers) 23.4 (leavers) | | | | | | | | | | | | | | | | | | | | | | | | | | | |  | | | | | | | | | | ^32^ | | |
|  | >5 to <10 | | | | | | | | | | | | | 19.8 | | | | | | | | | | | | | | | | | | |  | | | | | | | | | | | | | | | | | | | | | | | | | | | | 19.7(2.4) | | | | | | | | | | ^35^ | | |
|  |  | | | | | | | | | | | | | 22.6 | | | | | | | | | | | | | | | | | | |  | | | | | | | | | | | | | | | | | | | | | | | | | | | |  | | | | | | | | | | ^62^ | | |
|  |  | | | | | | | | | | | | |  | | | | | | | | | | | | | | | | | | | 5.6 (stayers) 8.3 (leavers) | | | | | | | | | | | | | | | | | | | | | | | | | | | |  | | | | | | | | | | ^32^ | | |
|  |  | | | | | | | | | | | | |  | | | | | | | | | | | | | | | | | | | 15.5 (non-citizen) | | | | | | | | | | | | | | | | | | | | | | | | | | | |  | | | | | | | | | | ^54^ | | |
|  |  | | | | | | | | | | | | |  | | | | | | | | | | | | | | | | | | | 19.2 | | | | | | | | | | | | | | | | | | | | | | | | | | | |  | | | | | | | | | | ^24^ | | |
|  |  | | | | | | | | | | | | |  | | | | | | | | | | | | | | | | | | | 19.2 | | | | | | | | | | | | | | | | | | | | | | | | | | | |  | | | | | | | | | | ^53^ | | |
|  |  | | | | | | | | | | | | |  | | | | | | | | | | | | | | | | | | | 19.2 | | | | | | | | | | | | | | | | | | | | | | | | | | | |  | | | | | | | | | | ^54^ | | |
|  |  | | | | | | | | | | | | |  | | | | | | | | | | | | | | | | | | | 19.3 (US born) | | | | | | | | | | | | | | | | | | | | | | | | | | | |  | | | | | | | | | |  | | |
|  |  | | | | | | | | | | | | |  | | | | | | | | | | | | | | | | | | | 20.8 (naturalized citizen) | | | | | | | | | | | | | | | | | | | | | | | | | | | |  | | | | | | | | | |  | | |
|  | <6 | | | | | | | | | | | | |  | | | | | | | | | | | | | | | | | | | 50 | | | | | | | | | | | | | | | | | | | | | | | | | | | |  | | | | | | | | | | ^50^ | | |
|  | <7 | | | | | | | | | | | | |  | | | | | | | | | | | | | | | | | | | 8.5 (temp/casual PT) | | | | | | | | | | | | | | | | | | | | | | | | | | | |  | | | | | | | | | | ^67^ | | |
|  |  | | | | | | | | | | | | |  | | | | | | | | | | | | | | | | | | | 11.8 (temp/casual FT) | | | | | | | | | | | | | | | | | | | | | | | | | | | |  | | | | | | | | | |  | | |
|  |  | | | | | | | | | | | | |  | | | | | | | | | | | | | | | | | | | 23.2 (permanent FT) | | | | | | | | | | | | | | | | | | | | | | | | | | | |  | | | | | | | | | |  | | |
|  | >10-15 | | | | | | | | | | | | |  | | | | | | | | | | | | | | | | | | | 6.5 (stayers) 0.7 (leavers) | | | | | | | | | | | | | | | | | | | | | | | | | | | |  | | | | | | | | | | ^32^ | | |
|  | >11 | | | | | | | | | | | | | 39.9 | | | | | | | | | | | | | | | | | | |  | | | | | | | | | | | | | | | | | | | | | | | | | | | |  | | | | | | | | | | ^62^ | | |
|  |  | | | | | | | | | | | | |  | | | | | | | | | | | | | | | | | | |  | | | | | | | | | | | | | | | | | | | | | | | | | | | |  | | | | | | | | | |  | | |
|  |  | | | | | | | | | | | | |  | | | | | | | | | | | | | | | | | | | 16.0 (non-citizen) | | | | | | | | | | | | | | | | | | | | | | | | | | | |  | | | | | | | | | | ^54^ | | |
|  |  | | | | | | | | | | | | |  | | | | | | | | | | | | | | | | | | | 36.6 (US-born) | | | | | | | | | | | | | | | | | | | | | | | | | | | |  | | | | | | | | | |  | | |
|  |  | | | | | | | | | | | | |  | | | | | | | | | | | | | | | | | | | 42.8 (naturalized citizen) | | | | | | | | | | | | | | | | | | | | | | | | | | | |  | | | | | | | | | |  | | |
|  |  | | | | | | | | | | | | |  | | | | | | | | | | | | | | | | | | |  | | | | | | | | | | | | | | | | | | | | | | | | | | | | 49.8** | | | | | | | | | | ^35^ | | |
|  | 11 to 20 | | | | | | | | | | | | | 22.4 | | | | | | | | | | | | | | | | | | |  | | | | | | | | | | | | | | | | | | | | | | | | | | | |  | | | | | | | | | | ^35^ | | |
|  |  | | | | | | | | | | | | |  | | | | | | | | | | | | | | | | | | | 22.3 | | | | | | | | | | | | | | | | | | | | | | | | | | | |  | | | | | | | | | | ^24^ | | |
|  |  | | | | | | | | | | | | |  | | | | | | | | | | | | | | | | | | | 22.8 | | | | | | | | | | | | | | | | | | | | | | | | | | | |  | | | | | | | | | | ^53^ | | |
|  | >15 | | | | | | | | | | | | |  | | | | | | | | | | | | | | | | | | | 6 (stayers) 3.4(leavers) | | | | | | | | | | | | | | | | | | | | | | | | | | | |  | | | | | | | | | | ^32^ | | |
|  | >20 | | | | | | | | | | | | | 12.3 | | | | | | | | | | | | | | | | | | |  | | | | | | | | | | | | | | | | | | | | | | | | | | | |  | | | | | | | | | | ^35^ | | |
|  |  | | | | | | | | | | | | |  | | | | | | | | | | | | | | | | | | | 12.4 | | | | | | | | | | | | | | | | | | | | | | | | | | | |  | | | | | | | | | | ^24^ | | |
|  |  | | | | | | | | | | | | |  | | | | | | | | | | | | | | | | | | | 12.5 | | | | | | | | | | | | | | | | | | | | | | | | | | | |  | | | | | | | | | | ^53^ | | |
| **Turnover**  Mean % |  | | | | | | | | | | | | |  | | | | | | | | | | | | | | | | | | | **Stayers** | | | | | | | | | | | | | | | | | | | | | | | | | | | | **Leavers** | | | | | | | | | |  | | |
|  | Turnover | | | | | | | | | | | | |  | | | | | | | | | | | | | | | | | | | 53.75 | | | | | | | | | | | | | | | | | | | | | | | | | | | | 46.3 | | | | | | | | | | ^32^ | | |
|  | Unemployed and looking | | | | | | | | | | | | | | | | | | | | | | | | | | | | | | | |  | | | | | | | | | | | | | | | | | | | | | | | | | | | | 66.2 | | | | | | | | | |  | | |
|  | Personal caregiver in acute care | | | | | | | | | | | | | | | | | | | | | | | | | | | | | | | |  | | | | | | | | | | | | | | | | | | | | | | | | | | | | 15.2 | | | | | | | | | |  | | |
|  | Switched to a non-medical job | | | | | | | | | | | | | | | | | | | | | | | | | | | | | | | |  | | | | | | | | | | | | | | | | | | | | | | | | | | | | 18.6 | | | | | | | | | |  | | |
|  | Turnover | | | | | | | | | | | | |  | | | | | | | | | | | | | | | | | | |  | | | | | | | | | | | | | | | | | | | | | | | | | | | | 37 | | | | | | | | | | ^65^ | | |
|  | Health or welfare sector | | | | | | | | | | | | | | | | | | | | | | | | | | | | | | | |  | | | | | | | | | | | | | | | | | | | | | | | | | | | | 13.4 | | | | | | | | | |  | | |
|  | Other sector unrelated to health | | | | | | | | | | | | | | | | | | | | | | | | | | | | | | | |  | | | | | | | | | | | | | | | | | | | | | | | | | | | | 6.7 | | | | | | | | | |  | | |
|  | Undertaking education | | | | | | | | | | | | | | | | | | | | | | | | | | | | | | | |  | | | | | | | | | | | | | | | | | | | | | | | | | | | | 12.1 | | | | | | | | | |  | | |
|  | Outside labor market | | | | | | | | | | | | | | | | | | | | | | | | | | | | | | | |  | | | | | | | | | | | | | | | | | | | | | | | | | | | | 4.8 | | | | | | | | | |  | | |
| **Issues associated with reporting turnover** | - No comprehensive definition for turnover - Most administrators included voluntary and involuntary turnover, all work shifts, and PT workers in the reported turnover rates. ~30% did not include at least one of these factors, when omissions were corrected the recalculated rates were generally higher - Most did not include turnover of agency staff | | | | | | | | | | | | | | | | | | | | | | | | | | | | | | | | | | | | | | | | | | | | | | | | | | | | | | | | | | | | | | | | | | | | | | ^31^ | | |
| **Intent to leave** | Do not expect next job to be NA | | | | | | | | | | | | | | | | | | | | | | | | | | | | | | | | 48 % (more Non-Whites intend to leave  the profession) | | | | | | | | | | | | | | | | | | | | | | | | | | | | | | | | | | | | | | ^55^ | | |
|  |  | | | | | | | | | | | | | | | | | | | | | | | | | | | | | | | | 2.22 (SD=1.06, 1= not at all, 4= extremely) | | | | | | | | | | | | | | | | | | | | | | | | | | | | | | | | | | | | | | ^68^ | | |
| **Transition from Direct care**  Mean % | Not working | | | | | | | | | | | | | | | | | | | | | | | | | | | | | | | | | 31.5 | | | | | | | | | | | | | | | | | | | | | | | | | | | | | | | | | | | | | ^72^ | | |
|  | Not working – not disabled | | | | | | | | | | | | | | | | | | | | | | | | | | | | | | | | | 23.8 | | | | | | | | | | | | | | | | | | | | | | | | | | | | | | | | | | | | |  | | |
|  | Not working – disabled | | | | | | | | | | | | | | | | | | | | | | | | | | | | | | | | | 5.5 working - disabledeen the US with in a percentage form as welllity conditions in the client' | | | | | | | | | | | | | | | | | | | | | | | | | | | | | | | | | | | | |  | | |
|  | Not working – in school | | | | | | | | | | | | | | | | | | | | | | | | | | | | | | | | | 2.2 working - disabledeen the US with in a percentage form as welllity conditions in the client' | | | | | | | | | | | | | | | | | | | | | | | | | | | | | | | | | | | | |  | | |
|  | New direct care job | | | | | | | | | | | | | | | | | | | | | | | | | | | | | | | | | 29.3 | | | | | | | | | | | | | | | | | | | | | | | | | | | | | | | | | | | | |  | | |
|  | RN | | | | | | | | | | | | | | | | | | | | | | | | | | | | | | | | | 3.5 | | | | | | | | | | | | | | | | | | | | | | | | | | | | | | | | | | | | |  | | |
|  | LPNost popular being cleaning/household and retail services | | | | | | | | | | | | | | | | | | | | | | | | | | | | | | | | | 1.4 | | | | | | | | | | | | | | | | | | | | | | | | | | | | | | | | | | | | |  | | |
|  | Medical Technician | | | | | | | | | | | | | | | | | | | | | | | | | | | | | | | | | 1.9 | | | | | | | | | | | | | | | | | | | | | | | | | | | | | | | | | | | | |  | | |
|  | Child care | | | | | | | | | | | | | | | | | | | | | | | | | | | | | | | | | 1.6 | | | | | | | | | | | | | | | | | | | | | | | | | | | | | | | | | | | | |  | | |
|  | Non-child care and non-health sector jobs (most popular being cleaning/household) | | | | | | | | | | | | | | | | | | | | | | | | | | | | | | | | | 29 | | | | | | | | | | | | | | | | | | | | | | | | | | | | | | | | | | | | |  | | |
|  | Multiple level processes exist in the UK (National Vocational Qualification (NVQ) and Japan (Home Helper) allow HCAs and home workers to advance in higher levels of care for the patient | | | | | | | | | | | | | | | | | | | | | | | | | | | | | | | | | | | | | | | | | | | | | | | | | | | | | | | | | | | | | | | | | | | | | | ^47,69^ | | |
|  | Of transitions, move to higher-paying occupations | | | | | | | | | | | | | | | | | | | | | | | | | | | | | | | | | 56  36 (RNs excluded) | | | | | | | | | | | | | | | | | | | | | | | | | | | | | | | | | | | | | ^77^ | | |
|  | - On average, workers earned significantly higher wages after transition jobs in other healthcare occupations, office administration, or professional positions (p<.05) - Wages declined in personal care/sales/food services occupation | | | | | | | | | | | | | | | | | | | | | | | | | | | | | | | | | | | | | | | | | | | | | | | | | | | | | | | | | | | | | | | | | | | | | |  | | |
| **In Facilities** |  | | | | | | | | | | | | | **Multiple Settings** | | | | | | | | | | | | | | | | | | | **NH** | | | | | | | | | | | | | | | | | | | | | | | | | | | | **HH** | | | | | | | | | |  | | |
| **Tenure** |  | | | | | | | | | | | | | 61.4 | | | | | | | | | | | | | | | | | | |  | | | | | | | | | | | | | | | | | | | | | | | | | | | |  | | | | | | | | | | ^35^ | | |
| Mean months in facility |  | | | | | | | | | | | | | 56.0 | | | | | | | | | | | | | | | | | | |  | | | | | | | | | | | | | | | | | | | | | | | | | | | |  | | | | | | | | | | ^34^ | | |
|  |  | | | | | | | | | | | | | 46.8 | | | | | | | | | | | | | | | | | | |  | | | | | | | | | | | | | | | | | | | | | | | | | | | |  | | | | | | | | | | ^80^ | | |
|  |  | | | | | | | | | | | | | 27.19 | | | | | | | | | | | | | | | | | | |  | | | | | | | | | | | | | | | | | | | | | | | | | | | |  | | | | | | | | | | ^77^ | | |
|  |  | | | | | | | | | | | | |  | | | | | | | | | | | | | | | | | | | 70.8 | | | | | | | | | | | | | | | | | | | | | | | | | | | |  | | | | | | | | | | ^68^ | | |
|  |  | | | | | | | | | | | | |  | | | | | | | | | | | | | | | | | | | 58.6 | | | | | | | | | | | | | | | | | | | | | | | | | | | |  | | | | | | | | | | ^24^ | | |
|  |  | | | | | | | | | | | | |  | | | | | | | | | | | | | | | | | | | 25.96 | | | | | | | | | | | | | | | | | | | | | | | | | | | |  | | | | | | | | | | ^56^ | | |
|  |  | | | | | | | | | | | | | 109.5 | | | | | | | | | | | | | | | | | | | 118.3 | | | | | | | | | | | | | | | | | | | | | | | | | | | | 104.6 | | | | | | | | | | ^79^ | | |
|  |  | | | | | | | | | | | | |  | | | | | | | | | | | | | | | | | | |  | | | | | | | | | | | | | | | | | | | | | | | | | | | | 57.6 | | | | | | | | | | ^73^ | | |
|  |  | | | | | | | | | | | | |  | | | | | | | | | | | | | | | | | | | **Assisted living** | | | | | | | | | | | | | | | | | | | | | | | | | | | | **Adult Day Services** | | | | | | | | | |  | | |
|  |  | | | | | | | | | | | | |  | | | | | | | | | | | | | | | | | | | 98.0 | | | | | | | | | | | | | | | | | | | | | | | | | | | | 110.3 | | | | | | | | | | ^79^ | | |
| Mean % by years |  | | | | | | | | | | | | |  | | | | | | | | | | | | | | | | | | | **NH** | | | | | | | | | | | | | | | | | | | | | | | | | | | |  | | | | | | | | | |  | | |
|  | <1 | | | | | | | | | | | | |  | | | | | | | | | | | | | | | | | | | 28.7 | | | | | | | | | | | | | | | | | | | | | | | | | | | |  | | | | | | | | | | ^24^ | | |
|  |  | | | | | | | | | | | | |  | | | | | | | | | | | | | | | | | | | 40 | | | | | | | | | | | | | | | | | | | | | | | | | | | |  | | | | | | | | | | ^50^ | | |
|  | >1 | | | | | | | | | | | | |  | | | | | | | | | | | | | | | | | | | 61 | | | | | | | | | | | | | | | | | | | | | | | | | | | |  | | | | | | | | | | ^50^ | | |
|  |  | | | | | | | | | | | | |  | | | | | | | | | | | | | | | | | | | 54.2(1.0) | | | | | | | | | | | | | | | | | | | | | | | | | | | |  | | | | | | | | | | ^56^ | | |
|  | <1 to <2 | | | | | | | | | | | | |  | | | | | | | | | | | | | | | | | | | 13.8 | | | | | | | | | | | | | | | | | | | | | | | | | | | |  | | | | | | | | | | ^24^ | | |
|  | <2 | | | | | | | | | | | | |  | | | | | | | | | | | | | | | | | | | 41.8 (rural) | | | | | | | | | | | | | | | | | | | | | | | | | | | |  | | | | | | | | | | ^53^ | | |
|  |  | | | | | | | | | | | | |  | | | | | | | | | | | | | | | | | | | 42.3 (urban) | | | | | | | | | | | | | | | | | | | | | | | | | | | |  | | | | | | | | | |  | | |
|  |  | | | | | | | | | | | | |  | | | | | | | | | | | | | | | | | | | 42.6 (micropolitan) | | | | | | | | | | | | | | | | | | | | | | | | | | | |  | | | | | | | | | |  | | |
|  | >1 to < 5 | | | | | | | | | | | | |  | | | | | | | | | | | | | | | | | | | 27.4 (urban) | | | | | | | | | | | | | | | | | | | | | | | | | | | |  | | | | | | | | | | ^53^ | | |
|  |  | | | | | | | | | | | | |  | | | | | | | | | | | | | | | | | | | 26.2 (micropolitan) | | | | | | | | | | | | | | | | | | | | | | | | | | | |  | | | | | | | | | |  | | |
|  |  | | | | | | | | | | | | |  | | | | | | | | | | | | | | | | | | | 25.8 | | | | | | | | | | | | | | | | | | | | | | | | | | | |  | | | | | | | | | | ^24^ | | |
|  |  | | | | | | | | | | | | |  | | | | | | | | | | | | | | | | | | | 25.6 (rural) | | | | | | | | | | | | | | | | | | | | | | | | | | | |  | | | | | | | | | | ^53^ | | |
|  | >5 to <10 | | | | | | | | | | | | |  | | | | | | | | | | | | | | | | | | | 14.0 (micropolitan) | | | | | | | | | | | | | | | | | | | | | | | | | | | |  | | | | | | | | | | ^53^ | | |
|  |  | | | | | | | | | | | | |  | | | | | | | | | | | | | | | | | | | 13.5 (urban) | | | | | | | | | | | | | | | | | | | | | | | | | | | |  | | | | | | | | | |  | | |
|  |  | | | | | | | | | | | | |  | | | | | | | | | | | | | | | | | | | 13.1 | | | | | | | | | | | | | | | | | | | | | | | | | | | |  | | | | | | | | | | ^24^ | | |
|  |  | | | | | | | | | | | | |  | | | | | | | | | | | | | | | | | | | 12.9 (rural) | | | | | | | | | | | | | | | | | | | | | | | | | | | |  | | | | | | | | | | ^53^ | | |
|  | >10 | | | | | | | | | | | | |  | | | | | | | | | | | | | | | | | | | 16.4  17.1 | | | | | | | | | | | | | | | | | | | | | | | | | | | |  | | | | | | | | | | ^24^  ^53^ | | |
|  | 11 to 20 | | | | | | | | | | | | |  | | | | | | | | | | | | | | | | | | | 14.9 (rural) | | | | | | | | | | | | | | | | | | | | | | | | | | | |  | | | | | | | | | | ^53^ | | |
|  |  | | | | | | | | | | | | |  | | | | | | | | | | | | | | | | | | | 12.5(urban) | | | | | | | | | | | | | | | | | | | | | | | | | | | |  | | | | | | | | | |  | | |
|  |  | | | | | | | | | | | | |  | | | | | | | | | | | | | | | | | | | 10.3(micropolitan) | | | | | | | | | | | | | | | | | | | | | | | | | | | |  | | | | | | | | | |  | | |
| Odds ratio | Direct care occupation  (ratio >1.0 =more likely to stay) | | | | | | | | | | | | | **H**  2.4 | | | | | | | | | | | | | | | | | | | **NH**  1.7 | | | | | | | | | | | | | | | | | | | | | | | | | | | | **HH**  1.0 | | | | | | | | | | ^22^ | | |
| **Effects on Tenure** | **Community** | | | | | | | | | | | | | | | | | | | | | | | | | | | | | | | | | | | | | | | | | | | | | | | | |  | | | | | |  | | | | | | | | | | | | | | |  | | |
|  | Higher county unemployment rate | | | | | | | | | | | | | | | | | | | | | | | | | | | | | | | | | | | | | | | | | | | | | | | | | + | | | | | |  | | | | | | | | | | | | | | | ^56^ | | |
|  | # of home health agencies for older people | | | | | | | | | | | | | | | | | | | | | | | | | | | | | | | | | | | | | | | | | | | | | | | | | + | | | | | | (NH-employed HCAs) | | | | | | | | | | | | | | |  | | |
|  | **General Facility Characteristics** | | | | | | | | | | | | | | | | | | | | | | | | | | | | | | | | | | | | | | | | | | | | | | | | |  | | | | | |  | | | | | | | | | | | | | | |  | | |
|  | Higher wages | | | | | | | | | | | | | | | | | | | | | | | | | | | | | | | | | | | | | | | | | | | | | | | | | + | | | | | |  | | | | | | | | | | | | | | |  | | |
|  | Paid time off (holidays, personal/vacation) | | | | | | | | | | | | | | | | | | | | | | | | | | | | | | | | | | | | | | | | | | | | | | | | | + | | | | | |  | | | | | | | | | | | | | | |  | | |
|  | Availability of pension | | | | | | | | | | | | | | | | | | | | | | | | | | | | | | | | | | | | | | | | | | | | | | | | | + | | | | | |  | | | | | | | | | | | | | | |  | | |
|  | Working in a facility that valued the work of CNAs | | | | | | | | | | | | | | | | | | | | | | | | | | | | | | | | | | | | | | | | | | | | | | | | | — | | | | | |  | | | | | | | | | | | | | | |  | | |
|  | For-profit | | | | | | | | | | | | | | | | | | | | | | | | | | | | | | | | | | | | | | | | | | | | | | | | | NS | | | | | |  | | | | | | | | | | | | | | |  | | |
|  | Health insurance coverage | | | | | | | | | | | | | | | | | | | | | | | | | | | | | | | | | | | | | | | | | | | | | | | | | NS | | | | | |  | | | | | | | | | | | | | | |  | | |
|  | Organizational culture | | | | | | | | | | | | | | | | | | | | | | | | | | | | | | | | | | | | | | | | | | | | | | | | | NS | | | | | |  | | | | | | | | | | | | | | |  | | |
|  | **Staffing** | | | | | | | | | | | | | | | | | | | | | | | | | | | | | | | | | | | | | | | | | | | | | | | | |  | | | | | |  | | | | | | | | | | | | | | |  | | |
|  | Assigned a mentor in first job | | | | | | | | | | | | | | | | | | | | | | | | | | | | | | | | | | | | | | | | | | | | | | | | | + | | | | | |  | | | | | | | | | | | | | | |  | | |
|  | Special units in facility | | | | | | | | | | | | | | | | | | | | | | | | | | | | | | | | | | | | | | | | | | | | | | | | | + | | | | | |  | | | | | | | | | | | | | | |  | | |
|  | CNA HPPD | | | | | | | | | | | | | | | | | | | | | | | | | | | | | | | | | | | | | | | | | | | | | | | | | NS | | | | | |  | | | | | | | | | | | | | | |  | | |
|  | RN HPPD | | | | | | | | | | | | | | | | | | | | | | | | | | | | | | | | | | | | | | | | | | | | | | | | | NS | | | | | |  | | | | | | | | | | | | | | |  | | |
| **Turnover** | **Annual** | | | | | | | | | | | | | **6 months** | | | | | | | | | | | | | | | | | | | **3 months** | | | | | | | | | | | | | | | | | | | | | | | | | | | |  | | | | | | | | | |  | | |
| Mean % of Turnover rates |  | | | | | | | | | | | | | 32.6 | | | | | | | | | | | | | | | | | | |  | | | | | | | | | | | | | | | | | | | | | | | | | | | | Multiple Settings | | | | | | | | | | ^93^ | | |
|  | 170.5 | | | | | | | | | | | | |  | | | | | | | | | | | | | | | | | | |  | | | | | | | | | | | | | | | | | | | | | | | | | | | | NH | | | | | | | | | | ^86^ | | |
|  | 119 | | | | | | | | | | | | |  | | | | | | | | | | | | | | | | | | |  | | | | | | | | | | | | | | | | | | | | | | | | | | | |  | | | | | | | | | | ^31^ | | |
|  | 107 | | | | | | | | | | | | |  | | | | | | | | | | | | | | | | | | |  | | | | | | | | | | | | | | | | | | | | | | | | | | | |  | | | | | | | | | | ^83^ | | |
|  | 59.4 | | | | | | | | | | | | |  | | | | | | | | | | | | | | | | | | |  | | | | | | | | | | | | | | | | | | | | | | | | | | | |  | | | | | | | | | | ^91^ | | |
|  | 98.6 | | | | | | | | | | | | |  | | | | | | | | | | | | | | | | | | |  | | | | | | | | | | | | | | | | | | | | | | | | | | | |  | | | | | | | | | | ^89^ | | |
|  | 77 | | | | | | | | | | | | |  | | | | | | | | | | | | | | | | | | |  | | | | | | | | | | | | | | | | | | | | | | | | | | | |  | | | | | | | | | | ^42^ | | |
|  |  | | | | | | | | | | | | | 64.4 | | | | | | | | | | | | | | | | | | |  | | | | | | | | | | | | | | | | | | | | | | | | | | | |  | | | | | | | | | | ^88^ | | |
|  |  | | | | | | | | | | | | | 51 | | | | | | | | | | | | | | | | | | |  | | | | | | | | | | | | | | | | | | | | | | | | | | | |  | | | | | | | | | | ^92^ | | |
|  |  | | | | | | | | | | | | | 45.2 (18.3) | | | | | | | | | | | | | | | | | | |  | | | | | | | | | | | | | | | | | | | | | | | | | | | |  | | | | | | | | | | ^80^ | | |
|  |  | | | | | | | | | | | | | 17.4(Treatment) | | | | | | | | | | | | | | | | | | |  | | | | | | | | | | | | | | | | | | | | | | | | | | | |  | | | | | | | | | | ^75^ | | |
|  |  | | | | | | | | | | | | | 13.1 (Control) | | | | | | | | | | | | | | | | | | |  | | | | | | | | | | | | | | | | | | | | | | | | | | | |  | | | | | | | | | |  | | |
|  |  | | | | | | | | | | | | |  | | | | | | | | | | | | | | | | | | | 19 | | | | | | | | | | | | | | | | | | | | | | | | | | | |  | | | | | | | | | | ^58^ | | |
|  |  | | | | | | | | | | | | |  | | | | | | | | | | | | | | | | | | | 18.8 | | | | | | | | | | | | | | | | | | | | | | | | | | | |  | | | | | | | | | | ^57^ | | |
|  |  | | | | | | | | | | | | | 14-346 (Time frame varies) | | | | | | | | | | | | | | | | | | |  | | | | | | | | | | | | | | | | | | | | | | | | | | | |  | | | | | | | | | | ^31^ | | |
|  | 60.4 | | | | | | | | | | | | |  | | | | | | | | | | | | | | | | | | |  | | | | | | | | | | | | | | | | | | | | | | | | | | | | Home care | | | | | | | | | | ^82^ | | |
|  | 29.7 | | | | | | | | | | | | |  | | | | | | | | | | | | | | | | | | |  | | | | | | | | | | | | | | | | | | | | | | | | | | | | Hospice | | | | | | | | | |  | | |
| **Factors related to turnover** | **Community characteristics** | | | | | | | | | | | | | | | | | | | | | | | | | | | | | | | | | | | | | | | | | | | | | | | | |  | | | | | | | | | | | | | | | | | | | | |  | | |
|  | High Unemployment rate | | | | | | | | | | | | | | | | | | | | | | | | | | | | | | | | | | | | | | | | | | | | | | | | | —  NS | | | | | | | | | | | | | | | | | | | | | ^57,82,91^  ^83,92^ | | |
|  | Potential alternate employers | | | | | | | | | | | | | | | | | | | | | | | | | | | | | | | | | | | | | | | | | | | | | | | | | + | | | | | | | | | | | | | | | | | | | | | ^80^ | | |
|  | Lack of career pathway | | | | | | | | | | | | | | | | | | | | | | | | | | | | | | | | | | | | | | | | | | | | | | | | | + | | | | | | | | | | | | | | | | | | | | | ^28^ | | |
|  | Job insecurity | | | | | | | | | | | | | | | | | | | | | | | | | | | | | | | | | | | | | | | | | | | | | | | | | + | | | | | | | | | | | | | | | | | | | | | ^44^ | | |
|  | Located in an unattractive neighbourhood | | | | | | | | | | | | | | | | | | | | | | | | | | | | | | | | | | | | | | | | | | | | | | | | | + | | | | | | | | | | | | | | | | | | | | | ^93^ | | |
|  | Longer distance from work | | | | | | | | | | | | | | | | | | | | | | | | | | | | | | | | | | | | | | | | | | | | | | | | | + | | | | | | | | | | | | | | | | | | | | | ^80^ | | |
|  | Per capita income in the county | | | | | | | | | | | | | | | | | | | | | | | | | | | | | | | | | | | | | | | | | | | | | | | | | —  NS | | | | | | | | | | | | | | | | | | | | | ^57^  ^92^ | | |
|  | **General facility characteristics** | | | | | | | | | | | | | | | | | | | | | | | | | | | | | | | | | | | | | | | | | | | | | | | | |  | | | | | | | | | | | | | | | | | | | | |  | | |
|  | For-profit status | | | | | | | | | | | | | | | | | | | | | | | | | | | | | | | | | | | | | | | | | | | | | | | | | +  + (Voluntary) | | | | | | | | | | | | | | | | | | | | | ^58,82,83,87,94^  ^82^ | | |
|  | Facility is investor-owned/privately owned | | | | | | | | | | | | | | | | | | | | | | | | | | | | | | | | | | | | | | | | | | | | | | | | | + | | | | | | | | | | | | | | | | | | | | | ^92^ | | |
|  | Change in ownership | | | | | | | | | | | | | | | | | | | | | | | | | | | | | | | | | | | | | | | | | | | | | | | | | NS | | | | | | | | | | | | | | | | | | | | | ^83^ | | |
|  | Larger facility size/more beds | | | | | | | | | | | | | | | | | | | | | | | | | | | | | | | | | | | | | | | | | | | | | | | | | +  — | | | | | | | | | | | | | | | | | | | | | ^57,88,93^  ^58,80^ | | |
|  | Smaller facility size | | | | | | | | | | | | | | | | | | | | | | | | | | | | | | | | | | | | | | | | | | | | | | | | | NS | | | | | | | | | | | | | | | | | | | | | ^92^ | | |
|  | Chain membership | | | | | | | | | | | | | | | | | | | | | | | | | | | | | | | | | | | | | | | | | | | | | | | | | +  —  NS | | | | | | | | | | | | | | | | | | | | | ^94^  ^80^  ^82^ | | |
|  | Non-chain facility | | | | | | | | | | | | | | | | | | | | | | | | | | | | | | | | | | | | | | | | | | | | | | | | | — | | | | | | | | | | | | | | | | | | | | | ^93^ | | |
|  | Facility is a training site | | | | | | | | | | | | | | | | | | | | | | | | | | | | | | | | | | | | | | | | | | | | | | | | | + | | | | | | | | | | | | | | | | | | | | | ^92^ | | |
|  | Combination Facility | | | | | | | | | | | | | | | | | | | | | | | | | | | | | | | | | | | | | | | | | | | | | | | | | NS | | | | | | | | | | | | | | | | | | | | | ^83^ | | |
|  | Region of Facility | | | | | | | | | | | | | | | | | | | | | | | | | | | | | | | | | | | | | | | | | | | | | | | | | NS | | | | | | | | | | | | | | | | | | | | | ^83^ | | |
|  | **Staffing** | | | | | | | | | | | | | | | | | | | | | | | | | | | | | | | | | | | | | | | | | | | | | | | | |  | | | | | | | | | | | | | | | | | | | | |  | | |
|  | High RN staffing  High LPN staffing  Low RN turnover | | | | | | | | | | | | | | | | | | | | | | | | | | | | | | | | | | | | | | | | | | | | | | | | | —  —  — | | | | | | | | | | | | | | | | | | | | | ^88^  ^80,91^  ^92^ | | |
|  | Low RN staffing  High RN turnover | | | | | | | | | | | | | | | | | | | | | | | | | | | | | | | | | | | | | | | | | | | | | | | | | +  + | | | | | | | | | | | | | | | | | | | | | ^88^  ^92^ | | |
|  | Greater NA HPPD | | | | | | | | | | | | | | | | | | | | | | | | | | | | | | | | | | | | | | | | | | | | | | | | | — | | | | | | | | | | | | | | | | | | | | | ^57,58,86^ | | |
|  | Percentage of PT workers | | | | | | | | | | | | | | | | | | | | | | | | | | | | | | | | | | | | | | | | | | | | | | | | | NS | | | | | | | | | | | | | | | | | | | | | ^82^ | | |
|  | OT shifts | | | | | | | | | | | | | | | | | | | | | | | | | | | | | | | | | | | | | | | | | | | | | | | | | NS | | | | | | | | | | | | | | | | | | | | | ^57^ | | |
|  | **Remuneration** | | | | | | | | | | | | | | | | | | | | | | | | | | | | | | | | | | | | | | | | | | | | | | | | |  | | | | | | | | | | | | | | | | | | | | |  | | |
|  | High/higher NA wages or income | | | | | | | | | | | | | | | | | | | | | | | | | | | | | | | | | | | | | | | | | | | | | | | | | —  NS | | | | | | | | | | | | | | | | | | | | | ^58,72,85, 87, 94^  ^82^ | | |
|  | Lower/lowest NA wages | | | | | | | | | | | | | | | | | | | | | | | | | | | | | | | | | | | | | | | | | | | | | | | | | +  NS | | | | | | | | | | | | | | | | | | | | | ^28^  ^82^ | | |
|  | Comparable income | | | | | | | | | | | | | | | | | | | | | | | | | | | | | | | | | | | | | | | | | | | | | | | | | NS | | | | | | | | | | | | | | | | | | | | | ^83^ | | |
|  | Higher administrative expenses | | | | | | | | | | | | | | | | | | | | | | | | | | | | | | | | | | | | | | | | | | | | | | | | | — | | | | | | | | | | | | | | | | | | | | | ^85,87^ | | |
|  | Activity costs (e.g. resident activities) | | | | | | | | | | | | | | | | | | | | | | | | | | | | | | | | | | | | | | | | | | | | | | | | | — | | | | | | | | | | | | | | | | | | | | | ^87^ | | |
|  | **Benefits**  Provision of health insurance benefits | | | | | | | | | | | | | | | | | | | | | | | | | | | | | | | | | | | | | | | | | | | | | | | | | —  NS | | | | | | | | | | | | | | | | | | | | | ^58,72^  ^22,93^ | | |
|  | No health benefits offered | | | | | | | | | | | | | | | | | | | | | | | | | | | | | | | | | | | | | | | | | | | | | | | | | NS (overall turnover) | | | | | | | | | | | | | | | | | | | | | ^82^ | | |
|  | Employee assistance benefits | | | | | | | | | | | | | | | | | | | | | | | | | | | | | | | | | | | | | | | | | | | | | | | | | — | | | | | | | | | | | | | | | | | | | | | ^58^ | | |
|  | Union contract in place/union membership | | | | | | | | | | | | | | | | | | | | | | | | | | | | | | | | | | | | | | | | | | | | | | | | | — | | | | | | | | | | | | | | | | | | | | | ^58,92^ | | |
|  | **Administrative** | | | | | | | | | | | | | | | | | | | | | | | | | | | | | | | | | | | | | | | | | | | | | | | | |  | | | | | | | | | | | | | | | | | | | | |  | | |
|  | Managers who do not solicit input | | | | | | | | | | | | | | | | | | | | | | | | | | | | | | | | | | | | | | | | | | | | | | | | | + | | | | | | | | | | | | | | | | | | | | | ^91^ | | |
|  | Managers who solicit input | | | | | | | | | | | | | | | | | | | | | | | | | | | | | | | | | | | | | | | | | | | | | | | | | — | | | | | | | | | | | | | | | | | | | | |  | | |
|  | Flatter management structure | | | | | | | | | | | | | | | | | | | | | | | | | | | | | | | | | | | | | | | | | | | | | | | | | — | | | | | | | | | | | | | | | | | | | | | ^92^ | | |
|  | Supervisor trained in management | | | | | | | | | | | | | | | | | | | | | | | | | | | | | | | | | | | | | | | | | | | | | | | | | — | | | | | | | | | | | | | | | | | | | | |  | | |
|  | **Resident Care** | | | | | | | | | | | | | | | | | | | | | | | | | | | | | | | | | | | | | | | | | | | | | | | | |  | | | | | | | | | | | | | | | | | | | | |  | | |
|  | High quality of care | | | | | | | | | | | | | | | | | | | | | | | | | | | | | | | | | | | | | | | | | | | | | | | | | — | | | | | | | | | | | | | | | | | | | | | ^80^ | | |
|  | Poor/lower quality of care at the facility | | | | | | | | | | | | | | | | | | | | | | | | | | | | | | | | | | | | | | | | | | | | | | | | | + | | | | | | | | | | | | | | | | | | | | | ^88,89^ | | |
|  | NA involvement in resident care planning | | | | | | | | | | | | | | | | | | | | | | | | | | | | | | | | | | | | | | | | | | | | | | | | | — | | | | | | | | | | | | | | | | | | | | | ^58^ | | |
|  | Patient demand on clinical time | | | | | | | | | | | | | | | | | | | | | | | | | | | | | | | | | | | | | | | | | | | | | | | | | NS | | | | | | | | | | | | | | | | | | | | | ^92^ | | |
|  | Moderate occupancy | | | | | | | | | | | | | | | | | | | | | | | | | | | | | | | | | | | | | | | | | | | | | | | | | — | | | | | | | | | | | | | | | | | | | | | ^58^ | | |
|  | High Medicaid occupancy | | | | | | | | | | | | | | | | | | | | | | | | | | | | | | | | | | | | | | | | | | | | | | | | | + | | | | | | | | | | | | | | | | | | | | | ^88^ | | |
| **Recruitment** | **How CNAs found job** | | | | | | | | | | | | |  | | | | | | | | | | | | | | | | | | | **Immigrant** | | | | | | | | | | | | | | | | | | | | | | | | | | | | **Non-immigrant** | | | | | | | | | |  | | |
| Mean % | Advertisement | | | | | | | | | | | | | 39.2 | | | | | | | | | | | | | | | | | | |  | | | | | | | | | | | | | | | | | | | | | | | | | | | |  | | | | | | | | | | ^40^ | | |
|  | Newspaper | | | | | | | | | | | | | 23.0* | | | | | | | | | | | | | | | | | | | 15.7 | | | | | | | | | | | | | | | | | | | | | | | | | | | | 24.7 | | | | | | | | | | ^33^ | | |
|  | Family/friend | | | | | | | | | | | | | 45.2*  27.8 | | | | | | | | | | | | | | | | | | | 59.1 | | | | | | | | | | | | | | | | | | | | | | | | | | | | 41.6 | | | | | | | | | | ^33^  ^40^ | | |
|  | School or training program | | | | | | | | | | | | | 3.8  6.1 | | | | | | | | | | | | | | | | | | | 5.6 | | | | | | | | | | | | | | | | | | | | | | | | | | | | 6.3 | | | | | | | | | | ^40^  ^33^ | | |
|  | Other  Approached Employer | | | | | | | | | | | | | 34.4*  3.8  2.7 | | | | | | | | | | | | | | | | | | | 27.2 | | | | | | | | | | | | | | | | | | | | | | | | | | | | 36.3 | | | | | | | | | | ^33^  ^40^ | | |
|  |  | | | | | | | | | | | | | Employers primarily find foreign direct care workers by word of mouth | | | | | | | | | | | | | | | | | | | | | | | | | | | | | | | | | | | | | | | | | | | | | | | | | | | | | | | | | ^16^ | | |
| **Themes that attract workers to home support** | - Caring and sharing – “sociable” personality, desire to work with people - Experience and Exposure – helping relative or friend - Finances and Flexibility – wages, affordable and timely training, training bursaries, provision of benefits, flexible scheduling   *In BC wages and benefits were major attractors to the field. In ON and NS, wages and benefits were main challenge | | | | | | | | | | | | | | | | | | | | | | | | | | | | | | | | | | | | | | | | | | | | | | | | | | | | | | | | | | | | | | | | | | | | | | ^41^ | | |
|  | - Relevant knowledge base, competency in English of international students and local experience that may help students become permanent residents also contribute to attraction of migrant workers to LTC jobs | | | | | | | | | | | | | | | | | | | | | | | | | | | | | | | | | | | | | | | | | | | | | | | | | | | | | | | | | | | | | | | | | | | | | | ^15^ | | |
| **Agency Requirements to Hire** | Request references | | | | | | | | | | | | | | | | | | | | | | | | | | | | | | | | | | | | | | | | | | | | | | | | | 63.6 | | | | | | | | | | | | | | | | | | | | | ^40^ | | |
|  | Must have experience | | | | | | | | | | | | | | | | | | | | | | | | | | | | | | | | | | | | | | | | | | | | | | | | | 67.2 | | | | | | | | | | | | | | | | | | | | |  | | |
|  | Test of basic knowledge | | | | | | | | | | | | | | | | | | | | | | | | | | | | | | | | | | | | | | | | | | | | | | | | | 16.5 | | | | | | | | | | | | | | | | | | | | |  | | |
|  | CNA training required | | | | | | | | | | | | | | | | | | | | | | | | | | | | | | | | | | | | | | | | | | | | | | | | | 6.7 | | | | | | | | | | | | | | | | | | | | |  | | |
|  | CNA training preferred | | | | | | | | | | | | | | | | | | | | | | | | | | | | | | | | | | | | | | | | | | | | | | | | | 18.9 | | | | | | | | | | | | | | | | | | | | |  | | |
|  | State criminal background check | | | | | | | | | | | | | | | | | | | | | | | | | | | | | | | | | | | | | | | | | | | | | | | | | 91.9 | | | | | | | | | | | | | | | | | | | | |  | | |
|  | Federal criminal background check | | | | | | | | | | | | | | | | | | | | | | | | | | | | | | | | | | | | | | | | | | | | | | | | | 55.8 | | | | | | | | | | | | | | | | | | | | |  | | |
|  | Reference check | | | | | | | | | | | | | | | | | | | | | | | | | | | | | | | | | | | | | | | | | | | | | | | | | 62.2 | | | | | | | | | | | | | | | | | | | | |  | | |
|  | English language | | | | | | | | | | | | | | | | | | | | | | | | | | | | | | | | | | | | | | | | | | | | | | | | | 67.8 | | | | | | | | | | | | | | | | | | | | |  | | |
|  | Driving license | | | | | | | | | | | | | | | | | | | | | | | | | | | | | | | | | | | | | | | | | | | | | | | | | 6.7 | | | | | | | | | | | | | | | | | | | | |  | | |
|  | Driving record check | | | | | | | | | | | | | | | | | | | | | | | | | | | | | | | | | | | | | | | | | | | | | | | | | 28.4 | | | | | | | | | | | | | | | | | | | | |  | | |
|  | Drug screening | | | | | | | | | | | | | | | | | | | | | | | | | | | | | | | | | | | | | | | | | | | | | | | | | 31.1 | | | | | | | | | | | | | | | | | | | | |  | | |
|  | Random drug screening | | | | | | | | | | | | | | | | | | | | | | | | | | | | | | | | | | | | | | | | | | | | | | | | | 8.3 | | | | | | | | | | | | | | | | | | | | |  | | |
|  | Citizenship or visa verified | | | | | | | | | | | | | | | | | | | | | | | | | | | | | | | | | | | | | | | | | | | | | | | | | 6.7 | | | | | | | | | | | | | | | | | | | | |  | | |
| **Individual**  **Tenure Factors** | Job security is reason for being an NA | | | | | | | | | | | | | | | | | | | | | | | | | | | | | | | | | | | | | | | | | | | | | | | | | + | | | | | | | | | | | | | | | | | | | | | ^56^ | | |
|  | Age >45  Age <30 | | | | | | | | | | | | | | | | | | | | | | | | | | | | | | | | | | | | | | | | | | | | | | | | | +  — | | | | | | | | | | | | | | | | | | | | |  | | |
|  | Male gender | | | | | | | | | | | | | | | | | | | | | | | | | | | | | | | | | | | | | | | | | | | | | | | | | — | | | | | | | | | | | | | | | | | | | | |  | | |
|  | > High school education/GED | | | | | | | | | | | | | | | | | | | | | | | | | | | | | | | | | | | | | | | | | | | | | | | | | — | | | | | | | | | | | | | | | | | | | | |  | | |
|  | Minority race | | | | | | | | | | | | | | | | | | | | | | | | | | | | | | | | | | | | | | | | | | | | | | | | | NS | | | | | | | | | | | | | | | | | | | | |  | | |
|  | English as second language | | | | | | | | | | | | | | | | | | | | | | | | | | | | | | | | | | | | | | | | | | | | | | | | | NS | | | | | | | | | | | | | | | | | | | | |  | | |
| **Turnover Factors** | Increasing age | | | | | | | | | | | | | | | | | | | | | | | | | | | | | | | | | | | | | | | | | | | | | | | | | NS  — | | | | | | | | | | | | | | | | | | | | | ^80^  ^22,94^ | | |
|  | Race and ethnicity  White  Black  Other  Hispanic | | | | | | | | | | | | | | | | | | | | | | | | | | | | | | | | | | | | | | | | | | | | | | | | | (OR 1.00)  NS (OR 1.19)  NS (OR 0.58)  + (OR 1.71)  NS | | | | | | | | | | | | | | | | | | | | | ^22^  ^94^ | | |
|  | Racial minority | | | | | | | | | | | | | | | | | | | | | | | | | | | | | | | | | | | | | | | | | | | | | | | | | — | | | | | | | | | | | | | | | | | | | | | ^80^ | | |
|  | English as primary language | | | | | | | | | | | | | | | | | | | | | | | | | | | | | | | | | | | | | | | | | | | | | | | | | — | | | | | | | | | | | | | | | | | | | | | ^94^ | | |
|  | Marital status | | | | | | | | | | | | | | | | | | | | | | | | | | | | | | | | | | | | | | | | | | | | | | | | | +  NS (compared to HHAs) | | | | | | | | | | | | | | | | | | | | | ^80^  ^22^ | | |
|  | Have children under 18 years | | | | | | | | | | | | | | | | | | | | | | | | | | | | | | | | | | | | | | | | | | | | | | | | | — | | | | | | | | | | | | | | | | | | | | | ^22^ | | |
|  | Longer tenure in profession | | | | | | | | | | | | | | | | | | | | | | | | | | | | | | | | | | | | | | | | | | | | | | | | | + | | | | | | | | | | | | | | | | | | | | | ^80^ | | |
|  | High school or less | | | | | | | | | | | | | | | | | | | | | | | | | | | | | | | | | | | | | | | | | | | | | | | | | — | | | | | | | | | | | | | | | | | | | | | ^94^ | | |
|  | Any college | | | | | | | | | | | | | | | | | | | | | | | | | | | | | | | | | | | | | | | | | | | | | | | | | NS | | | | | | | | | | | | | | | | | | | | | ^22^ | | |
|  | More training for job | | | | | | | | | | | | | | | | | | | | | | | | | | | | | | | | | | | | | | | | | | | | | | | | | + | | | | | | | | | | | | | | | | | | | | | ^80^ | | |
|  | Inadequate training | | | | | | | | | | | | | | | | | | | | | | | | | | | | | | | | | | | | | | | | | | | | | | | | | + | | | | | | | | | | | | | | | | | | | | | ^28^ | | |
|  | High number of previous jobs | | | | | | | | | | | | | | | | | | | | | | | | | | | | | | | | | | | | | | | | | | | | | | | | | + | | | | | | | | | | | | | | | | | | | | | ^80^ | | |
|  | Number of injuries | | | | | | | | | | | | | | | | | | | | | | | | | | | | | | | | | | | | | | | | | | | | | | | | | + | | | | | | | | | | | | | | | | | | | | | ^94^ | | |
|  | Lower Psychological Well-being | | | | | | | | | | | | | | | | | | | | | | | | | | | | | | | | | | | | | | | | | | | | | | | | | + | | | | | | | | | | | | | | | | | | | | | ^65^ | | |
|  | High Job satisfaction | | | | | | | | | | | | | | | | | | | | | | | | | | | | | | | | | | | | | | | | | | | | | | | | | — | | | | | | | | | | | | | | | | | | | | | ^80^ | | |
|  | Employed in long-term care | | | | | | | | | | | | | | | | | | | | | | | | | | | | | | | | | | | | | | | | | | | | | | | | | + | | | | | | | | | | | | | | | | | | | | | ^77^ | | |
|  | Employed in hospital | | | | | | | | | | | | | | | | | | | | | | | | | | | | | | | | | | | | | | | | | | | | | | | | | + | | | | | | | | | | | | | | | | | | | | |  | | |
|  | Amount of workload/time to do job | | | | | | | | | | | | | | | | | | | | | | | | | | | | | | | | | | | | | | | | | | | | | | | | | + | | | | | | | | | | | | | | | | | | | | | ^80^ | | |
|  | Hours worked per week | | | | | | | | | | | | | | | | | | | | | | | | | | | | | | | | | | | | | | | | | | | | | | | | | NS | | | | | | | | | | | | | | | | | | | | | ^22^ | | |
| **Intent to Leave**  Mean % |  | | | | | | | | | | | | | 43 | | | | | | | | | | | | | | | | | | |  | | | | | | | | | | | | | | | | | | | | | | | | | | | |  | | | | | | | | | | ^55^ | | |
|  | Citizenship status | | | | | | | | | | | | | **US-born** | | | | | | | | | | | | | | | | | | | **Naturalized** | | | | | | | | | | | | | | | | | | | | | | | | | | | | **Non-US citizen** | | | | | | | | | |  | | |
|  |  | | | | | | | | | | | | | 43.4 | | | | | | | | | | | | | | | | | | | 45.3 | | | | | | | | | | | | | | | | | | | | | | | | | | | | 61.0*** | | | | | | | | | | ^54^ | | |
|  | Age | | | | | | | | | | | | | **<30 years**  57.0 *** | | | | | | | | | | | | | | | | | | | **30-49 years**  43.1 ** | | | | | | | | | | | | | | | | | | | | | | | | | | | | **≥50 years**  33.8 | | | | | | | | | |  | | |
| **Intent to Leave Factors** | Younger | | | | | | | | | | | | | | | | | | | | | | | | | | | | | | | | | | | | | | | | | | | | | | | | | + | | | | | | | | | | | | | | | | | | | | | ^54,67,68^ | | |
|  | Older | | | | | | | | | | | | | | | | | | | | | | | | | | | | | | | | | | | | | | | | | | | | | | | | | — | | | | | | | | | | | | | | | | | | | | | ^72^ | | |
|  | Female | | | | | | | | | | | | | | | | | | | | | | | | | | | | | | | | | | | | | | | | | | | | | | | | | +  — | | | | | | | | | | | | | | | | | | | | | ^68^  ^67^ | | |
|  | Unmarried | | | | | | | | | | | | | | | | | | | | | | | | | | | | | | | | | | | | | | | | | | | | | | | | | + | | | | | | | | | | | | | | | | | | | | |  | | |
|  | Did Overtime | | | | | | | | | | | | | | | | | | | | | | | | | | | | | | | | | | | | | | | | | | | | | | | | | + | | | | | | | | | | | | | | | | | | | | | ^67^ | | |
|  | Night shifts | | | | | | | | | | | | | | | | | | | | | | | | | | | | | | | | | | | | | | | | | | | | | | | | | + | | | | | | | | | | | | | | | | | | | | | ^67,68^ | | |
|  | Amount of workload/time to do job | | | | | | | | | | | | | | | | | | | | | | | | | | | | | | | | | | | | | | | | | | | | | | | | | + | | | | | | | | | | | | | | | | | | | | | ^80^ | | |
|  | Overload | | | | | | | | | | | | | | | | | | | | | | | | | | | | | | | | | | | | | | | | | | | | | | | | | + | | | | | | | | | | | | | | | | | | | | | ^79^ | | |
|  | High level of stress | | | | | | | | | | | | | | | | | | | | | | | | | | | | | | | | | | | | | | | | | | | | | | | | | + | | | | | | | | | | | | | | | | | | | | | ^32^ | | |
|  | Low education | | | | | | | | | | | | | | | | | | | | | | | | | | | | | | | | | | | | | | | | | | | | | | | | | + | | | | | | | | | | | | | | | | | | | | | ^68^ | | |
|  | >High school | | | | | | | | | | | | | | | | | | | | | | | | | | | | | | | | | | | | | | | | | | | | | | | | | + | | | | | | | | | | | | | | | | | | | | | ^49,79^ | | |
|  | Some college or more | | | | | | | | | | | | | | | | | | | | | | | | | | | | | | | | | | | | | | | | | | | | | | | | | + | | | | | | | | | | | | | | | | | | | | | ^77^ | | |
|  | Workers with trade degrees | | | | | | | | | | | | | | | | | | | | | | | | | | | | | | | | | | | | | | | | | | | | | | | | | + | | | | | | | | | | | | | | | | | | | | | ^72^ | | |
|  | Had national qualification | | | | | | | | | | | | | | | | | | | | | | | | | | | | | | | | | | | | | | | | | | | | | | | | | + | | | | | | | | | | | | | | | | | | | | | ^67^ | | |
|  | 2+ jobs in past 5 years | | | | | | | | | | | | | | | | | | | | | | | | | | | | | | | | | | | | | | | | | | | | | | | | | + | | | | | | | | | | | | | | | | | | | | | ^49,79^ | | |
|  | Having other jobs | | | | | | | | | | | | | | | | | | | | | | | | | | | | | | | | | | | | | | | | | | | | | | | | | NS | | | | | | | | | | | | | | | | | | | | | ^67^ | | |
|  | Number of previous positions as NA | | | | | | | | | | | | | | | | | | | | | | | | | | | | | | | | | | | | | | | | | | | | | | | | | + | | | | | | | | | | | | | | | | | | | | | ^67,80^ | | |
|  | Training | | | | | | | | | | | | | | | | | | | | | | | | | | | | | | | | | | | | | | | | | | | | | | | | | +  — | | | | | | | | | | | | | | | | | | | | | ^80^  ^32^ | | |
|  | Worked in facility care | | | | | | | | | | | | | | | | | | | | | | | | | | | | | | | | | | | | | | | | | | | | | | | | | + | | | | | | | | | | | | | | | | | | | | | ^67^ | | |
|  | Worked in LTC 3- <5 years | | | | | | | | | | | | | | | | | | | | | | | | | | | | | | | | | | | | | | | | | | | | | | | | | + | | | | | | | | | | | | | | | | | | | | |  | | |
|  | Worked in LTC for <1 year | | | | | | | | | | | | | | | | | | | | | | | | | | | | | | | | | | | | | | | | | | | | | | | | | — | | | | | | | | | | | | | | | | | | | | |  | | |
|  | Tenure ≥ 7 years | | | | | | | | | | | | | | | | | | | | | | | | | | | | | | | | | | | | | | | | | | | | | | | | | — | | | | | | | | | | | | | | | | | | | | |  | | |
|  | Team Spirit | | | | | | | | | | | | | | | | | | | | | | | | | | | | | | | | | | | | | | | | | | | | | | | | | — | | | | | | | | | | | | | | | | | | | | | ^79^ | | |
|  | High Job satisfaction | | | | | | | | | | | | | | | | | | | | | | | | | | | | | | | | | | | | | | | | | | | | | | | | | — | | | | | | | | | | | | | | | | | | | | | ^49,80^ | | |
|  | High job security | | | | | | | | | | | | | | | | | | | | | | | | | | | | | | | | | | | | | | | | | | | | | | | | | — | | | | | | | | | | | | | | | | | | | | | ^56^ | | |
|  | Low job security | | | | | | | | | | | | | | | | | | | | | | | | | | | | | | | | | | | | | | | | | | | | | | | | | + | | | | | | | | | | | | | | | | | | | | | ^68^ | | |
|  | Perception of being valued by employer | | | | | | | | | | | | | | | | | | | | | | | | | | | | | | | | | | | | | | | | | | | | | | | | | NS | | | | | | | | | | | | | | | | | | | | | ^49^ | | |
|  | Discrimination | | | | | | | | | | | | | | | | | | | | | | | | | | | | | | | | | | | | | | | | | | | | | | | | | NS | | | | | | | | | | | | | | | | | | | | | ^79^ | | |
|  | Experienced work-related injury within the past year | | | | | | | | | | | | | | | | | | | | | | | | | | | | | | | | | | | | | | | | | | | | | | | | | NS | | | | | | | | | | | | | | | | | | | | | ^49^ | | |
| **Benefits**  Mean % |  | | | | | | | | | | | | | |  | | | | | | | | | | | | | | | | | | | | | | | | | | | | | | | | | | | **Setting** | | | | | | | | | | | | | | | | | | | | |  | | |
| **Health insurance** | None | | | | | | | | | | | | | | 33 | | | | | | | | | | | | | | | | | | | | | | | | | | | | | | | | | | | HH | | | | | | | | | | | | | | | | | | | | | ^82^ | | |
|  |  | | | | | | | | | | | | | | 25 | | | | | | | | | | | | | | | | | | | | | | | | | | | | | | | | | | | Multiple | | | | | | | | | | | | | | | | | | | | | ^22^ | | |
|  |  | | | | | | | | | | | | | | 16.5, 12.7, 17.5 | | | | | | | | | | | | | | | | | | | | | | | | | | | | | | | | | | | NH – Total, Immigrants , Non-Immigrants | | | | | | | | | | | | | | | | | | | | | ^33^ | | |
|  | Available | | | | | | | | | | | | | | 90.9, 91.2, 91.6, 88.5 | | | | | | | | | | | | | | | | | | | | | | | | | | | | | | | | | | | All, Urban, Micropolitan, Rural | | | | | | | | | | | | | | | | | | | | | ^53^ | | |
|  |  | | | | | | | | | | | | | | 89.7 | | | | | | | | | | | | | | | | | | | | | | | | | | | | | | | | | | |  | | | | | | | | | | | | | | | | | | | | | ^24^ | | |
|  |  | | | | | | | | | | | | | | 83.3 | | | | | | | | | | | | | | | | | | | | | | | | | | | | | | | | | | |  | | | | | | | | | | | | | | | | | | | | | ^49^ | | |
|  |  | | | | | | | | | | | | | | 89.6, 91.2 | | | | | | | | | | | | | | | | | | | | | | | | | | | | | | | | | | | Immigrants, Non-Immigrants | | | | | | | | | | | | | | | | | | | | | ^33^ | | |
|  | Have | | | | | | | | | | | | | | 60.6, 52.7 | | | | | | | | | | | | | | | | | | | | | | | | | | | | | | | | | | | Immigrants, Non-Immigrants | | | | | | | | | | | | | | | | | | | | |  | | |
|  |  | | | | | | | | | | | | | | 62.3, 41.8, 25.5 | | | | | | | | | | | | | | | | | | | | | | | | | | | | | | | | | | | H, NH, HH | | | | | | | | | | | | | | | | | | | | | ^39^ | | |
|  |  | | | | | | | | | | | | | | 54.0 | | | | | | | | | | | | | | | | | | | | | | | | | | | | | | | | | | | NH | | | | | | | | | | | | | | | | | | | | | ^24^ | | |
|  |  | | | | | | | | | | | | | | 54.1, 55.2, 49.2, 54.8 | | | | | | | | | | | | | | | | | | | | | | | | | | | | | | | | | | | All, Urban, Micropolitan, Rural | | | | | | | | | | | | | | | | | | | | | ^53^ | | |
|  |  | | | | | | | | | | | | | | 10.4 (Canada) | | | | | | | | | | | | | | | | | | | | | | | | | | | | | | | | | | | HH | | | | | | | | | | | | | | | | | | | | | ^27^ | | |
|  | Public | | | | | | | | | | | | | | 22 | | | | | | | | | | | | | | | | | | | | | | | | | | | | | | | | | | | Multiple | | | | | | | | | | | | | | | | | | | | | ^22^ | | |
|  | Private | | | | | | | | | | | | | | 60 | | | | | | | | | | | | | | | | | | | | | | | | | | | | | | | | | | |  | | | | | | | | | | | | | | | | | | | | |  | | |
|  | Employer-based | | | | | | | | | | | | | | 45.3 | | | | | | | | | | | | | | | | | | | | | | | | | | | | | | | | | | |  | | | | | | | | | | | | | | | | | | | | | ^72^ | | |
|  |  | | | | | | | | | | | | | | 41 | | | | | | | | | | | | | | | | | | | | | | | | | | | | | | | | | | |  | | | | | | | | | | | | | | | | | | | | | ^76^ | | |
|  |  | | | | | | | | | | | | | | 38 | | | | | | | | | | | | | | | | | | | | | | | | | | | | | | | | | | |  | | | | | | | | | | | | | | | | | | | | | ^22^ | | |
|  | Fully paid for employee only | | | | | | | | | | | | | | 12 | | | | | | | | | | | | | | | | | | | | | | | | | | | | | | | | | | | NH | | | | | | | | | | | | | | | | | | | | | ^56^ | | |
|  |  | | | | | | | | | | | | | | 9 | | | | | | | | | | | | | | | | | | | | | | | | | | | | | | | | | | |  | | | | | | | | | | | | | | | | | | | | | ^59^ | | |
|  | Partially paid for  employee only | | | | | | | | | | | | | | 79 | | | | | | | | | | | | | | | | | | | | | | | | | | | | | | | | | | |  | | | | | | | | | | | | | | | | | | | | | ^54^ | | |
|  |  | | | | | | | | | | | | | | 69 | | | | | | | | | | | | | | | | | | | | | | | | | | | | | | | | | | |  | | | | | | | | | | | | | | | | | | | | | ^58^ | | |
|  |  | | | | | | | | | | | | | | 19 | | | | | | | | | | | | | | | | | | | | | | | | | | | | | | | | | | |  | | | | | | | | | | | | | | | | | | | | | ^59^ | | |
|  | Fully paid for employee and family | | | | | | | | | | | | | | 4 | | | | | | | | | | | | | | | | | | | | | | | | | | | | | | | | | | |  | | | | | | | | | | | | | | | | | | | | | ^56^ | | |
|  |  | | | | | | | | | | | | | | 3 | | | | | | | | | | | | | | | | | | | | | | | | | | | | | | | | | | |  | | | | | | | | | | | | | | | | | | | | | ^59^ | | |
|  | Partially paid for employee and family | | | | | | | | | | | | | | 64 | | | | | | | | | | | | | | | | | | | | | | | | | | | | | | | | | | |  | | | | | | | | | | | | | | | | | | | | | ^56^ | | |
|  |  | | | | | | | | | | | | | | 56 | | | | | | | | | | | | | | | | | | | | | | | | | | | | | | | | | | |  | | | | | | | | | | | | | | | | | | | | | ^59^ | | |
|  | Drug | | | | | | | | | | | | | | 8.6 | | | | | | | | | | | | | | | | | | | | | | | | | | | | | | | | | | | HH | | | | | | | | | | | | | | | | | | | | | ^27^ | | |
|  | Dental | | | | | | | | | | | | | | 7.1 | | | | | | | | | | | | | | | | | | | | | | | | | | | | | | | | | | |  | | | | | | | | | | | | | | | | | | | | |  | | |
|  | Job protection during maternity leave | | | | | | | | | | | | | | <40 | | | | | | | | | | | | | | | | | | | | | | | | | | | | | | | | | | |  | | | | | | | | | | | | | | | | | | | | | ^44^ | | |
| **Pension Plan** | EAP Access | | | | | | | | | | | | | | 30 | | | | | | | | | | | | | | | | | | | | | | | | | | | | | | | | | | | NH | | | | | | | | | | | | | | | | | | | | | ^58^ | | |
|  | Have | | | | | | | | | | | | | | 67.5, 67.8, 71.2, 61.3 | | | | | | | | | | | | | | | | | | | | | | | | | | | | | | | | | | | All, Urban, Micropolitan, Rural | | | | | | | | | | | | | | | | | | | | | ^53^ | | |
|  |  | | | | | | | | | | | | | | 66.9, 53.8, 65.4 | | | | | | | | | | | | | | | | | | | | | | | | | | | | | | | | | | | H, NH, HH | | | | | | | | | | | | | | | | | | | | | ^39^ | | |
|  |  | | | | | | | | | | | | | | 65.2, 68.2 | | | | | | | | | | | | | | | | | | | | | | | | | | | | | | | | | | | NH- Immigrants, Non-Immigrants | | | | | | | | | | | | | | | | | | | | | ^33^ | | |
|  |  | | | | | | | | | | | | | | 62 | | | | | | | | | | | | | | | | | | | | | | | | | | | | | | | | | | |  | | | | | | | | | | | | | | | | | | | | | ^56^ | | |
|  |  | | | | | | | | | | | | | | 60 | | | | | | | | | | | | | | | | | | | | | | | | | | | | | | | | | | |  | | | | | | | | | | | | | | | | | | | | | ^58^ | | |
|  |  | | | | | | | | | | | | | | ~33 | | | | | | | | | | | | | | | | | | | | | | | | | | | | | | | | | | | HH | | | | | | | | | | | | | | | | | | | | | ^44^ | | |
|  |  | | | | | | | | | | | | | | 9.7 (Canada) | | | | | | | | | | | | | | | | | | | | | | | | | | | | | | | | | | |  | | | | | | | | | | | | | | | | | | | | | ^27^ | | |
|  | Employer | | | | | | | | | | | | | | 71.5, 43.5, 24.1 | | | | | | | | | | | | | | | | | | | | | | | | | | | | | | | | | | | H, NH, HH | | | | | | | | | | | | | | | | | | | | | ^39^ | | |
|  | Employers pay | | | | | | | | | | | | | |  | | | | | | | | | | | | | | | | | | | | | | | | | | | | | | | | | | |  | | | | | | | | | | | | | | | | | | | | |  | | |
|  | All | | | | | | | | | | | | | | 21.3, 23.9, 30.9 | | | | | | | | | | | | | | | | | | | | | | | | | | | | | | | | | | | H, NH, HH | | | | | | | | | | | | | | | | | | | | | ^39^ | | |
|  | Part | | | | | | | | | | | | | | 74.1, 67.4, 60.0 | | | | | | | | | | | | | | | | | | | | | | | | | | | | | | | | | | |  | | | | | | | | | | | | | | | | | | | | |  | | |
|  | None | | | | | | | | | | | | | | 4.6, 8.8, 9.1 | | | | | | | | | | | | | | | | | | | | | | | | | | | | | | | | | | |  | | | | | | | | | | | | | | | | | | | | |  | | |
| **Paid Time** | Sick | | | | | | | | | | | | | | 79 | | | | | | | | | | | | | | | | | | | | | | | | | | | | | | | | | | | NH | | | | | | | | | | | | | | | | | | | | | ^58^ | | |
|  |  | | | | | | | | | | | | | | 77.0, 72.4 | | | | | | | | | | | | | | | | | | | | | | | | | | | | | | | | | | | Immigrants, Non-Immigrants | | | | | | | | | | | | | | | | | | | | | ^33^ | | |
|  |  | | | | | | | | | | | | | | 73.4, 75.1, 65.7, 70.9 | | | | | | | | | | | | | | | | | | | | | | | | | | | | | | | | | | | All, Urban, Micropolitan, Rural | | | | | | | | | | | | | | | | | | | | | ^53^ | | |
|  |  | | | | | | | | | | | | | | 70.7 | | | | | | | | | | | | | | | | | | | | | | | | | | | | | | | | | | |  | | | | | | | | | | | | | | | | | | | | | ^24^ | | |
|  |  | | | | | | | | | | | | | | 70 | | | | | | | | | | | | | | | | | | | | | | | | | | | | | | | | | | |  | | | | | | | | | | | | | | | | | | | | | ^56^ | | |
|  |  | | | | | | | | | | | | | | ~33 (Canada) | | | | | | | | | | | | | | | | | | | | | | | | | | | | | | | | | | | HH | | | | | | | | | | | | | | | | | | | | | ^44^ | | |
|  |  | | | | | | | | | | | | | | 7.8 (Canada) | | | | | | | | | | | | | | | | | | | | | | | | | | | | | | | | | | |  | | | | | | | | | | | | | | | | | | | | | ^27^ | | |
|  | Vacation/ holidays/ personal days | | | | | | | | | | | | | | 89 | | | | | | | | | | | | | | | | | | | | | | | | | | | | | | | | | | | NH | | | | | | | | | | | | | | | | | | | | | ^56^ | | |
|  |  | | | | | | | | | | | | | | 81.7, 71.6 | | | | | | | | | | | | | | | | | | | | | | | | | | | | | | | | | | | Immigrants, Non-Immigrants | | | | | | | | | | | | | | | | | | | | | ^33^ | | |
|  |  | | | | | | | | | | | | | | 73.8, 75.6, 68.1, 67.6 | | | | | | | | | | | | | | | | | | | | | | | | | | | | | | | | | | | All, Urban, Micropolitan, Rural | | | | | | | | | | | | | | | | | | | | | ^53^ | | |
|  |  | | | | | | | | | | | | | | 64 | | | | | | | | | | | | | | | | | | | | | | | | | | | | | | | | | | |  | | | | | | | | | | | | | | | | | | | | | ^58^ | | |
|  |  | | | | | | | | | | | | | | 43.2 (Canada) | | | | | | | | | | | | | | | | | | | | | | | | | | | | | | | | | | | HH | | | | | | | | | | | | | | | | | | | | | ^27^ | | |
|  | Maternity /childcare leave | | | | | | | | | | | | | | 1.7 | | | | | | | | | | | | | | | | | | | | | | | | | | | | | | | | | | |  | | | | | | | | | | | | | | | | | | | | |  | | |
|  | For good work | | | | | | | | | | | | | | 8.4 | | | | | | | | | | | | | | | | | | | | | | | | | | | | | | | | | | | NH | | | | | | | | | | | | | | | | | | | | | ^53^ | | |
|  |  | | | | | | | | | | | | | | 14.9, 6.9 | | | | | | | | | | | | | | | | | | | | | | | | | | | | | | | | | | | Immigrants, Non-Immigrants | | | | | | | | | | | | | | | | | | | | | ^33^ | | |
|  | Other | | | | | | | | | | | | | | 85.5, 85.8, 85.0, 84.4 | | | | | | | | | | | | | | | | | | | | | | | | | | | | | | | | | | | Urban, Micropolitan, Rural | | | | | | | | | | | | | | | | | | | | | ^53^ | | |
|  |  | | | | | | | | | | | | | | 84.7, 85.7 | | | | | | | | | | | | | | | | | | | | | | | | | | | | | | | | | | | Immigrants, Non-Immigrants | | | | | | | | | | | | | | | | | | | | | ^33^ | | |
| **Bonuses** | General | | | | | | | | | | | | | | 37.6, 37.6, 39.0, 35.5 | | | | | | | | | | | | | | | | | | | | | | | | | | | | | | | | | | | NH - All, Urban, Micropolitan, Rural | | | | | | | | | | | | | | | | | | | | | ^53^ | | |
|  |  | | | | | | | | | | | | | | 37 | | | | | | | | | | | | | | | | | | | | | | | | | | | | | | | | | | |  | | | | | | | | | | | | | | | | | | | | | ^56^ | | |
|  |  | | | | | | | | | | | | | | 30.1, 39.5 | | | | | | | | | | | | | | | | | | | | | | | | | | | | | | | | | | | Immigrants, Non-Immigrants | | | | | | | | | | | | | | | | | | | | | ^33^ | | |
|  | For working holidays | | | | | | | | | | | | | | 86.8, 84.7 | | | | | | | | | | | | | | | | | | | | | | | | | | | | | | | | | | | Immigrants, Non-Immigrants | | | | | | | | | | | | | | | | | | | | |  | | |
|  |  | | | | | | | | | | | | | | 85.1, 85.5, 82.2, 85.7 | | | | | | | | | | | | | | | | | | | | | | | | | | | | | | | | | | | All, Urban, Micropolitan, Rural | | | | | | | | | | | | | | | | | | | | | ^53^ | | |
|  | Tuition reimbursed | | | | | | | | | | | | | | 76.7, 83.7 | | | | | | | | | | | | | | | | | | | | | | | | | | | | | | | | | | | Immigrants, Non-Immigrants | | | | | | | | | | | | | | | | | | | | | ^33^ | | |
|  |  | | | | | | | | | | | | | | 40.6, 42.7, 38.4, 29.2 | | | | | | | | | | | | | | | | | | | | | | | | | | | | | | | | | | | All, Urban, Micropolitan, Rural | | | | | | | | | | | | | | | | | | | | | ^53^ | | |
|  | Tuition subsidized | | | | | | | | | | | | | | 37.0, 41.5 | | | | | | | | | | | | | | | | | | | | | | | | | | | | | | | | | | | Immigrants, Non-Immigrants | | | | | | | | | | | | | | | | | | | | | ^33^ | | |
|  | Transportation | | | | | | | | | | | | | | 38.7 (Canada) | | | | | | | | | | | | | | | | | | | | | | | | | | | | | | | | | | | HH | | | | | | | | | | | | | | | | | | | | | ^27^ | | |
|  |  | | | | | | | | | | | | | | 5.1, 5.4, 4.0, 3.9 | | | | | | | | | | | | | | | | | | | | | | | | | | | | | | | | | | | NH - All, Urban, Micropolitan, Rural | | | | | | | | | | | | | | | | | | | | | ^53^ | | |
|  |  | | | | | | | | | | | | | | 5 | | | | | | | | | | | | | | | | | | | | | | | | | | | | | | | | | | |  | | | | | | | | | | | | | | | | | | | | | ^59^ | | |
|  | Child Care | | | | | | | | | | | | | | 13.5, 5.5 | | | | | | | | | | | | | | | | | | | | | | | | | | | | | | | | | | | Immigrants, Non-Immigrants | | | | | | | | | | | | | | | | | | | | | ^33^ | | |
|  |  | | | | | | | | | | | | | | 7, 7.6, 6.2, 4.3 | | | | | | | | | | | | | | | | | | | | | | | | | | | | | | | | | | | All, Urban, Micropolitan, Rural | | | | | | | | | | | | | | | | | | | | | ^53^ | | |
|  |  | | | | | | | | | | | | | | 5 | | | | | | | | | | | | | | | | | | | | | | | | | | | | | | | | | | |  | | | | | | | | | | | | | | | | | | | | | ^59^ | | |
| **Factors associated with NA benefits in home support** | Facility size | | | | | | | | | | | | | | | | | | | | | | | | | | | | | | | | | | | | | | | | | | | | | | | | | + | | | | | | | | | | | | | | | | | | | | | ^59^ | | |
|  | Not-for-profit ownership | | | | | | | | | | | | | | | | | | | | | | | | | | | | | | | | | | | | | | | | | | | | | | | | | + | | | | | | | | | | | | | | | | | | | | |  | | |
|  | Occupancy level | | | | | | | | | | | | | | | | | | | | | | | | | | | | | | | | | | | | | | | | | | | | | | | | | + | | | | | | | | | | | | | | | | | | | | |  | | |
|  | Nurse staffing level | | | | | | | | | | | | | | | | | | | | | | | | | | | | | | | | | | | | | | | | | | | | | | | | | + | | | | | | | | | | | | | | | | | | | | |  | | |
|  | Union involvement | | | | | | | | | | | | | | | | | | | | | | | | | | | | | | | | | | | | | | | | | | | | | | | | | + | | | | | | | | | | | | | | | | | | | | |  | | |
|  | Education of nursing home administrator | | | | | | | | | | | | | | | | | | | | | | | | | | | | | | | | | | | | | | | | | | | | | | | | | + | | | | | | | | | | | | | | | | | | | | |  | | |
|  | Home support workers compensation issues: “low wages, lack of wage parity” with those employed in institutions/other jurisdictions, and “limited benefits.” | | | | | | | | | | | | | | | | | | | | | | | | | | | | | | | | | | | | | | | | | | | | | | | | | | | | | | | | | | | | | | | | | | | | | | ^44^ | | |
| **Union Membership**  Mean % Unionized | **Overall** | | | | | | | | | | | | | 10.4 ( of NAs) | | | | | | | | | | | | | | | | | | |  | | | | | | | | | | | | | | | | | | | | | | | | | | | |  | | | | | | | | | | ^77^ | | |
|  | **By Setting** | | | | | | | | | | | | |  | | | | | | | | | | | | | | | | | | | **NH** | | | | | | | | | | | | | | | | | | | | | | | | | | | | **HH** | | | | | | | | | |  | | |
|  |  | | | | | | | | | | | | |  | | | | | | | | | | | | | | | | | | | 15 (of facilities) | | | | | | | | | | | | | | | | | | | | | | | | | | | |  | | | | | | | | | | ^58,59^ | | |
|  |  | | | | | | | | | | | | |  | | | | | | | | | | | | | | | | | | | 19 (of facilities) | | | | | | | | | | | | | | | | | | | | | | | | | | | |  | | | | | | | | | | ^92^ | | |
|  |  | | | | | | | | | | | | |  | | | | | | | | | | | | | | | | | | |  | | | | | | | | | | | | | | | | | | | | | | | | | | | | 38 (of NAs) | | | | | | | | | | ^41^ | | |
| **Early Retirement & Disability** | **Most Significant Hazard Ratios of obtaining Voluntary Early Retirement** | | | | | | | | | | | | | | | | | | | | | | | | | | | | | | | | | | | | | | | | | | | | | | | | | | | | | | | | | | | | | | | | | | | | | |  | | |
|  |  | | | | | | | | | | | | | | | | | | | | | | | | | | | | | | | | | | | | | | | **HR** | | | | | | | | | | | | | | | **95% CI** | | | | | | | | | | | | | | | |  | | |
|  | Low education | | | | | | | | | | | | | | | | | | | | | | | | | | | | | | | | | | | | | | | 3.19 | | | | | | | | | | | | | | | 2.65-3.85 | | | | | | | | | | | | | | | | ^66^ | | |
|  | High job demands | | | | | | | | | | | | | | | | | | | | | | | | | | | | | | | | | | | | | | | 1.28 | | | | | | | | | | | | | | | 1.09-1.5 | | | | | | | | | | | | | | | |  | | |
|  | Inflammatory rheumatic disease | | | | | | | | | | | | | | | | | | | | | | | | | | | | | | | | | | | | | | | 1.76 | | | | | | | | | | | | | | | 1.25-2.48 | | | | | | | | | | | | | | | |  | | |
|  | Cardiovascular disease | | | | | | | | | | | | | | | | | | | | | | | | | | | | | | | | | | | | | | | 1.47 | | | | | | | | | | | | | | | 1.27-1.69 | | | | | | | | | | | | | | | |  | | |
|  | Gastrointestinal Disorders | | | | | | | | | | | | | | | | | | | | | | | | | | | | | | | | | | | | | | | 1.39 | | | | | | | | | | | | | | | 1.1-1.76 | | | | | | | | | | | | | | | |  | | |
|  | **Most Significant Hazard Ratios of obtaining Disability Pension** | | | | | | | | | | | | | | | | | | | | | | | | | | | | | | | | | | | | | | | | | | | | | | | | | | | | | | | | | | | | | | | | | | | | | |  | | |
|  | >90 days of low back pain during the last 12 years | | | | | | | | | | | | | | | | | | | | | | | | | | | | | | | | | | | | | | | 2.27 | | | | | | | | | | | | | | | 1.55-3.34 | | | | | | | | | | | | | | | |  | | |
|  | >30 days of sick leave due to upper-extremity disorders | | | | | | | | | | | | | | | | | | | | | | | | | | | | | | | | | | | | | | | 2.18 | | | | | | | | | | | | | | | 1.08-2.11 | | | | | | | | | | | | | | | |  | | |
|  | >30 days of sick leave due to lower extremity disorders | | | | | | | | | | | | | | | | | | | | | | | | | | | | | | | | | | | | | | | 1.51 | | | | | | | | | | | | | | | 1.51-2.11 | | | | | | | | | | | | | | | |  | | |
|  | Inflammatory rheumatic disease | | | | | | | | | | | | | | | | | | | | | | | | | | | | | | | | | | | | | | | 2.42 | | | | | | | | | | | | | | | 1.67-3.52 | | | | | | | | | | | | | | | |  | | |
|  | Gastrointestinal Disease | | | | | | | | | | | | | | | | | | | | | | | | | | | | | | | | | | | | | | | 1.39 | | | | | | | | | | | | | | | 1.1-1.76 | | | | | | | | | | | | | | | |  | | |
|  | Workers Compensation Case | | | | | | | | | | | | | | | | | | | | | | | | | | | | | | | | | | | | | | | 1.51 | | | | | | | | | | | | | | | 1.23-1.87 | | | | | | | | | | | | | | | |  | | |
|  | **Total Number of Lost working years** | | | | | | | | | | | | | | | | | | | | | | | | **Granted Disability Pension** | | | | | | | | | | | | | | | | | | | | | | | | | | | | | | **Granted Early Retirement** | | | | | | | | | | | | | | | |  | | |
|  | 7472 | | | | | | | | | | | | | | | | | | | | | | | | 3714 | | | | | | | | | | | | | | | | | | | | | | | | | | | | | | 11186 | | | | | | | | | | | | | | | |  | | |
| **Theoretical** | - See paper for model equations 1 and 2. Accounts for employee headcount, year, proportion of unfilled positions, target vacancy rate, average FTE, change in population(growth rate), casual requirement, % of graduates hired from Alberta post-secondary institutions, % employees ending employment with AHS, system shocks (economic recovery and/or capital projects). - Nearly all variables involved some form of estimation/judgement to convert available data into model parameters.   Estimated increase in HCA demand +2.3%/year | | | | | | | | | | | | | | | | | | | | | | | | | | | | | | | | | | | | | | | | | | | | | | | | | | | | | | | | | | | | | | | | | | | | | | ^48^ | | |

| **INJURY AND ILLNESS** | | | | | | | | | | | | | | | | | | | | | | | | | | | | | | | | | | | | | | | | | |
| --- | --- | --- | --- | --- | --- | --- | --- | --- | --- | --- | --- | --- | --- | --- | --- | --- | --- | --- | --- | --- | --- | --- | --- | --- | --- | --- | --- | --- | --- | --- | --- | --- | --- | --- | --- | --- | --- | --- | --- | --- | --- |
| Themes | Outcomes | | | | | | | | | | | | | | | | | | | | | | | | | | | | | | | | | | | | | | | | Ref. |
| **Overall Work** **Related Injury Rate**  Mean (% per year)  Mean # (SD) |  | | 59.44 (nursing homes)  59.12 (nursing homes)  18.5 (home health aides) | | | | | | | | | | | | | | | | | | | | | | | | | | | | | | | | | | | | | | ^49^  ^24^  ^94^ |
|  |  |  | 1.54 (0.18) – per individual  2.63 (0.17)  8.52 (6.10) – per nursing home | | | | | | | | | | | | | | | | | | | | | | | | | | | | | | | | | | | | | | ^51^  ^24^  ^90^ |
| **Rate By Injury Type**  Mean (%)  (% of total injuries) | Overall Injuries | | | | | | | | | MSIs | | | | | | | | | | | | | | | | | | | | 84 | | | | | | | | | | | ^64^ |
|  | Rate of Injuries Among NAs (n=2881) | | | | | | | | | Back injury | | | | | | | | | | | | | | | | | | | | 17.34 | | | | | | | | | | | ^51^ |
|  |  |  |  |  |  |  |  |  |  | Other strains or pulled muscles | | | | | | | | | | | | | | | | | | | | 15.63 | | | | | | | | | | |  |
|  |  |  |  |  |  |  |  |  |  | Human bites | | | | | | | | | | | | | | | | | | | | 11.44 | | | | | | | | | | |  |
|  |  |  |  |  |  |  |  |  |  | Scratches, open wounds, or cuts | | | | | | | | | | | | | | | | | | | | 44.39 | | | | | | | | | | |  |
|  |  |  |  |  |  |  |  |  |  | Black eyes or other bruising | | | | | | | | | | | | | | | | | | | | 16.09 | | | | | | | | | | |  |
|  |  |  |  |  |  |  |  |  |  | Other workplace injuries | | | | | | | | | | | | | | | | | | | | 7.16 | | | | | | | | | | |  |
|  | Injury Rate (per 100 person years) and Adjusted Relative Risk (ARR) of Musculoskeletal Injuries (MSIs) (adjusted for age and gender) (*P< 0.05) | | | | | | | | | | | | | | | | | | | | | | | | | | | | | | | | | | | | | | | | ^62^ |
|  |  | |  | | | | | | **RN** | | | | | | | | | | | | | | | | | | | | **CA** | | | | | | | | | | | |  |
|  |  |  | FT | | | | | | 5.9; ARR 1.00 (ref.) | | | | | | | | | | | | | | | | | | | | 20.8; ARR 1.00 (ref.) | | | | | | | | | | | |  |
|  |  |  | PT | | | | | | 4.6; ARR 0.8 (0.6, 1.0)* | | | | | | | | | | | | | | | | | | | | 19.3; ARR 0.9 (0.6, 1.3) | | | | | | | | | | | |  |
|  |  |  | Casual | | | | | | 4.1; ARR 0.7 (0.5, 0.9)* | | | | | | | | | | | | | | | | | | | | 15.2; ARR 0.6 (0.5, 0.8)* | | | | | | | | | | | |  |
|  | **Injury Type by Setting** | | | | | | | | | | | | | | | | | | | | | | | | | | | | | | | | | | | | | | | | ^60^ |
|  |  | | | | | | | | | | | | | **Acute** | | | | | | | **Community** | | | | | | | | | | | | | **NH** | | | | | | |  |
|  | MSI | | | | | | | | | | | | | 60.8 | | | | | | | 73.3 | | | | | | | | | | | | | 73.0 | | | | | | |  |
|  | Irritation and Allergy | | | | | | | | | | | | | 8.0 | | | | | | | 2.8 | | | | | | | | | | | | | 4.3 | | | | | | |  |
|  | Puncture | | | | | | | | | | | | | 8.2 | | | | | | | 2.8 | | | | | | | | | | | | | 1.4 | | | | | | |  |
|  | Other injuries (burns, cuts, bruise, psychological trauma) | | | | | | | | | | | | | 15.9 | | | | | | | 14.7 | | | | | | | | | | | | | 13.9 | | | | | | |  |
|  | Predominant departments for All Injuries in Acute Care | | | | | | | | | | | | | | | | | | | | | | | | | | | | | | | | | | | | | | | |  |
|  |  | | | | | | | | | | **Rehabilitation and Extended Care** | | | | | | | | | | | | | | | **Infection control and Related Areas** | | | | | | | | | | | | | | |  |
|  | MSI | | | | | | | | | | 43 | | | | | | | | | | | | | | | 19.8 | | | | | | | | | | | | | | |  |
|  | Irritation and Allergy | | | | | | | | | | 19.2 | | | | | | | | | | | | | | | 38.5 | | | | | | | | | | | | | | |  |
|  | Puncture | | | | | | | | | | 12.5 | | | | | | | | | | | | | | | 62.5 | | | | | | | | | | | | | | |  |
|  | Other (burns, cuts, bruise, psychological, trauma) | | | | | | | | | | 43.5 | | | | | | | | | | | | | | | 17.4 | | | | | | | | | | | | | | |  |
| **Rate by Occupation** |  | | | | | | | | | | | | | | | | | **RN** | | | | | | | **LPN** | | | | | | | | | | | | | | **CA** | | ^61^ |
|  | Rate of Time-loss falls/100 FTE (2005-2008) | | | | | | | | | | | | | | | | | 0.9 | | | | | | | 0.9 | | | | | | | | | | | | | | 2.2 | |  |
|  | Crude Odds Ratio of Falls Resulting in Time-Loss | | | | | | | | | | | | | | | | | 1.00 (ref) | | | | | | | 0.88  (0.46-1.71) | | | | | | | | | | | | | | 2.14  (1.38–3.31)** | |  |
|  | Adjusted Odds Ratio of Falls Resulting in Time-Loss (adjusted for subsector, gender, age, employment status and occupation) | | | | | | | | | | | | | | | | | 1.00 (ref) | | | | | | | 0.73  (0.36–1.48) | | | | | | | | | | | | | | 1.72  (1.06–2.81)* | |  |
|  | Rates for MSIs (***P<0.001) per 100 person-years | | | | | | | | | | | | | | | | | 4.6 p-y | | | | | | | 9.2 p-y | | | | | | | | | | | | | | 16.4 p-y | | ^64^ |
|  | Adjusted Relative Risk (adjusted for gender, age group, sub-sector, employment status and occupation) | | | | | | | | | | | | | | | | | 1.00 (ref.) | | | | | | | 1.98  (1.49-2.63) *** | | | | | | | | | | | | | | 3.76  (3.09-4.59) *** | |  |
|  | Rates for All Injuries per 100 person-years | | | | | | | | | | | | | | | | | 5.1p-y | | | | | | | 10.3 p-y | | | | | | | | | | | | | | 19.5 p-y | |  |
|  | Adjusted Relative Risk (adjusted for gender, age group, sub-sector, employment status and occupation) | | | | | | | | | | | | | | | | | 1.00 (ref.) | | | | | | | 1.98  (1.54-2.55) *** | | | | | | | | | | | | | | 3.62  (3.03-4.33) *** | |  |
| **Rate by Employment Status** | Rates for All injuries | | | | | | | | | | | | | | | **per 100 person years** | | | | | | | | **Adjusted Relative Risk (adjusted for age and gender)** | | | | | | | | | | | | | | | | | ^62^ |
|  |  | | | | | **CA** | | | | | FT | | | | | 25.8 | | | | | | | | 25.8/ 1.0 (ref.) | | | | | | | | | | | | | | | | |  |
|  |  |  |  |  |  |  | | | | | PT | | | | | 22.9 | | | | | | | | 25.8/ 1.0 (ref.) | | | | | | | | | | | | | | | | |  |
|  |  |  |  |  |  |  | | | | | Casual | | | | | 18.1 | | | | | | | | 25.8/ 1.0 (ref.) | | | | | | | | | | | | | | | | |  |
|  |  |  |  |  |  | **RN** | | | | | FT | | | | | 7.4 | | | | | | | | 1.00 (ref.) | | | | | | | | | | | | | | | | |  |
|  |  |  |  |  |  |  | | | | | PT | | | | | 5.3 | | | | | | | | 0.7 (0.6, 0.9)* | | | | | | | | | | | | | | | | |  |
|  |  |  |  |  |  |  | | | | | Casual | | | | | 5.5 | | | | | | | | 0.7 (0.5, 1.0)* | | | | | | | | | | | | | | | | |  |
|  | Rates for MSIs | | | | | | | | | | FT | | | | | 20.8 | | | | | | | | 1.0 (ref.) | | | | | | | | | | | | | | | | |  |
|  |  |  |  |  |  |  |  |  |  |  | PT | | | | | 19.3 | | | | | | | | 0.9 (0.6, 1.3) | | | | | | | | | | | | | | | | |  |
|  |  |  |  |  |  |  |  |  |  |  | Casual | | | | | 15.2 | | | | | | | | 0.6 (0.5, 0.8)* | | | | | | | | | | | | | | | | |  |
| **Rate by Setting** | All Injury Rates (per 100 FTE) | | | | | | | | | | | | | | | | **Acute Care** | | | | | | | | **Community** | | | | | | | | | | | | | | **LTC** | | ^60^ |
|  |  | | | | | RN | | | | | | | | | | | 21.9 | | | | | | | | 7.7 | | | | | | | | | | | | | | 17.2 | |  |
|  |  | | | | | LPN | | | | | | | | | | | 30.5 | | | | | | | | No data | | | | | | | | | | | | | | 26.8 | |  |
|  |  | | | | | CA | | | | | | | | | | | 30.7 | | | | | | | | 25.3 | | | | | | | | | | | | | | 37.0 | |  |
|  | Time-loss Falls for RNs, LPNs, and CAs (95%CI) | | | | | Crude Rate | | | | | | | | | | | 1.00 (ref) | | | | | | | | 1.17  (0.82–1.67) | | | | | | | | | | | | | | 2.09  (1.38–3.17)** | | ^61^ |
|  |  |  |  |  |  | Adjusted Odds Ratio | | | | | | | | | | | 1.00 (ref) | | | | | | | | 0.67  (0.40–1.11) | | | | | | | | | | | | | | 1.71  (1.06–2.75)* | |  |
| **Loss and Costs due to Injury and Sickness Time** | Median Days Lost/Fall Claim (interquartile range for 2005-2008) | | | | | | | | | | | | | | | | | **RN** | | | | | | | **LPN** | | | | | | | | | | | | | | **CA** | | ^61^ |
|  |  |  |  |  |  |  |  |  |  |  |  |  |  |  |  |  |  | 14 (3-65) | | | | | | | 27 (6-102) | | | | | | | | | | | | | | 27 (6-102) | |  |
|  | Rate of time loss falls/100 FTE | | | | | | | | | | | | | | | | | 0.9 | | | | | | | 0.9 | | | | | | | | | | | | | | 2.2 | |  |
|  | Sick days/ person year of productive hours | | | | | | | | | | | | | | | | | 15.5 | | | | | | | 19.5 | | | | | | | | | | | | | | 19.5 | | ^63^ |
|  | Median Cost per Fall Claim (interquartile range) (CDN$)(2005-2008) | | | | | | | | | | | | | | | | | 2065  (682-8367) | | | | | | | 2342  (622-11339) | | | | | | | | | | | | | | 2144  (724-8673) | | ^61^ |
|  | Total Sickness Costs per person year of productive hours (CDN$) | | | | | | | | | | | | | | | | | 3667 | | | | | | | 3042 | | | | | | | | | | | | | | 2763 | | ^63^ |
|  | Total annual costs due to sickness absence (CDN$) | | | | | | | | | | | | | | | | | 21,896,334 | | | | | | | 3,085,709 | | | | | | | | | | | | | | 7,830,904 | |  |
|  | Falls resulting in compensation for wage-loss claims | | | | | | | | | | | | | | | | | 58% | | | | | | | 55% | | | | | | | | | | | | | | 75% | | ^61^ |
|  | Mean Days Unable to work due to injury | | | | | | | | | | | | | | | | |  | | | | | | |  | | | | | | | | | | | | | | 3.01 (0.54) | | ^24^ |
|  | Days unable to work due to Injury (weighted %)(SE) | | | | | | | | | | | | | | | | | 0 | | | 1 | | | | | | | 2 | | | | | | | >3 | | | | | |  |
|  |  | | | | | | | | | | | | | | | | | 74.63 (2.23) | | | 4.56 (0.73) | | | | | | | 3.85  (0.59) | | | | | | | 15.36(2.7) | | | | | |  |
| **Factors Associated with Injury** | Organization | | | | Facility | | | | | | | | | | | | | | | | | | | | | | | | | | | | | | | | | | | | ^90^ |
|  |  |  |  |  | More Likely to Report High Injury Rate (AOR) | | | | | | | | | | | | Chain member | | | | | | | | 38% | | | | | | | | | | | | | | 1.38, 1.22–1.55 ** | |  |
|  |  |  |  |  |  |  |  |  |  |  |  |  |  |  |  |  | High Average Occupancy Rate | | | | | | | |  | | | | | | | | | | | | | | 1.18, 1.11-1.26 ** | |  |
|  |  |  |  |  | Less Likely to Report High Injury Rate | | | | | | | | | | | | For Profit | | | | | | | | 26% | | | | | | | | | | | | | | 0.84, 0.74–0.9 ** | |  |
|  |  |  |  |  |  |  |  |  |  |  |  |  |  |  |  |  | High FTE NA pre 100 beds | | | | | | | |  | | | | | | | | | | | | | | 0.95, 0.89–1.01 ** | |  |
|  | Unit | | | | Training | | | | | | Odds Ratio (Marginal effect) | | | | | | | | | | | | | | | | | | | | | | | | | | | | | | ^50^ |
|  |  |  |  |  |  |  |  |  |  |  | Training to prevent workplace injuries during initial NA training was excellent | | | | | | | | | | | | | 0.81(0.13) | | | | | | | | | | | | | | | | |  |
|  |  |  |  |  |  |  |  |  |  |  | Facility provides training to reduce WPIs | | | | | | | | | | | | | 0.61(0.16)* | | | | | | | | | | | | | | | | |  |
|  |  |  |  |  |  |  |  |  |  |  | Lifting device always available with need | | | | | | | | | | | | | 0.59 (0.12)** | | | | | | | | | | | | | | | | |  |
|  |  |  |  |  |  |  |  |  |  |  | Enough time available to give ADLs to residents in typical work week | | | | | | | | | | | | | 0.65(0.11)** | | | | | | | | | | | | | | | | |  |
|  |  |  |  |  |  |  |  |  |  |  | Time at current facility 4 or less months | | | | | | | | | | | | | 0.203 (0.08)** | | | | | | | | | | | | | | | | |  |
|  |  |  |  |  |  |  |  |  |  |  | Time at current facility 5 – 8 months | | | | | | | | | | | | | 0.43(0.10)** | | | | | | | | | | | | | | | | |  |
|  |  |  |  |  |  |  |  |  |  |  | Time at current facility 9-11 months | | | | | | | | | | | | | 0.61 (0.21) | | | | | | | | | | | | | | | | |  |
|  |  |  |  |  |  |  |  |  |  |  | < 1 year as NA | | | | | | | | | | | | | 1.87 (0.55)* | | | | | | | | | | | | | | | | |  |
|  |  |  |  |  |  |  |  |  |  |  | 1 year to < 2 year 1.842 | | | | | | | | | | | | | 1.84(0.50)* | | | | | | | | | | | | | | | | |  |
|  |  |  |  |  |  |  |  |  |  |  | 2-5 years 1.518 | | | | | | | | | | | | | 1.52 (0.30)* | | | | | | | | | | | | | | | | |  |
|  |  |  |  |  | **HHAs Ratings of Training by Number of Injuries Since Starting their Position or in the Last 12 Months n=3375 (%)** | | | | | | | | | | | | | | | | | | | 1 injury | | | | | | | | 2 | | | | | | 3 | | | ^94^ |
|  |  |  |  |  |  | | Odds of injury among HHAs feeling somewhat prepared by their training (vs. well-prepared) | | | | | | | | | | | | | | | | | 1.10 | | | | | | | | 1.83* | | | | | | 2.41* | | |  |
|  |  |  |  |  |  |  | Odds of injury among HHAs who did not feel prepared (vs. well-prepared) by their training | | | | | | | | | | | | | | | | | 3.05** | | | | | | | | 4.70* | | | | | | 8.09** | | |  |
|  |  |  |  |  |  |  | Odds of injury among HHAs with poor support from their supervisor (vs. good support) | | | | | | | | | | | | | | | | | 1.51* | | | | | | | | 1.39 | | | | | | 3.10** | | |  |
|  |  |  |  |  | Leadership Style | | | | | | TL negatively associated with number of injuries (B) | | | | | | | | | | | | | -0.17 ** | | | | | | | | | | | | | | | | | ^51^ |
|  |  |  |  |  |  |  |  |  |  |  | TL positively associated with injury-related absenteeism | | | | | | | | | | | | | -3.65* | | | | | | | | | | | | | | | | |  |
|  |  |  |  |  | General | | | | | |  | | | | | | | | | | | | | **%** | | | | | | | | | | | | | | | | | ^64^ |
|  |  |  |  |  |  |  |  |  |  |  | Ergonomics | | | | | | | | |  | | | | 55 | | | | | | | | | | | | | | | | |  |
|  |  |  |  |  |  |  |  |  |  |  | Awkward posture | | | | | | | | |  | | | | 25 | | | | | | | | | | | | | | | | |  |
|  |  |  |  |  |  |  |  |  |  |  | Force | | | | | | | | |  | | | | 23 | | | | | | | | | | | | | | | | |  |
|  |  |  |  |  |  |  |  |  |  |  | Slip and Fall | | | | | | | | |  | | | | 12 | | | | | | | | | | | | | | | | |  |
|  |  |  |  |  |  |  |  |  |  |  | Miscellaneous | | | | | | | | |  | | | | 17 | | | | | | | | | | | | | | | | |  |
|  |  |  |  |  |  |  |  |  |  |  | Workplace Aggression | | | | | | | | | | | | | 11 | | | | | | | | | | | | | | | | |  |
|  |  |  |  |  |  |  |  |  |  |  | Equipment | | | | | | | | |  | | | | 4 | | | | | | | | | | | | | | | | |  |
|  |  |  |  |  |  |  |  |  |  |  | MVA | | | | | | | | |  | | | | 1 | | | | | | | | | | | | | | | | |  |
|  |  |  |  |  |  |  |  |  |  |  | Aggressive clients or family members, discrimination, racism, and unsafe conditions | | | | | | | | | | | | | | | | | | | | | | | | | | | | | | ^44^ |
|  |  |  |  |  |  |  |  |  |  |  | Poor working conditions can also impact workers’ physical health and have been linked to MSI | | | | | | | | | | | | | | | | | | | | | | | | | | | | | |  |
|  | Individual | | | | **Occupational Activity leading to MSIs (%)** | | | | | | | | | | | | | | | | | | | | | | | | | | **n** | | | | | **%** | | | | | ^64^ |
|  |  |  |  |  |  | | | Patient handling | | | | | | | | | | | | | | | | | | | | | | | 369 | | | | | 59 | | | | |  |
|  |  |  |  |  |  |  |  |  | | Repositioning | | | | | | | | | | | | | | | | | | | | | 137 | | | | |  | | | | |  |
|  |  |  |  |  |  |  |  |  |  | Transferring | | | | | | | | | | | | | | | | | | | | | 149 | | | | |  | | | | |  |
|  |  |  |  |  |  |  |  |  |  | Preventing a Fall | | | | | | | | | | | | | | | | | | | | | 55 | | | | |  | | | | |  |
|  |  |  |  |  |  |  |  |  |  | Holding/Assisting during a procedure | | | | | | | | | | | | | | | | | | | | | 70 | | | | |  | | | | |  |
|  |  |  |  |  |  |  |  |  |  | Assisted walking | | | | | | | | | | | | | | | | | | | | | 12 | | | | |  | | | | |  |
|  |  |  |  |  |  |  |  |  |  | Toileting | | | | | | | | | | | | | | | | | | | | | 13 | | | | |  | | | | |  |
|  |  |  |  |  |  |  |  | Patient care | | | | | | | | | | | | | | | | | | | | | | | 84 | | | | | 14 | | | | |  |
|  |  |  |  |  |  |  |  |  | | Personal Care | | | | | | | | | | | | | | | | | | | | | 79 | | | | |  | | | | |  |
|  |  |  |  |  |  |  |  |  | |  | | Washing | | | | | | | | | | | | | | | | | | | 45 | | | | |  | | | | |  |
|  |  |  |  |  |  |  |  |  | |  | | Dressing | | | | | | | | | | | | | | | | | | | 22 | | | | |  | | | | |  |
|  |  |  |  |  |  |  |  |  | |  | | Changing | | | | | | | | | | | | | | | | | | | 34 | | | | |  | | | | |  |
|  |  |  |  |  |  |  |  |  | |  | | Feeding | | | | | | | | | | | | | | | | | | | 1 | | | | |  | | | | |  |
|  |  |  |  |  |  |  |  |  | | Procedure | | | | | | | | | | | | | | | | | | | | | 5 | | | | |  | | | | |  |
|  |  |  |  |  | **Blood and Body Fluid Contact Among PCAs (n=980)** | | | | | | | | | | | | | | | | | | | | | | | | | | **n** | | | | | **%** | | | | | ^73^ |
|  |  |  |  |  |  | | | Change Dirty Linens | | | | | | | | | | | | | | | | | | | | | | | 700 | | | | | 79.0 | | | | |  |
|  |  |  |  |  |  |  |  | Change Wound Dressing | | | | | | | | | | | | | | | | | | | | | | | 95 | | | | | 11.0 | | | | |  |
|  |  |  |  |  |  |  |  | Empty Wound Drainage | | | | | | | | | | | | | | | | | | | | | | | 43 | | | | | 5.1 | | | | |  |
|  |  |  |  |  |  |  |  | Handle Sharps | | | | | | | | | | | | | | | | | | | | | | | 119 | | | | | 13.8 | | | | |  |
|  |  |  |  |  |  |  |  | Insert/Care of Urinary Catheter | | | | | | | | | | | | | | | | | | | | | | | 35 | | | | | 4.1 | | | | |  |
|  |  |  |  |  |  |  |  | Colostomy Care | | | | | | | | | | | | | | | | | | | | | | | 38 | | | | | 4.5 | | | | |  |
|  |  |  |  |  |  |  |  | Tracheostomy Care | | | | | | | | | | | | | | | | | | | | | | | 36 | | | | | 4.2 | | | | |  |
|  |  |  |  |  | **Odds Ratio of Reported Activities Associated with Blood and Body Fluid Contact** | | | | | | | | | | | | | | | | | | **RN** | | | | | | | | | | **PCA** | | | | | | | |  |
|  |  |  |  |  |  | | Change Dirty Linens | | | | | | | | | | | | | | | | 1.7 (1.2-2.4) | | | | | | | | | | 1.6 (0.7-3.4) | | | | | | | |  |
|  |  |  |  |  |  |  | Change Wound Dressing | | | | | | | | | | | | | | | | 2.5 (1.6-4.1) | | | | | | | | | | 6.3(3.4-11.6) | | | | | | | |  |
|  |  |  |  |  |  |  | Empty Wound Drainage | | | | | | | | | | | | | | | | 2.1(1.4-3.1) | | | | | | | | | | 8.5(4.0-18.0) | | | | | | | |  |
|  |  |  |  |  |  |  | Handle Sharps | | | | | | | | | | | | | | | | 1.4(0.9-2.1) | | | | | | | | | | 7.4(4.1-13.3) | | | | | | | |  |
|  |  |  |  |  |  |  | Insert/Care of Urinary Catheter | | | | | | | | | | | | | | | | 1.6  (1.1-2.3) | | | | | | | | | | 6.9  (3.0-15.7) | | | | | | | |  |
|  |  |  |  |  |  |  | Colostomy Care | | | | | | | | | | | | | | | | 1.6 (1.1-2.3) | | | | | | | | | | 4.9(2.1-11.2) | | | | | | | |  |
|  |  |  |  |  |  |  | Tracheostomy Care | | | | | | | | | | | | | | | | 1.6 (1.1-2.3) | | | | | | | | | | 2.9 (1.1-7.9) | | | | | | | |  |
|  |  |  |  |  | **Consequences of HHA Worker Injury Rates (B co-efficient)** | | | | | | | | | | | | | | | | | | | | | | | | | | | | | | | | | | | | ^94^ |
|  |  |  |  |  |  | | Decrease Job Satisfaction | | | | | | | | | | | | | | | | -0.119 ** | | | | | | | | | | | | | | | | | |  |
|  |  |  |  |  |  |  | Increase Turnover Intent | | | | | | | | | | | | | | | | +0.069** | | | | | | | | | | | | | | | | | |  |
|  |  |  |  |  |  |  | Willingness to recommend agency for job | | | | | | | | | | | | | | | | -0.11** | | | | | | | | | | | | | | | | | |  |
|  |  |  |  |  |  |  | Willingness to recommend agency for care | | | | | | | | | | | | | | | | -0.099** | | | | | | | | | | | | | | | | | |  |
| **Rate by Individual Characteristics**  Relative Risk (RR)  Injury Rates/100 FTEs | Age | | | | | | | <30 | | | | | 30-39 | | | | | | <40 | | | | | | | | 40-49 | | | | | | | | | | | | 50-59 | >60 |  |
|  |  | MSI | | | | | | n/a | | | | | | | | | | | 0.5 (0.3- 1.0) * | | | | | | | | n/a | | | | | | | | | | | | n/a | n/a | ^62^ |
|  |  | All Injury | | | | | | n/a | | | | | | | | | | | 0.5 (0.3- 0.9)* | | | | | | | | n/a | | | | | | | | | | | | n/a | n/a |  |
|  |  | Acute Care | | | | | | 43.5 | | | | | 34.0 | | | | | | n/a | | | | | | | | 32.4 | | | | | | | | | | | | 27.5 | 21.0 | ^60^ |
|  |  | Community | | | | | | 23.1 | | | | | 26.5 | | | | | | n/a | | | | | | | | 26.2 | | | | | | | | | | | | 24.3 | 26.1 |  |
|  |  | Long-term Care | | | | | | 49.5 | | | | | 42.1 | | | | | | n/a | | | | | | | | 35.7 | | | | | | | | | | | | 37.8 | 15.9 |  |
|  | Gender | | |  | | | | | | | | | | | | | | | | | | **Female** | | | | | | | | | | | | | | | **Male** | | | |  |
|  |  |  |  |  |  |  |  |  |  |  |  |  |  |  |  |  |  |  |  |  |  | 38.8/100FTE | | | | | | | | | | | | | | | 15.7/100 FTE | | | | ^60^ |
|  |  |  |  | MSI(per 100 person-years) (adjusted for age and gender) | | | | | | | | | | | FT | | | | | | | 27.6 | | | | | | | | | | | | | | | 5.2 | | | | ^62^ |
|  |  |  |  |  |  |  |  |  |  |  |  |  |  |  | PT | | | | | | | 23.5 | | | | | | | | | | | | | | | 14.3 | | | |  |
|  |  |  |  |  |  |  |  |  |  |  |  |  |  |  | Casual | | | | | | | 18.4 | | | | | | | | | | | | | | | 14.3 | | | |  |
|  |  |  |  | All Injury(per 100 person-years) (adjusted for age and gender) | | | | | | | | | | | FT | | | | | | | 22.4 | | | | | | | | | | | | | | | 3.4 | | | |  |
|  |  |  |  |  |  |  |  |  |  |  |  |  |  |  | PT | | | | | | | 19.7 | | | | | | | | | | | | | | | 14.3 | | | |  |
|  |  |  |  |  |  |  |  |  |  |  |  |  |  |  | Casual | | | | | | | 15.3 | | | | | | | | | | | | | | | 14.3 | | | |  |
|  |  |  |  | MSI(per 100 person-years) (adjusted for age and gender) | | | | | | | | | | | PT vs. FT | | | | | | | 0.8 (0.6-1.2) | | | | | | | | | | | | | | | 4.0 (0.9, 17.4)* | | | |  |
|  |  |  |  |  |  |  |  |  |  |  |  |  |  |  | Casual vs. FT | | | | | | | 0.6 (0.4-0.8)* | | | | | | | | | | | | | | | 3.5 (1.0, 13.1)* | | | |  |
|  |  |  |  | All Injury(per 100 person-years) (adjusted for age and gender) | | | | | | | | | | | PT vs. FT | | | | | | | 0.8 (0.6-1.1) | | | | | | | | | | | | | | | 2.7 (1.0-7.1)* | | | |  |
|  |  |  |  |  |  |  |  |  |  |  |  |  |  |  | Casual vs. FT | | | | | | | 0.6 (0.5-0.8)* | | | | | | | | | | | | | | | 2.4 (0.8-7.6) | | | |  |
